# Magnetic Field‐Driven Spin State Transformation in Promoting the Catalytic Activity of Doped Single‐Atom for Hydrogen Evolution Reaction

**DOI:** 10.1002/adma.202513213

**Published:** 2025-11-07

**Authors:** Chenjing Wang, Yuquan Yang, Jinlong Zheng, Yanru Yuan, Dawei Pang, Jiajia Liu, Hongjing Wu, Naiyan Liu, Hui Ying Yang, Xiaolu Pang

**Affiliations:** ^1^ Beijing Advanced Innovation Center for Materials Genome Engineering School of Advanced Materials Innovation University of Science and Technology Beijing Beijing 100083 China; ^2^ Shunde Innovation School University of Science and Technology Beijing Foshan 528399 China; ^3^ College of Materials Science and Engineering Beijing University of Technology Beijing 100083 China; ^4^ Department of Materials Science and Engineering College of Design and Engineering National University of Singapore 9 Engineering Drive 1 Singapore 117575 Singapore; ^5^ School of Materials Science and Engineering State Key Laboratory of Nuclear Power Safety Technology and Equipment University of Science and Technology Beijing Beijing 100083 China

**Keywords:** electronic structure modulation, external magnetic field, hydrogen evolution reaction, single‐atom Ru‐doping, spin state transformation

## Abstract

Developing efficient electrocatalysts for the hydrogen evolution reaction (HER) requires innovative strategies to modulate electronic structures and reaction kinetics. Herein, a ferromagnetic Ru_SAs_/Ni_2_P@Fe_3_O_4_ core‐shell catalyst is designed, which synergizes Ru single‐atoms (SAs) doping and external magnetic field excitation. Under a 0.3 T magnetic field, Ru_SAs_/Ni_2_P@Fe_3_O_4_−0.3 T achieves a remarkably low overpotential of 38.9 mV at 10 mA cm^−2^ and a Tafel slope of 39.5 mV dec^−1^ in alkaline media, outperforming its counterparts without magnetic stimulation. Advanced characterization (XANES, Mössbauer, EPR, SQUID) and density functional theory calculations reveal that the magnetic field induces a spin‐state transition in Fe^3+^ (from low‐spin to high‐spin), enhancing interfacial charge transfer and enriching electron density around Ru SAs. These effects optimize hydrogen adsorption free energy (ΔG_H*_) and reaction kinetics. The Ru SAs serve as the dominant active sites, while the spin‐state reconfiguration of the Fe_3_O_4_ core under magnetic fields stabilizes the structure and accelerates electron transfer. This work unveils a dual‐regulation mechanism combining atomic doping and spin engineering, offering a novel pathway for designing high‐performance catalysts via electronic and magnetic synergy.

## Graphical abstract

A ferromagnetic Ru_SAs_/Ni_2_P@Fe_3_O_4_ material is designed via a synergistic strategy of single‐atom doping and magnetic field‐driven spin‐state transformation. This strategy introduces new Ru single‐atom active sites for the hydrogen evolution reaction and facilitates the transition of Fe^3+^ (3d t_2g_) low‐spin electrons to a high‐spin state (3d e_g_). Consequently, electron transfer to the Ru single‐atom is enhanced, boosting overall catalytic efficiency.

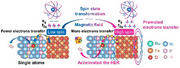

## 1 Introduction

**Figure 1:**
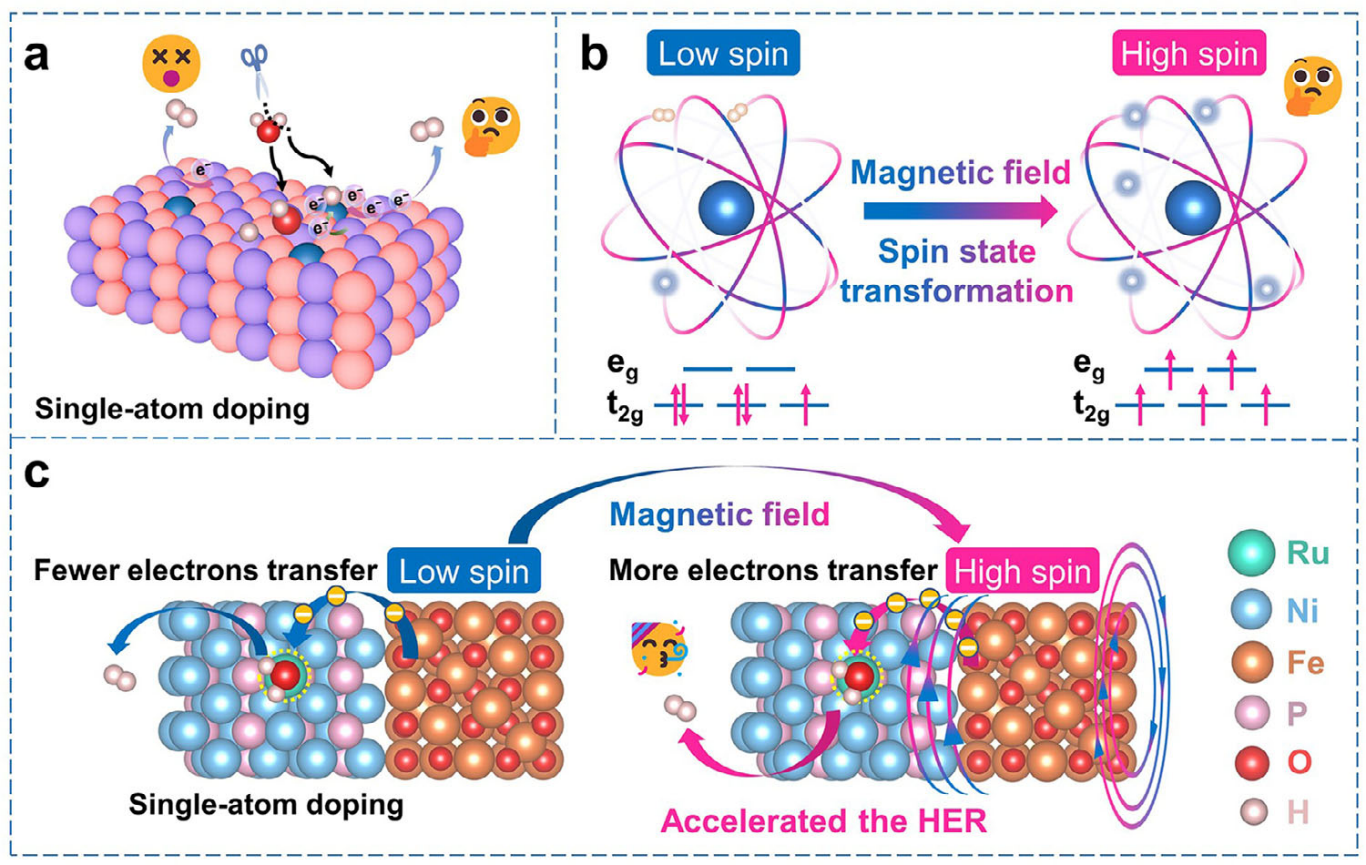
Schematic diagram of the process in which single‐atom doping, spin state transformation driven by magnetic field, and their synergistic effect promote the HER reaction. a) Schematic diagram of the mechanism of electrocatalytic HER reaction enhanced by SAs doping. b) Schematic diagram of the promotion of interfacial electron transfer by spin state transformation dominated by an external magnetic field. c) Schematic diagram of the process in which the synergistic effect of SAs doping and spin state transformation driven by an external magnetic field accelerates the HER.

Due to their high atomic efficiency and remarkable catalytic activity, atomically dispersed single‐atom catalysts (SACs) have gained significant attention in various electrocatalytic reactions in recent years.^[^ ^1^, ^2^, ^3^, ^4^, ^5^ ^]^ In the alkaline electrocatalytic water decomposition process, water molecules first undergo the Volmer step (H_2_O + e^−^ + * → H_ads_ + OH^−^) to achieve water dissociation and generate adsorbed hydrogen. Subsequently, the adsorbed hydrogen is released as hydrogen molecules through the desorption process (Heyrovsky step: H_ads_ + H_2_O + e^−^ → H_2_ + OH^−^ + *). However, the significant energy barrier in the water dissociation process and the low efficiency of hydrogen desorption both impede the activity of the hydrogen evolution reaction (HER).^[^ ^6^ ^]^ To address this core bottleneck, recent studies have made breakthroughs by precisely regulating the electronic structure of active sites. For instance, Yu et al.^[^ ^7^ ^]^ utilized the Mott‐Schottky effect on the surface of intermetallic alloys to construct high‐density Frustrated Lewis pairs (FLP). These FLP sites synergistically optimize the electron transfer efficiency of key HER steps (water adsorption and hydrogen desorption), significantly reducing the reaction energy barrier and offering a core idea for regulating the electronic state of active sites for enhancing alkaline HER activity. The optimization of electronic properties of the catalyst is considered a viable method to enhance HER performance.^[^ ^8^ ^]^ Doping single‐atoms (SAs) into nanomaterials can improve electron transfer and decrease energy barriers, thereby boosting the HER rate (**Figure** **1**a). For instance, Zhu et al.^[^ ^9^ ^]^ introduced Pt SAs into Ru/RuO_2_, which not only created new active sites but also altered the electron density, significantly enhancing the H atom combination step and resulting in superior alkaline HER activity. Additionally, Ru SAs are recognized as excellent catalysts due to their high atomic utilization and unique quantum size effect,^[^ ^10^, ^11^ ^]^ making them suitable for enhancing HER activity when incorporated into transition metal phosphides. Thus, doping Ru SAs into these materials presents a promising strategy for improving HER catalytic performance.

In addition to enhancing the intrinsic activity of catalysts, external excitation enhancement strategies have emerged as a new trend in the field of electrochemistry. Approaches such as magnetic fields,^[^ ^12^, ^13^ ^]^ light fields,^[^ ^14^ ^]^ heat fields,^[^ ^15^ ^]^ and ultrasound^[^ ^16^ ^]^ effectively alter reaction kinetics. Recent studies have shown that external magnetic fields, as pollution‐free and non‐contact technologies, have contributed significantly to efficient hydrogen production.^[^ ^17^ ^]^ In electrocatalytic processes, external magnetic fields not only affect reaction kinetics through physical effects (e.g., magnetic force‐induced mass transfer) but also induce spin transitions in ferromagnetic materials, thereby enhancing electron transfer efficiency and catalytic performance (Figure 1b).^[^ ^18^ ^]^ However, existing studies have shown that magnetic force‐induced mass transfer has a weak impact on catalytic reactions. For instance, Ren et al.^[^ ^19^ ^]^ placed paramagnetic materials IrO_2_ and Co_3_O_4_ in a magnetic field for electrocatalytic reactions and found that the improvement in their catalytic performance was not significant. Additionally, based on the analysis of Grotthuss' theory,^[^ ^20^ ^]^ proton transport in aqueous solutions does not involve physical movement but is achieved through continuous proton transfer. Thus, magnetic fields cannot significantly enhance catalytic performance by promoting mass transfer (e.g., accelerating ion convection). In contrast, the role of magnetic fields in inducing spin state transitions in ferromagnetic materials is more critical. For instance, Li et al.^[^ ^21^ ^]^ demonstrated that orbital spin splitting facilitated the transition of the electronic structure in FeCoPS_3_ from a low‐spin (LS) state to a high‐spin (HS) state. This spin state transition enhanced the charge flow and orbital orientation, thereby reducing the energy barrier and strengthening the reaction kinetics, suggesting that the magnetic field contributes to its catalytic activity. Fe_3_O_4_ is a common ferromagnetic material,^[^ ^22^ ^]^ and electrons can be excited from a LS state to a HS state by an external magnetic field. Magnetic field intensity, as one of the core parameters, directly determines the degree of spin polarization. Currently, studies have shown that under a magnetic field of 0.1–0.6 T, the overpotential of HER can be significantly reduced and ferromagnetic materials undergo spin‐state transitions.^[^ ^23^, ^24^ ^]^ It is worth emphasizing that different magnetic field intensities exhibit remarkable differences in their effects on the catalytic system.^[^ ^25^ ^]^ Such a spin state transformation can enhance the synergy between Fe_3_O_4_ and other catalyst components, thereby constructing an efficient electrocatalytic system.

Based on the above analysis, combining SACs with an external magnetic field may induce a synergistic effect, further enhancing electrocatalytic performance (Figure 1c). When a magnetic field is introduced into a transition metal phosphide system doped with SAs, on the one hand, the new active sites and unique electronic structure introduced by SAs can optimize catalyst‐reactant interactions and reduce the reaction energy barrier.^[^ ^26^ ^]^ On the other hand, the external magnetic field can modulate the electron spin state and accelerate electron transfer, with the two effects complementing each other. This synergistic effect may enable the Volmer step in the water dissociation process and the Heyrovsky step of hydrogen desorption to proceed more efficiently, significantly enhancing HER activity and even potentially surpassing the performance limits of SACs or magnetic fields acting alone.

Inspired by the aforementioned research achievements, this study combines the inherent activity of the catalyst with an external excitation approach, proposing a novel strategy to enhance the catalytic efficiency of the Ru_SAs_/Ni_2_P@Fe_3_O_4_ heterostructure through an external magnetic field. In this structure, Ru SAs are uniformly dispersed on the Ni_2_P surface, with Fe_3_O_4_ acting as a stable substrate. Due to its ferromagnetic characteristics, Fe_3_O_4_ is highly responsive to the external magnetic field. Density functional theory (DFT) calculations reveal that application of the magnetic field shifts Fe_3_O_4_ from a LS to a HS state, promoting electron transfer from Fe_3_O_4_ to the Ru_SAs_/Ni_2_P interface and increasing electron density at the Ru SAs. This electron‐dense environment enhances the interaction between Ru and Ni_2_P, leading to robust electronic coupling. During hydrogen adsorption and desorption, this coupling allows the abundant electrons around Ru SAs to effectively stabilize adsorbed hydrogen and lower the energy barrier for hydrogen adsorption. Under a 0.3 T external magnetic field, the Ru_SAs_/Ni_2_P@Fe_3_O_4_ catalyst achieves a current density of 10 mA cm^−2^ at a low overpotential of 38.9 mV, with a favorable Tafel slope of 39.5 mV dec^−1^. Furthermore, after 48 h of continuous operation at −0.039 V vs RHE, the catalyst exhibits a minimal performance degradation rate of just 1.5%. Such structural and electronic property changes induced by Ru SAs, combined with magnetic field‐induced modulation of the spin state of Fe_3_O_4_, construct an efficient synergistic catalytic mechanism. This lays a solid experimental and theoretical foundation for the Ru_SAs_/Ni_2_P@Fe_3_O_4_ heterostructure exhibiting superior performance in the electrocatalytic HER.

## 2 Results and Discussion

### 2.1 Synthesis and Characterization

**Figure 2:**
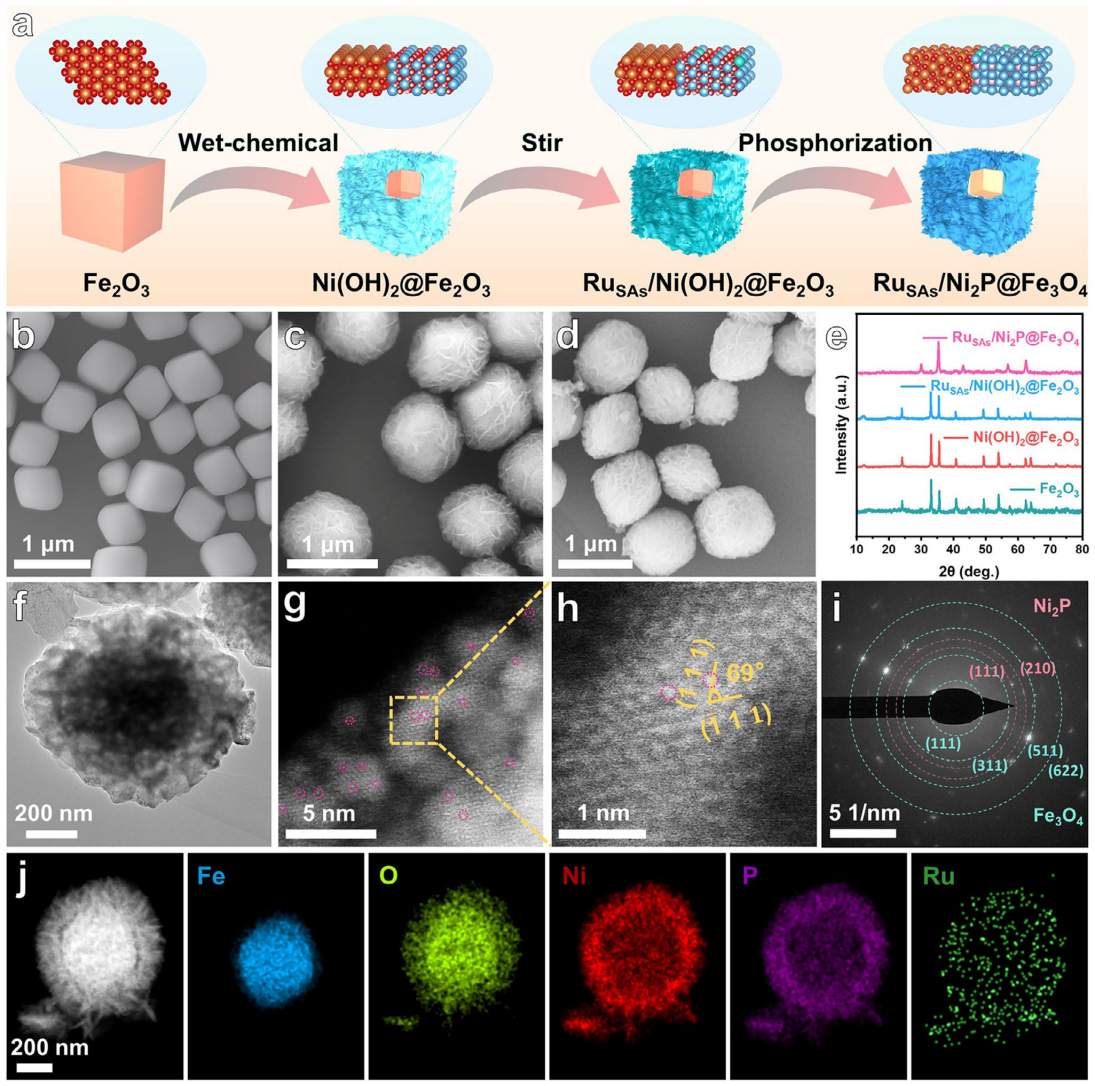
Synthesis process and structural characterizations of Ru_SAs_/Ni_2_P@Fe_3_O_4_. a) Schematic illustration for the preparation of Ru_SAs_/Ni_2_P@Fe_3_O_4_ core‐shell structure. SEM images of b) Fe_2_O_3_, c) Ru_SAs_/Ni(OH)_2_@Fe_2_O_3_, and d) Ru_SAs_/Ni_2_P@Fe_3_O_4_. XRD patterns of e) Fe_2_O_3_, Ni(OH)_2_@Fe_2_O_3_, Ru_SAs_/Ni(OH)_2_@Fe_2_O_3_ and Ru_SAs_/Ni_2_P@Fe_3_O_4_. f) TEM and g,h) AC HAADF‐STEM images of Ru_SAs_/Ni_2_P@Fe_3_O_4_. i) SAED pattern of Ru_SAs_/Ni_2_P@Fe_3_O_4_, with its polycrystalline rings corresponding to Fe_3_O_4_ (cyan rings) and Ni_2_P (bright pink rings). j) HAADF‐STEM image of Ru_SAs_/Ni_2_P@Fe_3_O_4_, along with related EDS mapping images of Fe, O, Ni, P, and Ru elements.

**Figure** **2**a depicts the synthesis of the Ru_SAs_/Ni_2_P@Fe_3_O_4_ core‐shell structure. First, Fe_2_O_3_ was synthesized via a hydrothermal method. X‐ray diffraction (XRD) confirmed its cubic structure (JCPDS No. 00‐033‐0664) (Figure 2e; Figure S1, Supporting Information).^[^ ^27^ ^]^ The scanning electron microscopy (SEM) image revealed that the size of the Fe_2_O_3_ precursor is ≈400 nm (Figure 2b). Next, Ni(OH)_2_ nanosheets were deposited onto the Fe_2_O_3_ surface via a wet chemical method, resulting in the Ni(OH)_2_@Fe_2_O_3_ nanomaterial.

The XRD pattern of this composite matched the phases of both Ni(OH)_2_ (JCPDS No. 00‐038‐0715)^[^ ^28^ ^]^ and Fe_2_O_3_ (Figure 2e; Figure S2, Supporting Information). Additionally, the SEM image in Figure S3 (Supporting Information) demonstrates uniform deposition of Ni(OH)_2_ nanosheets on the Fe_2_O_3_, forming the core‐shell structure. Ru atoms were subsequently incorporated into the Ni(OH)_2_@Fe_2_O_3_ via stirring. XRD results indicate that the Ru‐doped sample preserves the characteristic diffraction peaks of Ni(OH)_2_@Fe_2_O_3_, implying minimal alteration of the crystal structure upon Ru doping (Figure 2e; Figure S4, Supporting Information). Notably, no diffraction peaks for Ru or RuO_2_ are observed, confirming the existence of Ru SAs. This sample was designated as Ru_SAs_/Ni(OH)_2_@Fe_2_O_3_. The SEM image indicates that the morphology of Ru_SAs_/Ni(OH)_2_@Fe_2_O_3_ remains consistent with the undoped material (Figure 2c). Finally, Ru_SAs_/Ni(OH)_2_@Fe_2_O_3_ was phosphorized using NaH_2_PO_2_ in a tube furnace, fully converting Ni(OH)_2_ to Ni_2_P and Fe_2_O_3_ to Fe_3_O_4_. The Inductively Coupled Plasma Optical Emission Spectrometry (ICP─OES) analysis (Table S1, Supporting Information) confirms a Ru mass fraction of 1.61 wt% in the phosphatized sample.^[^ ^29^ ^]^ XRD analysis (Figure 2e; Figure S5, Supporting Information) shows that the phosphorized material corresponds to the Ni_2_P (JCPDS No. 00‐003‐0953)^[^ ^30^ ^]^ and Fe_3_O_4_ phases (JCPDS No. 01‐077‐1545), with distinct diffraction peaks for both. Importantly, no Ru or RuO_2_ peaks are detected, indicating that the phosphidation process does not impact the Ru SAs, affirming the formation of Ru_SAs_/Ni_2_P@Fe_3_O_4_.^[^ ^31^ ^]^ The SEM results in Figure 2d reveal that Ru_SAs_/Ni_2_P@Fe_3_O_4_ maintains its core‐shell structure, though slight surface deformation occurred, likely attributed to gas release during calcination. Simultaneously, the findings from transmission electron microscopy (TEM) further validate this observation (Figure 2f). The aberration‐corrected high‐angle annular dark‐field scanning transmission electron microscopy (AC HAADF‐STEM) (Figure 2g,h) images indicate that the angle between the ($\bar{1}$11) and (111) crystal planes of the hexagonal Ni_2_P are 69° (the bright spots in the images correspond to dispersed Ru SAs). As control samples, Ni_2_P@Fe_3_O_4_ and Ru_SAs_/Ni_2_P nanomaterials were synthesized (Figures S6a and S7a, Supporting Information). SEM and TEM images show the core‐shell structure of Ni_2_P@Fe_3_O_4_, with Fe_3_O_4_ as the core and Ni_2_P shell (Figure S6b–l, Supporting Information). Additionally, Ru SAs are observed on the Ni_2_P nanosheets in the Ru_SAs_/Ni_2_P sample (Figure S7b–h, Supporting Information). The selected area electron diffraction (SAED) pattern (Figure 2i) reveals two materials in Ru_SAs_/Ni_2_P@Fe_3_O_4_, with bright pink and cyan rings indicating Ni_2_P and Fe_3_O_4_ phases, respectively. Structural characterization by high‐angle annular dark‐field scanning transmission (HAADF‐STEM) (Figure 2j) reveals a well‐defined core‐shell architecture, with Fe and O atoms constituting the core region and Ni and P atoms demonstrating a homogeneous distribution across the nanosheet. This atomic arrangement suggests successful substitution of Ni sites by Ru SAs, which is fully consistent with our preceding XRD analyses.

**Figure 3:**
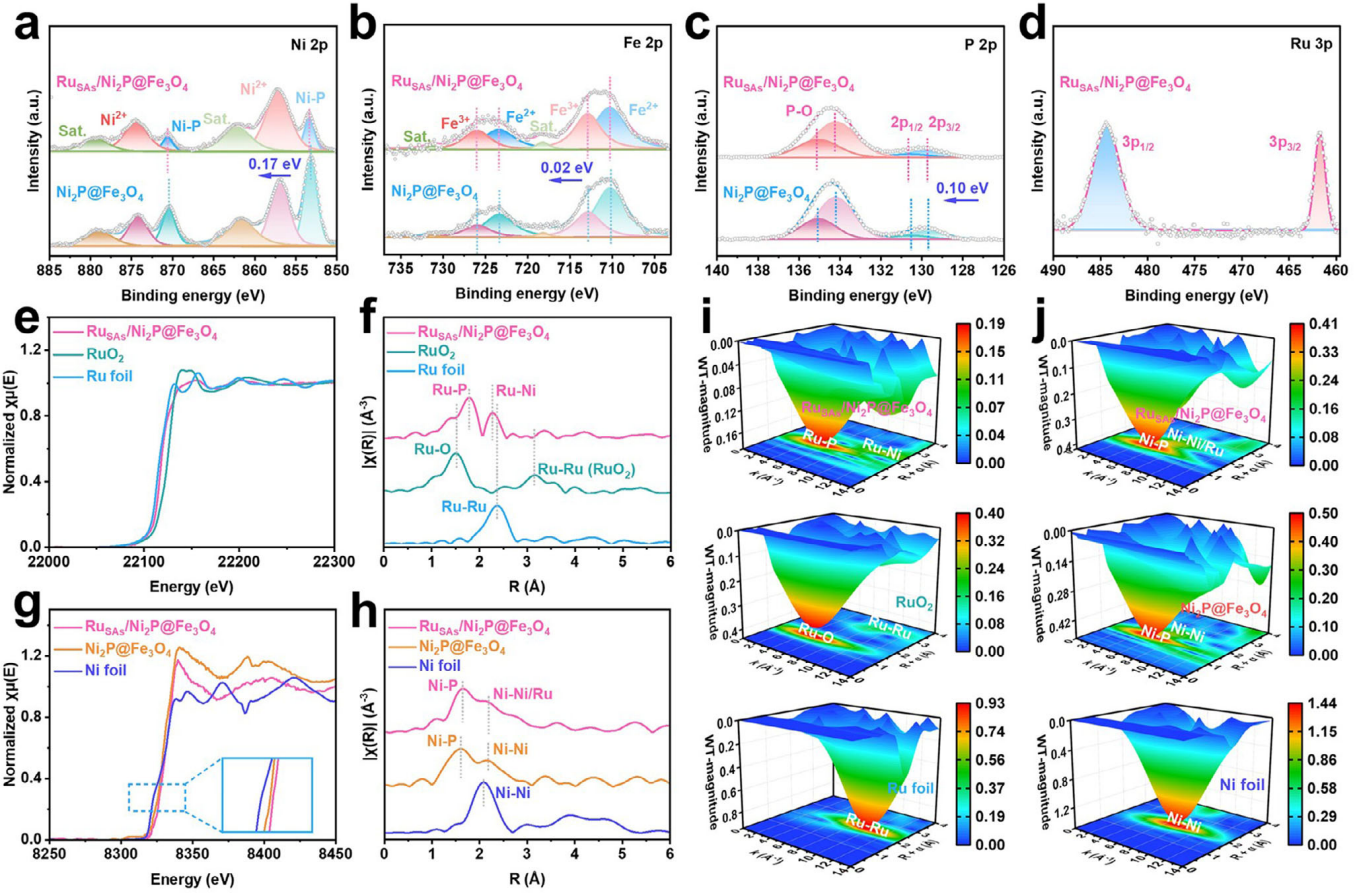
Phase and surface chemical characteristics analyses of Ru_SAs_/Ni_2_P@Fe_3_O_4_. High‐resolution XPS spectra of a) Ni 2p, b) Fe 2p, c) P 2p, and d) Ru 3p of Ru_SAs_/Ni_2_P@Fe_3_O_4_ and Ni_2_P@Fe_3_O_4_, respectively. e) Ru K‐edge XANES and f) EXAFS spectra of Ru_SAs_/Ni_2_P@Fe_3_O_4_, RuO_2_, and Ru foil. g) Ni K‐edge XANES and h) EXAFS spectra of Ru_SAs_/Ni_2_P@Fe_3_O_4_, Ni_2_P@Fe_3_O_4_, and Ni foil. i) WT‐EXAFS of Ru_SAs_/Ni_2_P@Fe_3_O_4_, RuO_2_, and Ru foil. j) WT‐EXAFS of Ru_SAs_/Ni_2_P@Fe_3_O_4_, Ni_2_P@Fe_3_O_4_, and Ni foil.

X‐ray photoelectron spectroscopy (XPS) was utilized to investigate the chemical and electronic states of the material after doping with Ru SAs.^[^ ^32^ ^]^ The XPS results for Ru_SAs_/Ni_2_P@Fe_3_O_4_ and Ni_2_P@Fe_3_O_4_ are presented in **Figure** **3**a–d and Figure S8 (Supporting Information). In Ru_SAs_/Ni_2_P@Fe_3_O_4_, the binding energies for Ni‐P are 853.39 eV (Ni 2p_3/2_) and 870.64 eV (Ni 2p_1/2_), which are higher than the corresponding Ni 2p values (853.22 eV (Ni 2p_3/2_) and 870.47 eV (Ni 2p_1/2_)) in Ni_2_P@Fe_3_O_4_.^[^ ^33^ ^]^ This elevation is attributed to the doping of highly electronegative Ru SAs, leading to enhanced electron affinity of Ru compared to Ni, thus facilitating electron transfer from Ni to Ru. The XPS analysis of Fe 2p shows negligible changes in binding energy after Ru SAs doping (Figure 3b), indicating that Ru SAs are located on the Ni_2_P nanosheet surface and have almost no impact on the Fe_3_O_4_ core. The P 2p spectrum (Figure 3c) shows a P─O bond at 133.44 eV in both samples, primarily due to air oxidation. The binding energies for P 2p_3/2_ in Ru_SAs_/Ni_2_P@Fe_3_O_4_ and Ni_2_P@Fe_3_O_4_ are 129.72 and 129.62 eV, respectively, while for P 2p_1/2_ they are 130.64 and 130.54 eV. The higher P 2p binding energy in Ru_SAs_/Ni_2_P@Fe_3_O_4_ indicates Ru‐P bond formation during synthesis. The strong Ru‐P and Ru‐Ni interactions in this core‐shell structure reduce the electron density at the P site, altering the charge distribution and facilitating electron transfer to Ru SAs.^[^ ^34^ ^]^ This electron‐rich state of Ru may enhance the adsorption of H, thereby improving the electrocatalytic activity. Furthermore, the Ru 3p spectrum in Figure 3d reveals binding energies of 484.36 eV for Ru 3p_1/2_ and 461.73 eV for Ru 3p_3/2_, with no RuO_2_ peaks being detected, confirming that Ru exists as SAs.

To further explore Ru SAs, the local electronic environment and coordination in Ru_SAs_/Ni_2_P@Fe_3_O_4_ were assessed by X‐ray absorption near‐edge structure (XANES) and extended X‐ray absorption fine structure (EXAFS) measurements.^[^ ^35^, ^36^ ^]^ Figure 3e displays the Ru K‐edge XANES spectra of Ru_SAs_/Ni_2_P@Fe_3_O_4_, RuO_2_, and Ru foil. The Ru K‐edge XANES spectrum indicates that the energy value shifts positively with increasing Ru oxidation state in Ru_SAs_/Ni_2_P@Fe_3_O_4_, confirming the presence of the Ru SAs in Ru_SAs_/Ni_2_P@Fe_3_O_4_. These Ru SAs can bond with Ni and P in Ru_SAs_/Ni_2_P@Fe_3_O_4_ to form Ru‐Ni and Ru‐P bonds. As shown in Figure 3f, the Fourier‐transformed EXAFS spectrum of Ru_SAs_/Ni_2_P@Fe_3_O_4_ exhibits two peaks at ≈1.82 and 2.31 Å, which are attributed to Ru‐P and Ru‐Ni bonds, respectively.^[^ ^37^ ^]^ The lack of a Ru‐Ru bond near 2.37 Å suggests atomic dispersion of Ru in Ru_SAs_/Ni_2_P@Fe_3_O_4_.^[^ ^38^ ^]^ The Wavelet Transform‐EXAFS (WT‐EXAFS) analysis illustrates Ru SAs dispersion in Ru_SAs_/Ni_2_P@Fe_3_O_4_, RuO_2_, and Ru foil (Figure 3i). Specifically, the corresponding fitting parameters for Ru_SAs_/Ni_2_P@Fe_3_O_4_ and Ru foil are shown in Figures S9 and S10, Table S2 (Supporting Information). The WT‐EXAFS spectrum of Ru_SAs_/Ni_2_P@Fe_3_O_4_ shows a pronounced signal at 6 Å^−1^, and no signals corresponding to Ru‐Ru bonds in RuO_2_ or Ru foil are observed.^[^ ^39^ ^]^ This indicates that Ru SAs are uniformly dispersed within Ru_SAs_/Ni_2_P@Fe_3_O_4_. The Ni K‐edge XANES spectra of Ru_SAs_/Ni_2_P@Fe_3_O_4_ and Ni_2_P@Fe_3_O_4_ are similar, but distinct from Ni foil (Figure 3g), suggesting structural integrity after Ru SAs doping, consistent with XRD results. The higher energy shift of the Ni K‐edge suggests electron transfer from Ni to Ru. EXAFS spectra indicate that Ni‐Ni and Ni‐P bonds in Ru_SAs_/Ni_2_P@Fe_3_O_4_ and Ni_2_P@Fe_3_O_4_ are similar, confirming structural stability (Figure 3h). Notably, the Ni‐Ni bond peak in Ru_SAs_/Ni_2_P@Fe_3_O_4_ is slightly larger, which is caused by the formation of partial Ni‐Ru bonds due to the incorporation of Ru. These observations are supported by the WT‐EXAFS pattern (Figure 3j). Furthermore, the corresponding fitting parameters for Ru_SAs_/Ni_2_P@Fe_3_O_4_, Ni_2_P@Fe_3_O_4,_ and Ni foil are shown in Figures S11 and S12, Table S3 (Supporting Information). To investigate the effect of Ru SAs doping on the chemical state of P, P L‐edge XANES characterizations were conducted on both Ru_SAs_/Ni_2_P@Fe_3_O_4_ and the control sample Ni_2_P@Fe_3_O_4_. As shown in Figure S13, the P L_3_‐edge main peak of Ru_SAs_/Ni_2_P@Fe_3_O_4_ shifts to a higher energy region compared to that of Ni_2_P@Fe_3_O_4_. This energy shift aligns with the increased P 2p binding energy observed in XPS measurements, consistently demonstrating that the introduction of Ru SAs reduces the electron cloud density of P atoms. These results directly confirm the formation of Ru‐P interfacial coordination interactions in the catalyst.^[^ ^40^, ^41^ ^]^ Overall, Ru SAs doping induces local electronic and structural perturbations.

### 2.2 Magnetic Field‐Enhanced HER

**Figure 4:**
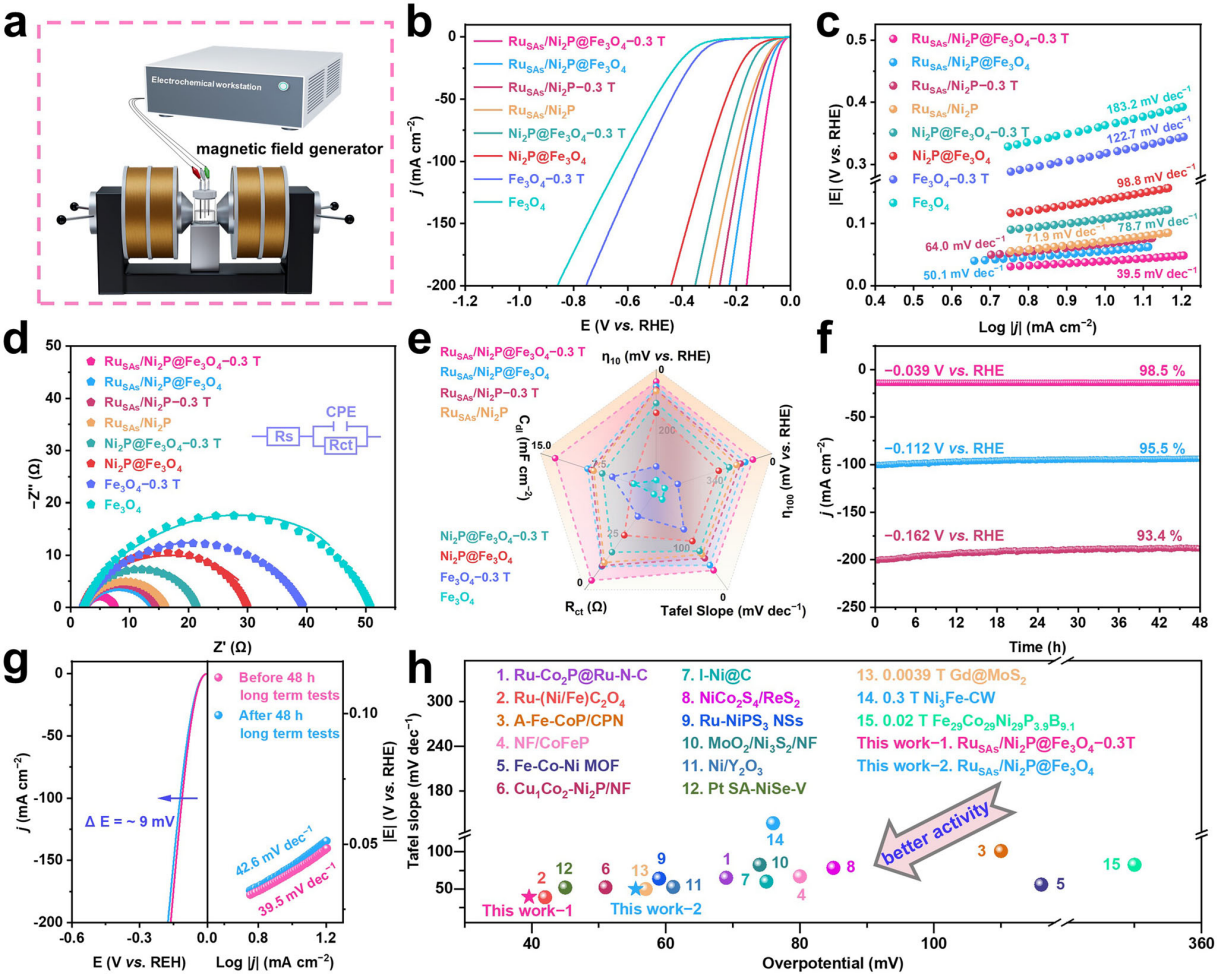
Performance investigations of alkaline HER on Ru_SAs_/Ni_2_P@Fe_3_O_4_ under the action of an external magnetic field. −0.3 T means the application of an external magnetic field with an intensity of 0.3 T to the sample during the performance testing process. a) Electrochemistry schematic of the applied magnetic field. b) LSV curves, c) Tafel slopes, d) EIS spectrums with specific R_ct_ values inset, and e) radar map depicting Ru_SAs_/Ni_2_P@Fe_3_O_4_ and its control samples under the conditions with and without a 0.3 T external magnetic field, in 1 M KOH electrolyte. f) I‐t curves of Ru_SAs_/Ni_2_P@Fe_3_O_4_−0.3 T at different potentials within 48 h (tested under continuous application of 0.3 T external magnetic field). g) LSV curves and Tafel slopes of Ru_SAs_/Ni_2_P@Fe_3_O_4_−0.3 T before and after 48 h long‐term test under a 0.3 T magnetic field. h) Comparison of the performance between Ru_SAs_/Ni_2_P@Fe_3_O_4_ and Ru_SAs_/Ni_2_P@Fe_3_O_4_−0.3 T with that of the reported Ru SAs, Ni‐based, and Fe‐based HER catalysts, as well as with other catalysts under the action of an external magnetic field (Detailed information can be found in the Supporting Information).

To investigate the impact of Ru_SAs_/Ni_2_P@Fe_3_O_4_ on HER electrocatalytic activity in an external magnetic field, electrochemical experiments were performed using a three‐electrode system in 1 M KOH solution.^[^ ^9^, ^42^, ^43^ ^]^ Performance comparisons among Ru_SAs_/Ni_2_P, Ni_2_P@Fe_3_O_4_, and Fe_3_O_4_ were conducted. **Figure** **4**a and Figure S14 illustrate the schematic of the electrocatalytic setup under magnetic field influence. The linear sweep voltammetry (LSV) results in Figure 4b indicate that the Ru_SAs_/Ni_2_P@Fe_3_O_4_ catalyst achieves a current density of 10 mA cm^−2^ at 55.5 mV, surpassing Ru_SAs_/Ni_2_P (71.6 mV), Ni_2_P@Fe_3_O_4_ (143.4 mV), and Fe_3_O_4_ (364.3 mV). This suggests improved HER activity due to interface formation and Ru SAs doping.^[^ ^35^ ^]^ To explore the effect of the external magnetic field, a magnetic field of 0.3 T was applied. It should be particularly noted that −0.3 T indicates the application of an external magnetic field of 0.3 T to the sample. The overpotentials for Ru_SAs_/Ni_2_P@Fe_3_O_4_−0.3 T, Ru_SAs_/Ni_2_P−0.3 T, Ni_2_P@Fe_3_O_4_−0.3 T, and Fe_3_O_4_−0.3 T are 38.9, 67.4, 111.3, and 318.6 mV, respectively, indicating a substantial enhancement in catalytic performance for Ru_SAs_/Ni_2_P@Fe_3_O_4_ and Ni_2_P@Fe_3_O_4_, while Ru_SAs_/Ni_2_P showed minimal change.

Existing studies have shown that mass transport in electrochemical processes can be affected by magnetic fields. However, in this study, multiple pieces of evidence indicate that the mass transport effect is not the cause of enhanced HER activity under a magnetic field. First, I‐t tests were conducted on Ru_SAs_/Ni_2_P@Fe_3_O_4_, Ru_SAs_/Ni_2_P, Ni_2_P@Fe_3_O_4_, and Fe_3_O_4_ under different magnetic field conditions, respectively. The results show that with the increase of magnetic field strength, the current densities of all ferromagnetic materials containing Fe_3_O_4_ (Ru_SAs_/Ni_2_P@Fe_3_O_4_, Ni_2_P@Fe_3_O_4_, Fe_3_O_4_) increase significantly at the same voltage. In contrast, the current density of the paramagnetic material Ru_SAs_/Ni_2_P remains almost unchanged as magnetic field strength increases (Figure S15, Supporting Information). This indicates that the mass transport effect under an external magnetic field should make a minimal contribution to the enhancement of HER activity of ferromagnetic catalysts. Second, according to the LSV data, the overpotentials of Ru_SAs_/Ni_2_P@Fe_3_O_4_, Ni_2_P@Fe_3_O_4_, and Fe_3_O_4_ at a current density of 10 mA cm^−2^ decrease by 16.6, 32.1, and 45.7 mV, respectively, with and without the application of a 0.3 T magnetic field. Moreover, the overpotential of paramagnetic Ru_SAs_/Ni_2_P shows no significant change under the magnetic field (Ru_SAs_/Ni_2_P−0.3 T: 67.4 mV, Ru_SAs_/Ni_2_P: 71.6 mV). All these indicate that the mass transfer effect induced by the external magnetic field should make a minimal contribution to the improvement of HER activity of ferromagnetic catalysts, which is consistent with the results reported by Ren et al.^[^ ^19^ ^]^ Furthermore, according to previous literature reports and the Grotthuss mechanism theory,^[^ ^19^ ^]^ protons migrate in aqueous solutions through relay transfer via hydrogen bond networks, rather than relying on long‐distance physical diffusion. Thus, the impact of mass transport effects on the reaction is inherently relatively weak. After excluding the influence of mass transport, this overpotential difference indicates the importance of the Fe_3_O_4_ cores in Ru_SAs_/Ni_2_P@Fe_3_O_4_ and Ni_2_P@Fe_3_O_4_ under magnetic influence, likely because the magnetic field enhances their electrocatalytic performance by regulating the spin states of Fe atoms and the overall electronic structure of the material. In alkaline media, HER generally involves the Volmer, Heyrovsky, and Tafel mechanisms, with Tafel slopes of 120, 40, and 30 mV dec^−1^, respectively.^[^ ^44^, ^45^, ^46^ ^]^ The Tafel slope for Ru_SAs_/Ni_2_P@Fe_3_O_4_ is ≈50.1 mV dec^−1^ (Figure 4c), better than that of Ru_SAs_/Ni_2_P (71.9 mV dec^−1^), Ni_2_P@Fe_3_O_4_ (98.8 mV dec^−1^), and Fe_3_O_4_ (183.2 mV dec^−1^), indicating a Volmer‐Heyrovsky mechanism where the Heyrovsky step is rate‐determining. Thus, the combination of adsorbed hydrogen and protons, as well as the transfer of electrons, jointly govern the reaction rate, highlighting the advantage of electron regulation via interface and SAs interactions in enhancing reaction kinetics.^[^ ^47^, ^48^ ^]^ Furthermore, the Tafel slopes for Ru_SAs_/Ni_2_P@Fe_3_O_4_−0.3 T, Ru_SAs_/Ni_2_P−0.3 T, Ni_2_P@Fe_3_O_4_−0.3 T, and Fe_3_O_4_−0.3 T are 39.5, 64.0, 78.7, and 122.7 mV dec^−1^, respectively. Compared with the test results of samples without a magnetic field, the reaction kinetics of Ru_SAs_/Ni_2_P@Fe_3_O_4_ and Ni_2_P@Fe_3_O_4_ under a 0.3 T magnetic field are enhanced, indicating that the magnetic field improved the catalytic performance.

To evaluate the electrochemically active surface area (ECSA) of the catalyst, cyclic voltammetry (CV) curves at different scan rates (10–80 mV s^−1^) were recorded in the non‐Faradaic potential range (Figure S16, Supporting Information). The double‐layer capacitance (C_dl_) was calculated through the linear slope of the capacitance current and the scanning rate (Figure S17a, Supporting Information), and the LSV was further normalized by ECSA (*j*_ECSA_). The results (Figure S17b,c, Supporting Information) show that at the same overpotential, Ru_SAs_/Ni_2_P@Fe_3_O_4_−0.3 T exhibits a significant increase in *j*_ECSA_. This outcome suggests that a magnetic field can activate more atoms as catalytically active sites.^[^ ^49^ ^]^ This may be attributed to the synergistic effects from the core‐shell interface engineering of Ru_SAs_/Ni_2_P and Fe_3_O_4_, along with the influence of an external magnetic field. The core‐shell structure offers a greater specific surface area, generates more active sites,^[^ ^50^ ^]^ and provides better hydrogen adsorption free energy. Additionally, the magnetic field may modify the electron spin state of the Fe_3_O_4_ core, enhancing interfacial charge transfer with Ru_SAs_/Ni_2_P, thereby accelerating the HER reaction. Electrochemical impedance spectroscopy (EIS) results indicate that the charge transfer resistance (R_ct_) decreases under an external magnetic field, supporting the idea that the magnetic field boosts electron transfer (Figure 4d; Figure S18, Supporting Information). To elucidate the HER activity sources for each catalyst, parameters such as ɳ_10_ (overpotential at a current density of 10 mA cm^−2^) (Figure S19a, Supporting Information), ɳ_100_ (overpotential at a current density of 100 mA cm^−2^) (Figure S19b, Supporting Information), Tafel slope, R_ct_, and C_dl_ were compiled and analysed in a radar chart (Figure 4e). It clearly shows the superior performance of Ru_SAs_/Ni_2_P@Fe_3_O_4_−0.3 T across multiple metrics.^[^ ^51^ ^]^ Durability tests via chronoamperometry demonstrate that Ru_SAs_/Ni_2_P@Fe_3_O_4_−0.3 T, after 48 h of testing at different potentials, exhibits a current retention rate of over 93.4% at each potential (Figure 4f). Furthermore, after 48 h of continuous operation at a current density of 100 mA cm^−2^, the overpotential increases by only 9 mV (Figure 4g). These results collectively confirm that the Ru_SAs_/Ni_2_P@Fe_3_O_4_ electrode possesses excellent long‐term stability. Overall, this catalyst outperforms advanced Ru SAs and nickel‐based and iron‐based catalysts in alkaline electrolytes, especially under an external magnetic field (Figure 4h; Table S4, Supporting Information). Furthermore, the SEM, XRD, and Raman spectroscopy analyses presented in Figures S20 and S21 (Supporting Information) demonstrate that the structure and composition of Ru_SAs_/Ni_2_P@Fe_3_O_4_−0.3 T remain intact after stability tests, with no observable alterations in morphology or chemical composition even under magnetic field conditions. To directly observe the Ru SAs under the magnetic field, AC‐HAADF‐STEM characterization was performed on Ru_SAs_/Ni_2_P@Fe_3_O_4_−0.3 T (Figure S22, Supporting Information). The results clearly show that after the 48‐h stability test under a 0.3 T magnetic field, Ru SAs are still uniformly dispersed on Ni_2_P nanosheets, and no obvious aggregation or migration of Ru atoms is observed. These characterization results from the macroscopic scale (SEM, XRD, Raman) to the atomic scale (AC‐HAADF‐STEM) collectively confirm that the Ru_SAs_/Ni_2_P@Fe_3_O_4_−0.3 T electrocatalyst exhibits excellent structural stability under the magnetic field. To investigate the influence of varying magnetic field intensities on the HER performance of Ru_SAs_/Ni_2_P@Fe_3_O_4_, LSV measurements were conducted under magnetic fields of 0, 0.1, 0.2, 0.3, 0.4, and 0.5 T. As shown in Figure S23 (Supporting Information), the overpotential of the catalyst exhibits a gradient decrease with increasing magnetic field strength. The Tafel slopes of the catalyst show a significant reduction as the magnetic field intensity increases. These results indicate that increasing the magnetic field strength effectively optimizes the HER performance of Ru_SAs_/Ni_2_P@Fe_3_O_4_, significantly accelerating the reaction kinetics and thereby enabling the catalyst to drive the HER more efficiently at lower energy consumption.^[^ ^52^ ^]^ However, when increasing the magnetic field to 0.4 and 0.5 T, the overpotential and Tafel slope stabilize without significant changes. This confirms that 0.3 T has reached the magnetic field regulation saturation threshold.

In conclusion, the doping of Ru SAs modulates the electronic structure and may optimize the free energy of H adsorption, thereby promoting the HER reaction. The core‐shell interface of Ru_SAs_/Ni_2_P and Fe_3_O_4_ enhances the specific surface area, generating more active sites for the interaction between H_ads_ and protons, thereby facilitating the electron transfer and accelerating the reaction rate. In addition, the Fe_3_O_4_ core exhibits unique magnetic properties. Under the influence of an external magnetic field, its electron distribution and spin state may be altered, enhancing the interfacial charge transfer with Ru_SAs_/Ni_2_P and further promoting the HER reaction.

### 2.3 Magnetic Field‐Driven Spintronic Effect Mechanism

To verify the influence of magnetic fields on spin state transitions and electron distribution, and thereby clarify the intrinsic mechanism underlying the enhancement of HER performance, various characterization tests were conducted. Hysteresis loop results indicate that both Ru_SAs_/Ni_2_P@Fe_3_O_4_ and Fe_3_O_4_ exhibit ferromagnetic characteristics, while Ru_SAs_/Ni_2_P shows paramagnetic behavior (Figure S24, Supporting Information), suggesting that the Fe_3_O_4_ substrate is the primary source of ferromagnetism in the Ru_SAs_/Ni_2_P@Fe_3_O_4_ sample.^[^ ^53^ ^]^ Additionally, DFT calculations reveal that the d‐orbitals of Fe atoms in Fe_3_O_4_ are divided into d_xz_, d_yz_, d_xy_, d_z2_, and d_x2−y2_ (Figure S25, Supporting Information).^[^ ^54^ ^]^ Among them, d_xz_, d_yz_, and d_xy_ belong to the low‐energy t_2g_ orbitals, while d_z2_ and d_x2−y2_ belong to the high‐energy e_g_ orbitals. When both t_2g_ and e_g_ orbitals contain unpaired electrons (e.g., t_2g_^3^e_g_^2^ configuration), a HS state is formed with a total of 5 unpaired electrons. When electrons occupy only the t_2g_ orbitals and are paired (e.g., t_2g_^5^e_g_^0^ configuration), a LS state is formed with the number of unpaired electrons reduced to 1. Based on the above theoretical analysis of the spin states of Fe atoms, XANES, Mössbauer spectra, electron paramagnetic resonance (EPR), and superconducting quantum interference device (SQUID) measurements were used to systematically analyze how magnetic fields regulate the spin states of Fe atoms in ferromagnetic Fe_3_O_4_ supports.

**Figure 5:**
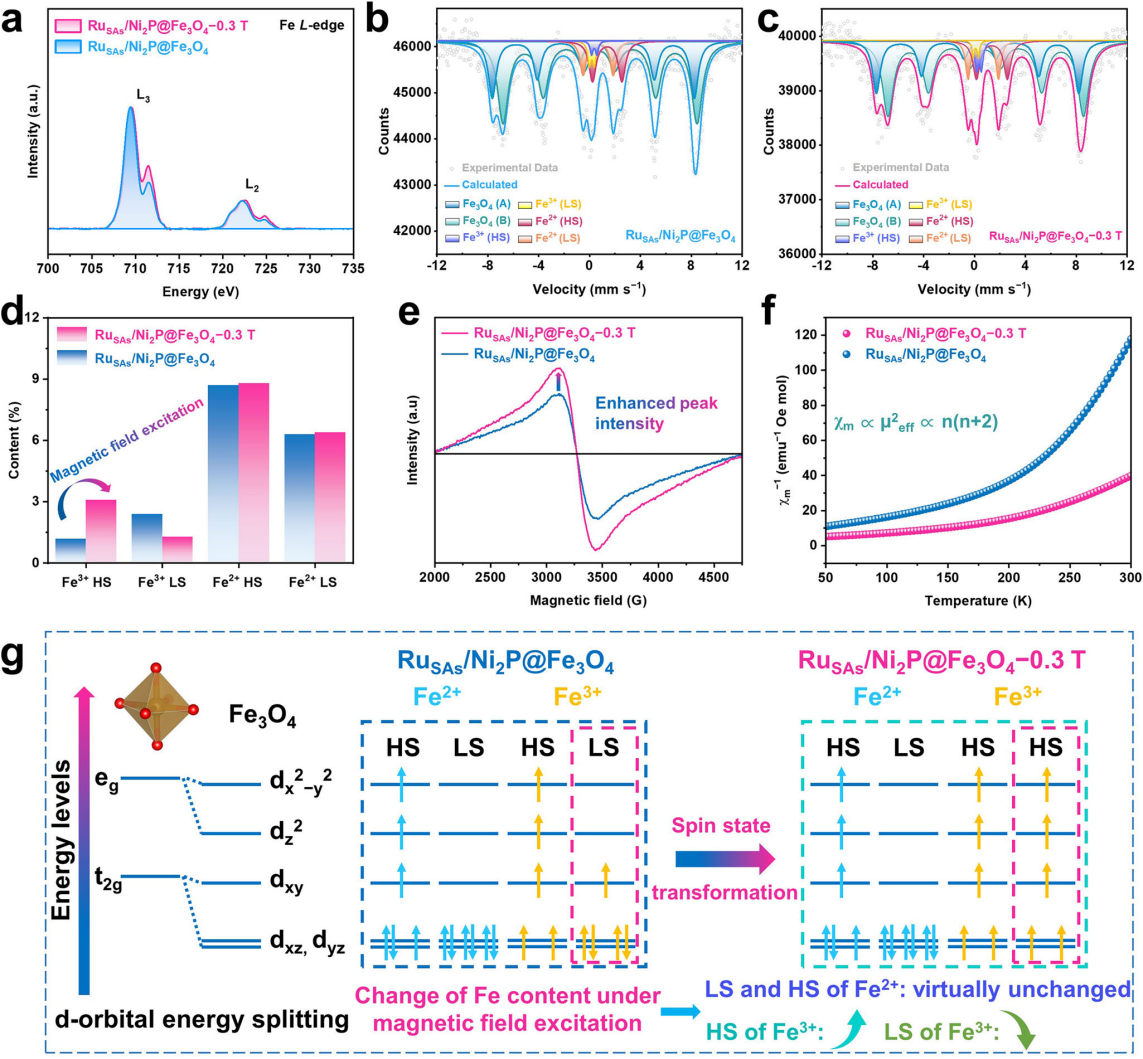
Electronic structure characterization of magnetic field‐driven spin state transition in Ru_SAs_/Ni_2_P@Fe_3_O_4_−0.3 T indicates that the sample has been treated under a 0.3 T magnetic field. a) Fe L‐edge XANES of Ru_SAs_/Ni_2_P@Fe_3_O_4_ and Ru_SAs_/Ni_2_P@Fe_3_O_4_−0.3 T. Mössbauer spectra of b) Ru_SAs_/Ni_2_P@Fe_3_O_4_ and c) Ru_SAs_/Ni_2_P@Fe_3_O_4_−0.3 T. d) Content percentage of different spin states of Fe in Ru_SAs_/Ni_2_P@Fe_3_O_4_ and Ru_SAs_/Ni_2_P@Fe_3_O_4_−0.3 T. e) EPR spectra, and f) ZFC curves related to magnetic susceptibility of Ru_SAs_/Ni_2_P@Fe_3_O_4_ and Ru_SAs_/Ni_2_P@Fe_3_O_4_−0.3 T. g) Schematic diagram of the spin state transformation of Fe^2+^ and Fe^3+^ in Fe_3_O_4_ of Ru_SAs_/Ni_2_P@Fe_3_O_4_−0.3 T electrocatalyst driven by the enhancement strategy excited by an external magnetic field.

Fe L‐edge XANES spectra (**Figure** **5**a) show that both Ru_SAs_/Ni_2_P@Fe_3_O_4_−0.3 T and Ru_SAs_/Ni_2_P@Fe_3_O_4_ exhibit characteristic L_3_ and L_2_ edge peaks corresponding to Fe 2p→3d orbital transitions, whose intensity is positively correlated with the density of unpaired electrons in the 3d orbitals.^[^ ^55^, ^56^ ^]^ Notably, the I(L_3_)/[I(L_3_) + I(L_2_)] branching ratio of Ru_SAs_/Ni_2_P@Fe_3_O_4_−0.3 T (0.778) is higher than that of Ru_SAs_/Ni_2_P@Fe_3_O_4_ (0.774) (Table S5, Supporting Information). This indicates that Ru_SAs_/Ni_2_P@Fe_3_O_4_−0.3 T has more half‐filled 3d orbitals and unpaired electrons, driving the spin state transition from the LS t_2g_ state to the HS e_g_ state.^[^ ^57^ ^]^ This transition is directly confirmed by the significant increase in the intensity of the Fe^3+^ characteristic peak at 711.5 eV, while the Fe^2+^ peak intensity at 709.5 eV remains essentially unchanged, verifying the regulatory effect of the magnetic field on the Fe^3+^ spin state. To quantify the spin state distribution, Mössbauer spectra were obtained for Ru_SAs_/Ni_2_P@Fe_3_O_4_ and Ru_SAs_/Ni_2_P@Fe_3_O_4_−0.3 T. According to isomer shift (IS) and quadrupole splitting (QS) values (Tables S6 and S7, Supporting Information), two sextets and four doublets were identified in both samples, corresponding to Fe_3_O_4_ (A), Fe_3_O_4_ (B), Fe^2+^ (HS), Fe^2+^ (LS), Fe^3+^ (HS), and Fe^3+^ (LS), respectively. Comparison of the Mössbauer spectra of Ru_SAs_/Ni_2_P@Fe_3_O_4_ and Ru_SAs_/Ni_2_P@Fe_3_O_4_−0.3 T shows that the sextets for Fe_3_O_4_ remain stable in positions and proportions (Figure 5b,c), indicating that the crystal structure and magnetic properties of the Fe_3_O_4_ phase exhibit high stability under an external magnetic field.^[^ ^58^ ^]^ The magnetic field has a significant influence on the content proportion of HS and LS states of Fe^3+^. Under the external magnetic field (Figure 5c, Ru_SAs_/Ni_2_P@Fe_3_O_4_−0.3 T), the intensity of the characteristic absorption peak corresponding to the HS state of Fe^3+^ is significantly enhanced, while the peak intensity of the LS state of Fe^3+^ decreases accordingly, confirming the conclusion that the spin state of Fe^3+^ in the XANES spectrum changes from LS to HS.^[^ ^59^ ^]^ Notably, the spin state of Fe^2+^ remained almost unchanged, a stability that is quantitatively supported by its distinct Mössbauer parameters. Specifically, the quadrupole splitting (QS) value for Fe^2+^ (≈2.4 mm s^−1^) is significantly larger than that for Fe^3+^ (≈0.4 mm s^−1^) in the system (Tables S6 and S7, Supporting Information). Such a large QS value provides direct evidence that the electron cloud around Fe^2+^ is subject to limited delocalization. This localization enhances the ligand field, leading to a higher 3d orbital splitting energy, which stabilizes its electron configuration.^[^ ^60^ ^]^ Consequently, the spin state of Fe^2+^ is insensitive to the energy perturbation from the external magnetic field. This conclusion is further corroborated by the quantitative peak fitting of the spectra (Figure 5d). The data show that in Ru_SAs_/Ni_2_P@Fe_3_O_4_ without a magnetic field, the HS state of Fe^3+^ accounts for 1.2% and the LS state of Fe^3+^ accounts for 2.4%. In Ru_SAs_/Ni_2_P@Fe_3_O_4_−0.3 T with an applied magnetic field, the HS state of Fe^3+^ increased to 3.1%, while the LS state of Fe^3+^ decreased to 1.3%. In stark contrast, the proportion of the HS state of Fe^2+^ only changes from 8.7% to 8.8%, and the LS state from 6.3% to 6.4%, confirming its stability. This difference indicates that the magnetic field mainly regulates the 3d orbital electron occupation state of Fe^3+^, promoting its transition from the LS state to the HS state of Fe^3+^, and thereby changing the overall spintronic structure of the material.

In addition, the combined analysis of EPR spectra and SQUID magnetic characterization provides a complete chain of evidence for the spin state transformation of Fe^3+^ induced by magnetic fields, ranging from microscopic electronic structure to macroscopic magnetic correspondence. In the EPR spectrum (Figure 5e), Ru_SAs_/Ni_2_P@Fe_3_O_4_−0.3 T shows a stronger signal near the g value compared with Ru_SAs_/Ni_2_P@Fe_3_O_4_, which directly reflects that the magnetic field induces the ferromagnetic material to generate more unpaired electrons, indicating that the magnetic field alters the electronic structure of the material.^[^ ^59^ ^]^ To further analyze the electronic spin configuration of Fe, zero‐field cooling (ZFC) variable‐temperature magnetization measurements are performed. The results in Figure 5f show that the magnetic susceptibility (χ_m_) of Ru_SAs_/Ni_2_P@Fe_3_O_4_−0.3 T is significantly higher than that of Ru_SAs_/Ni_2_P@Fe_3_O_4_. According to the Curie‐Weiss law (χ_m_ = C/(T‐θ), where C is the Curie constant and θ is the Curie temperature) and the relationship between the effective magnetic moment µ_eff_ = (2C/N_A_)^1/2^, combined with the relationship between the spin state and the number of unpaired electrons (n) (µ_eff_ = (n(n+2))^1/2^), a linear relationship exists among χ_m_, µ_eff_^2^, and n(n+2).^[^ ^61^, ^62^ ^]^ Therefore, the higher χ_m_ value of Ru_SAs_/Ni_2_P@Fe_3_O_4_−0.3 T indicates a greater number of unpaired electrons, confirming that Fe^3+^ in the ferromagnetic material undergoes an LS‐to‐HS transition.

Through multi‐dimensional characterization, this study reveals the differential regulation of magnetic fields on the spin states of Fe ions in the catalyst. Under excitation by a magnetic field, the d^5^ electrons of Fe^3+^ transition from the LS state (fully occupied t_2g_ orbitals, empty e_g_ orbitals) to the HS state (3 electrons in t_2g_ orbitals, 2 electrons in e_g_ orbitals), while the spin states of Fe^2+^ remain unchanged regardless of being in the LS state (t_2g_^6^e_g_^0^) or HS state (t_2g_^4^e_g_^2^). Therefore, under the influence of a magnetic field, the content of Fe^3+^ in the HS state increases, while its LS state content decreases, whereas the contents of Fe^2+^ in both HS and LS states remain almost unchanged (Figure 5g). During this process, the e_g_ orbitals of Fe^3+^ transition from “empty” to “half‐filled”, increasing the number of unpaired electrons. This promotes more e_g_ orbital electrons to participate in reactant adsorption and charge transfer, enhancing electron transport efficiency and thereby accelerating catalytic reaction kinetics, providing a microscopic mechanism for the improvement of catalytic performance by magnetic fields.

### 2.4 Theoretical Calculations

Based on synchrotron radiation and XPS characterization of Ru_SAs_/Ni_2_P@Fe_3_O_4_ and Ni_2_P@Fe_3_O_4_, it was found that Ru SAs doping induces local electron redistribution. Further combined with XANES, Mössbauer spectroscopy, EPR, and SQUID measurements on Ru_SAs_/Ni_2_P@Fe_3_O_4_ with and without a magnetic field, it is demonstrated that the magnetic field facilitates the excitation of Fe (3d t_2g_) electrons from the LS state to the HS state (3d e_g_). This spin‐state transition triggers the rearrangement of electron distribution in the d orbitals, generating more unpaired electrons in the 3d orbitals.^[^ ^63^, ^64^ ^]^ According to these experimental findings, DFT calculations were systematically performed to explore the synergistic mechanism of external magnetic field‐driven spin‐state transitions and SAs doping on HER performance.

**Figure 6:**
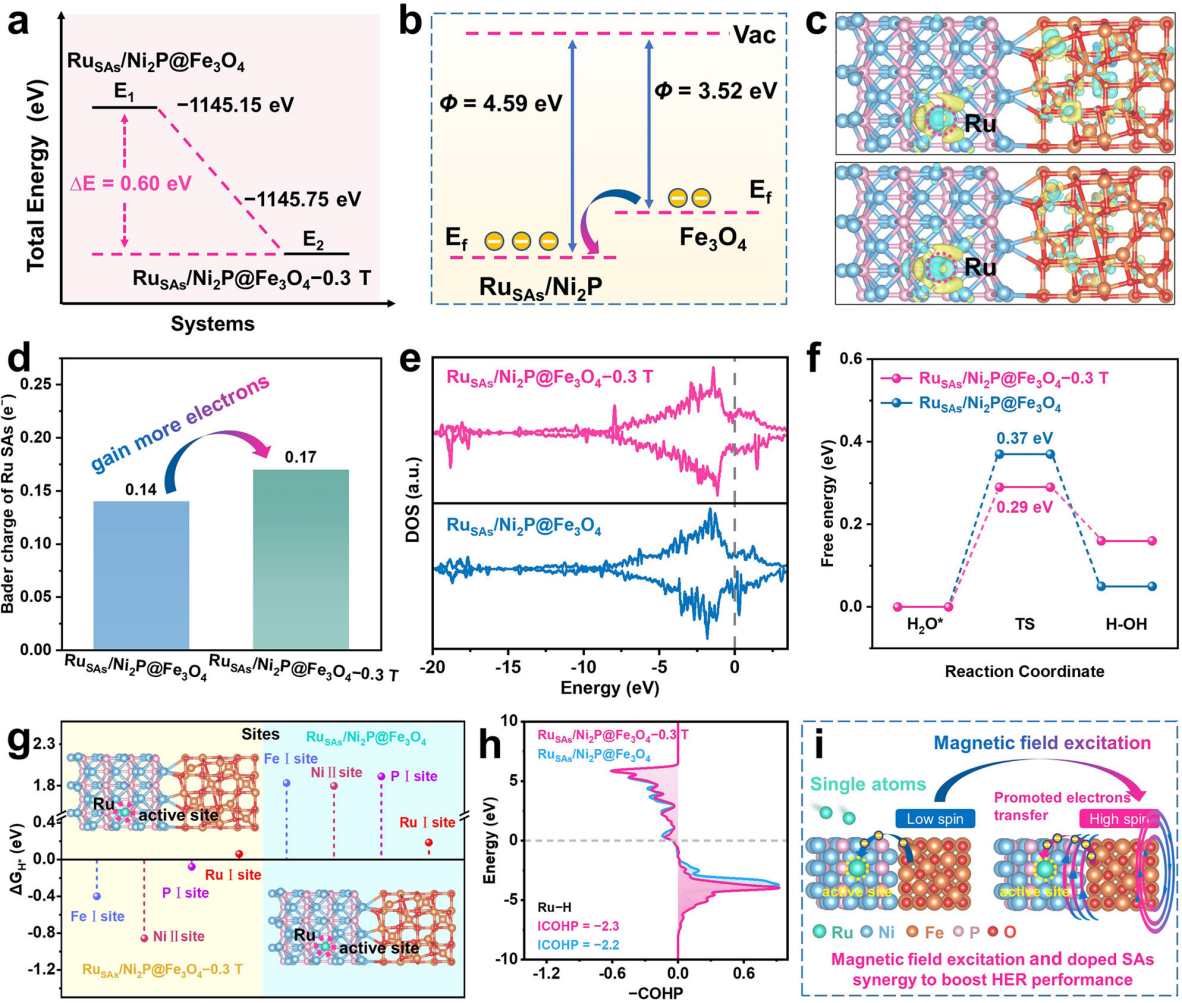
DFT calculations of Ru_SAs_/Ni_2_P@Fe_3_O_4_ and Ru_SAs_/Ni_2_P@Fe_3_O_4_−0.3 T. a) Total energy comparison diagram of Ru_SAs_/Ni_2_P@Fe_3_O_4_ and Ru_SAs_/Ni_2_P@Fe_3_O_4_−0.3 T. b) Schematic diagram of electron transfer in the Ru_SAs_/Ni_2_P@Fe_3_O_4_ structure. c) 3D charge density (Upper figure: Ru_SAs_/Ni_2_P@Fe_3_O_4_, Lower figure: Ru_SAs_/Ni_2_P@Fe_3_O_4_−0.3 T), and d) Bader charge values of Ru sites in Ru_SAs_/Ni_2_P@Fe_3_O_4_ and Ru_SAs_/Ni_2_P@Fe_3_O_4_−0.3 T. Blue area represents the electron‐accumulated region and yellow area represents the electron‐depleted region. (The isosurface value of the plot is 0.008 e bohr^−3^). e) DOS of Ru_SAs_/Ni_2_P@Fe_3_O_4_ and Ru_SAs_/Ni_2_P@Fe_3_O_4_−0.3 T. f) Relative energy profile of water dissociation on Ru sites of Ru_SAs_/Ni_2_P@Fe_3_O_4_ and Ru_SAs_/Ni_2_P@Fe_3_O_4_−0.3 T. g) Comparison plots of ΔG_H*_ at different adsorption sites of Ru_SAs_/Ni_2_P@Fe_3_O_4_ and Ru_SAs_/Ni_2_P@Fe_3_O_4_−0.3 T. h) COHP of adsorbed *H onto Ru site of Ru_SAs_/Ni_2_P@Fe_3_O_4_ and Ru_SAs_/Ni_2_P@Fe_3_O_4_−0.3 T. i) Schematic diagram of the magnetic field excitation and SAs synergistically regulating the catalytic activity of Ru_SAs_/Ni_2_P@Fe_3_O_4_.

Calculations were conducted using heterostructure models of Ru_SAs_/Ni_2_P@Fe_3_O_4_ and Ni_2_P@Fe_3_O_4_. For the Ru_SAs_/Ni_2_P@Fe_3_O_4_ model, stability comparisons were performed for different Ru SAs doping sites, and a Ru_SAs_/Ni_2_P@Fe_3_O_4_ stable structure with lower energy was selected for DFT calculations (Figure S26, Supporting Information). Considering that an external magnetic field can induce spin‐state transitions in Fe atoms, the crystal structure models of Ru_SAs_/Ni_2_P@Fe_3_O_4_−0.3 T under a magnetic field and Ru_SAs_/Ni_2_P@Fe_3_O_4_ without a magnetic field were constructed (Figure S27, Supporting Information). DFT calculations of the total energies for the Ru_SAs_/Ni_2_P@Fe_3_O_4_ and Ru_SAs_/Ni_2_P@Fe_3_O_4_−0.3 T systems reveal that the Ru_SAs_/Ni_2_P@Fe_3_O_4_−0.3 T with an applied 0.3 T magnetic field exhibits a lower total energy (**Figure** **6**a).^[^ ^53^, ^65^ ^]^ This indicates that the magnetic field not only alters the spin state of the material but also stabilizes its structure, which benefits catalytic reactions. Work function studies show a trend of electron transfer from Fe_3_O_4_ to Ru_SAs_/Ni_2_P (Figure 6b; Figure S28, Supporting Information). Additionally, XPS data (Figure S29, Supporting Information) revealed that the binding energy of Ru_SAs_/Ni_2_P@Fe_3_O_4_ shifted to a higher value compared to Fe_3_O_4_, strongly confirming that more electrons are injected from Fe_3_O_4_ into Ru_SAs_/Ni_2_P and highlighting stronger electronic interactions at the interface.^[^ ^66^ ^]^ Among them, electrons tend to accumulate around Ru atoms, resulting in Ru SAs obtaining 0.10 e^−^, which is consistent with the results of XPS and synchrotron radiation analysis. The differential charge density and Bader charge analyses for Ru SAs sites in the Ru_SAs_/Ni_2_P@Fe_3_O_4_ heterostructure (Figure 6c,d) show that electrons migrate from Fe_3_O_4_ to Ru_SAs_/Ni_2_P, causing Ru SAs to gain 0.14 e^−^. The magnetic field further promotes electron transfer to Ru SAs, increasing the electron gain to 0.17 e^−^. Furthermore, XANES characterization results further corroborate this enhanced electron transfer phenomenon. The characterization results show that compared to Ru_SAs_/Ni_2_P@Fe_3_O_4_ without an applied magnetic field, the absorption edge of Ru in Ru_SAs_/Ni_2_P@Fe_3_O_4_−0.3 T shifts toward the Ru foil and is farther from the absorption edge of RuO_2_ (Figure S30, Supporting Information). This spectral shift indicates that Ru in the magnetic field‐treated sample has gained more electrons. Combined with Fe L‐edge XANES characterization data, it can be seen that the Fe absorption peak in Ru_SAs_/Ni_2_P@Fe_3_O_4_−0.3 T shifts more significantly toward the higher energy region compared to that in Ru_SAs_/Ni_2_P@Fe_3_O_4_ (Figure 5a). This indicates that Fe loses electrons. These results further confirm that more electrons are transferred from the Fe_3_O_4_ core to Ru SAs under external magnetic field excitation. The comparison of the electron gain and loss values of Ru SAs in Ni_2_P@Fe_3_O_4_, Ru_SAs_/Ni_2_P@Fe_3_O_4_, and Ru_SAs_/Ni_2_P@Fe_3_O_4_−0.3 T catalysts indicates that an external magnetic field induces more unpaired electrons in the 3d orbital by inducing the spin state of Fe atoms in Fe_3_O_4_ to shift from LS to HS. This significantly enhances the electron transfer kinetics between the Fe_3_O_4_ and Ru_SAs_/Ni_2_P interface, thereby enabling Ru SAs to acquire more charges through interfacial electron coupling.^[^ ^67^, ^68^ ^]^

To deeply analyze the synergistic effect of doped single atoms with Fe 3d orbitals in different spin states, the density of states (DOS) was calculated for Ni_2_P@Fe_3_O_4_, Ru_SAs_/Ni_2_P@Fe_3_O_4_, and Ru_SAs_/Ni_2_P@Fe_3_O_4_−0.3 T (Figure 6e; Figure S31, Supporting Information). The density of states intensity of Ru_SAs_/Ni_2_P@Fe_3_O_4_ near the Fermi level is significantly higher than that of Ni_2_P@Fe_3_O_4_. This phenomenon indicates that Ru SAs doping introduces more local electronic states and enhances the electronic transport capacity at the interface. The density of states intensity of Ru_SAs_/Ni_2_P@Fe_3_O_4_−0.3 T is more prominent than that of Ru_SAs_/Ni_2_P@Fe_3_O_4_ and Ni_2_P@Fe_3_O_4_. This phenomenon indicates that electrons are more accessible near the Fermi level under the intervention of an external magnetic field, greatly enhancing the participation of Ru_SAs_/Ni_2_P@Fe_3_O_4_ in HER‐related electron transfer processes.^[^ ^69^ ^]^ DFT was used to calculate the water dissociation process and different sites of H^*^ adsorption, wherein the calculations for water dissociation were performed with reference to the optimal active sites of H^*^ adsorption. Detailed data on water dissociation can be found in Tables S8–S10 (Supporting Information). Figure 6f and Figure S32 (Supporting Information) results show that the water dissociation energy barrier of Ru_SAs_/Ni_2_P@Fe_3_O_4_ is lower than that of Ni_2_P@Fe_3_O_4_, being 0.37 and 0.48 eV, respectively. This indicates that single‐atom Ru doping shifts the active sites for water dissociation from Ni to Ru and further promotes the water dissociation process. Upon applying a magnetic field, the water dissociation energy barrier of the Ru_SAs_/Ni_2_P@Fe_3_O_4_−0.3 T system further decreases to 0.29 eV, reconfirming that the external magnetic field forms a synergistic effect with single‐atom Ru through inducing spin transitions, collectively optimizing the water dissociation kinetics. The results of hydrogen adsorption free energy (ΔG_H*_) at different active sites in the three systems (Ru_SAs_/Ni_2_P@Fe_3_O_4_−0.3 T, Ru_SAs_/Ni_2_P@Fe_3_O_4_, and Ni_2_P@Fe_3_O_4_) (Figure S33, Supporting Information) demonstrate that in the Ni_2_P@Fe_3_O_4_ system, H* predominantly adsorbs at the P sites of the Ni_2_P shell with a ΔG_H*_ of −0.22 eV (Figure S34a, Supporting Information). This indicates an excessively strong H* adsorption at P sites, which hinders subsequent desorption processes.^[^ ^70^ ^]^ Upon doping with Ru SAs, the active site for H* adsorption shifts to Ru SAs, where ΔG_H*_ decreases to 0.18 eV (Figure S34b, Supporting Information). This confirms that Ru SAs doping optimizes the balance between H* adsorption and desorption,^[^ ^71^ ^]^ in stark contrast to the P sites in Ni_2_P@Fe_3_O_4_. When a 0.3 T magnetic field is applied, the ΔG_H*_ at Ru sites in Ru_SAs_/Ni_2_P@Fe_3_O_4_−0.3 T further improves to 0.06 eV (Figure S34c, Supporting Information). Notably, both Ru_SAs_/Ni_2_P@Fe_3_O_4_−0.3 T and Ru_SAs_/Ni_2_P@Fe_3_O_4_ exhibit optimal ΔG_H*_ values at Ru sites (Figure 6g; Figure S35, Supporting Information), unequivocally identifying Ru as the dominant HER active site in these systems. The magnetic field‐induced transition of Fe atoms from LS to HS states optimizes ΔG_H*_ from 0.18 eV (Ru_SAs_/Ni_2_P@Fe_3_O_4_) to 0.06 eV (Ru_SAs_/Ni_2_P@Fe_3_O_4_−0.3 T), approaching 0 eV. This enhancement originates from the spin‐state transition of Fe atoms under magnetic fields, which modulates the electron localization degree of Ru SAs and strengthens orbital hybridization between Ru SAs and H^*^. Furthermore, calculations of ΔG_H*_ for different sites on the surfaces of Ru_SAs_/Ni_2_P and pure Ni_2_P demonstrate that the ΔG_H*_ at the Ru site in Ru_SAs_/Ni_2_P is 0.19 eV, significantly lower than the ΔG_H*_ of 0.4 eV at the P site in Ni_2_P (Figure S36, Supporting Information). This further confirms that Ru SAs serve as the active sites for HER. Comparison of the ΔG_H*_ values at active sites in the Ru_SAs_/Ni_2_P, Ru_SAs_/Ni_2_P@Fe_3_O_4_, and Ru_SAs_/Ni_2_P@Fe_3_O_4_−0.3 T systems reveals that the enhancement of HER performance induced by spin‐state transitions under a magnetic field is significantly greater than the regulatory effect of heterostructure construction. This indicates that the external magnetic field exerts a more pronounced reinforcing effect on the electronic structure regulation of catalytically active sites by promoting interfacial electron transfer efficiency. To further verify the effect of the external magnetic field on the H^*^ intermediate, the charge density difference and Crystal Orbital Hamilton Population (COHP) of H^*^ adsorbed on the Ru sites of Ru_SAs_/Ni_2_P@Fe_3_O_4_ and Ru_SAs_/Ni_2_P@Fe_3_O_4_−0.3 T were analyzed. As shown by the charge density difference results, compared with Ru_SAs_/Ni_2_P@Fe_3_O_4_, the electron accumulation region of the Ru‐H bond in Ru_SAs_/Ni_2_P@Fe_3_O_4_−0.3 T is more concentrated (Figure S37, Supporting Information). This indicates that in the HS state after applying a magnetic field, the charge transfer and orbital interaction mode between the Ru site and H are altered, which is more conducive to the electron filling of bonding orbitals. Further analysis, combined with the COHP diagram, shows that the integrated crystal orbital Hamilton population (ICOHP) of Ru_SAs_/Ni_2_P@Fe_3_O_4_−0.3 T is −2.3, which is more negative than the ICOHP of Ru_SAs_/Ni_2_P@Fe_3_O_4_ (−2.2) (Figure 6h). Since the larger the absolute value of ICOHP (i.e., the more negative the value), the stronger the chemical bond and the higher the bonding stability. This demonstrates that the bonding interaction of the Ru‐H bond is stronger in the HS state under a magnetic field. This is consistent with the more pronounced electron accumulation region in the charge density difference, confirming that the magnetic field can regulate the spin configuration, affect the orbital hybridization and charge distribution between Ru and ^*^H, and thereby optimize the catalytic reaction process. Specifically, the external magnetic field first induces the spin state transition of Fe_3_O_4_, further facilitating electron transfer at the interface between Fe_3_O_4_ and Ru_SAs_/Ni_2_P. This process enables the Ru active sites to acquire more electrons, and eventually enhances the bonding strength between Ru and the catalytic intermediate H^*^, achieving the optimization of the HER process. Therefore, single atoms and the external magnetic field synergistically regulate the kinetic equilibrium of water dissociation and hydrogen adsorption, ultimately achieving an improvement in HER rate.

Based on the aforementioned findings, the incorporation of Ru SAs into Ni_2_P@Fe_3_O_4_ successfully introduces new active centers for the HER. Since the HER involves substantial electron participation, the applied magnetic field plays a crucial role in promoting electron transfer. Specifically, the magnetic field induces a transition of Fe atoms from LS to HS states, triggering electron redistribution within the Ru_SAs_/Ni_2_P@Fe_3_O_4_ core‐shell structure. This electronic redistribution increases the electron density around the Ru active sites during HER, strengthens the bonding interaction in the Ru‐H intermediate, and thereby accelerates the reaction kinetics (Figure 6i). The synergistic interplay between Ru SAs doping and external magnetic fields establishes an efficient electron‐transport and reaction‐promotion mechanism. These two effects mutually reinforce each other, leading to significantly enhanced HER performance. This work not only provides a solid theoretical foundation but also offers practical guidance for designing high‐performance HER catalysts through spin‐state engineering combined with atomic‐scale doping strategies.

### 2.5 Discussion

This study investigates the impact of an external magnetic field excitation enhancement strategy on the spin‐orbit splitting and catalytic performance of materials by designing a ferromagnetic Ru_SAs_/Ni_2_P@Fe_3_O_4_ core‐shell structure. Through systematic performance evaluation, comprehensive characterization, and theoretical calculations, the unique catalytic mechanism of this innovative ferromagnetic material in the HER is elucidated. The research results show that in the HER reaction, the participation of a high density of electrons at the active sites is crucial, and the external magnetic field plays a key role in promoting the transfer of electrons to the active site. This discovery provides a fundamental basis for understanding how magnetic properties influence catalytic activity.

The external magnetic field induces a transformation of Fe atoms in the ferromagnetic Fe_3_O_4_ from an LS to an HS state, enhancing structural stability and dramatically altering electron distribution within the Ru_SAs_/Ni_2_P@Fe_3_O_4_ material. Specifically, this study reveals through XANES, Mössbauer spectroscopy, EPR, and SQUID measurements that under magnetic field excitation, electrons in the LS state of Fe^3+^ (3d t_2g_) are excited to the HS state (3d e_g_), thereby generating more unpaired electrons in the 3d orbitals. These unpaired electrons are highly active, facilitating electron transfer from Fe_3_O_4_ to Ru_SAs_/Ni_2_P, with electron accumulation around Ru SAs. This phenomenon is intuitively demonstrated by differential charge density and Bader charge analysis. Furthermore, DOS analysis further confirms the regulatory effect of the external magnetic field on the electronic structure. That is, near the Fermi level, the density of states intensity under the action of the external magnetic field is more prominent, indicating that electrons are more easily accessible in this region, which is beneficial for enhancing the participation in the HER‐related electron transfer process.

In the Ru_SAs_/Ni_2_P@Fe_3_O_4_ heterostructure, the incorporation of Ru SAs not only introduces new active sites but also modulates the electronic structure of the heterostructure. DFT calculations show that the introduction of Ru SAs not only changes the degree of electron localization but also affects the electron transfer and reactive sites of the entire catalytic system. The magnetic domains of Fe_3_O_4_, under the influence of an external magnetic field, enhance the efficiency of interfacial electron transfer, thereby enabling Ru SAs to acquire more electrons. Due to the high electron density around Ru SAs, this enhances the bonding interaction of the Ru‐H bond, resulting in the Ru sites exhibiting excellent adsorption‐desorption equilibrium capability for key intermediates in the HER, thus creating favorable conditions for the efficient progression of the HER.

## 3 Conclusion

In conclusion, this study designed a ferromagnetic Ru_SAs_/Ni_2_P@Fe_3_O_4_ core‐shell material and innovatively proposed a synergistic strategy combining single‐atom doping with magnetic field‐driven spin‐state transition, achieving remarkable enhancement of HER catalytic performance. Both theoretical and experimental evidence demonstrate that this strategy not only provides new Ru SAs active sites for the HER reaction but also promotes the transition of Fe^3+^ LS state electrons to a HS state, optimizing electron distribution in the heterostructure. This unique optimization of electronic structure significantly promotes the directional migration of electrons to Ru active sites, strengthens the bonding interaction of the Ru‐H bond, and enables the catalyst to achieve an extremely low overpotential of 38.9 mV at a current density of 10 mA cm^−2^, thus exhibiting excellent catalytic efficiency. This research offers valuable insights for overcoming the performance limitations of nanomaterials and establishes a solid theoretical and practical foundation for the design of advanced HER catalysts. From the perspective of practical application, in the future, the intermittent magnetic field application modes can also be realized by adjusting magnetic field application parameters (such as magnetic field intensity, application interval, single‐action duration, etc.). This approach can not only significantly reduce the energy consumption caused by continuous magnetic field application, but also ensure the long‐term stable operation of the catalytic system. Future investigations will explore the applicability of this strategy in other catalytic reactions, aiming for further advancements in energy conversion and storage.

## 4 Experimental Section

### Materials and Chemicals

Anhydrous ferric chloride (FeCl_3_), nickel nitrate hexahydrate (Ni(NO_3_)_2_·6H_2_O), polyvinylpyrrolidone (PVP), urea (CO(NH_2_)_2_), and sodium hypophosphite (NaH_2_PO_2_) were sourced from Shanghai Aladdin. Co. Ltd. Ethyl alcohol and isopropyl alcohol were acquired from Beijing Chemical Factory. Deionized water was utilized for the synthesis process. All chemicals employed were of analytical grade and used without further purification. The Teflon autoclave (100 mL) was obtained from Anhui Chem‐n Instrument Co., Ltd.

### Physical and Chemical Characterizations

The crystal phases and compositions of the synthesized samples were characterized by X‐ray diffraction (XRD) on a Rigaku D/MAX 2550 Diffractometer using Cu Kα radiation (λ = 1.5418 Å). Microscopic morphologies and elemental distributions were investigated using a combination of scanning electron microscopy (SEM, Zeiss SUPRA 55 SAPPHIRE), transmission electron microscopy (TEM and HRTEM, FEI‐Tecnai G2 F30), spherical aberration‐corrected TEM (AC‐TEM, Thermo Fisher Scientific Themis‐G2), high‐angle annular dark‐field STEM (HAADF‐STEM), and energy‐dispersive X‐ray spectroscopy (EDS) mapping. X‐ray absorption spectroscopy measurements at the Ru K‐edge and Ni K‐edge (XANES and EXAFS) were conducted at the BL 07A beamline of the National Synchrotron Radiation Research Center (Shanghai), while P L‐edge XANES spectra were acquired at beamline BL10B of the National Synchrotron Radiation Laboratory (NSRL, Hefei). The surface chemical states were analyzed by X‐ray photoelectron spectroscopy (XPS, PHI 5000 Versaprobe III), and the bulk elemental composition was determined by inductively coupled plasma optical emission spectrometry (ICP‐OES, Agilent 725 ES). Mössbauer spectroscopy measurements were performed at room temperature with a SEE Co. Model W304 spectrometer system equipped with a ^57^Co(Pd) source. Electron paramagnetic resonance (EPR) spectra were recorded at 100 K on a Bruker EMXplus spectrometer (acknowledgment to eceshi, www.eceshi.com). Magnetic properties were evaluated using a superconducting quantum interference device (SQUID, Quantum Design PPMS‐9T), including zero‐field‐cooled (ZFC) magnetization measurements from 2 to 300 K.

### Synthesis of the Precursor Fe_2_O_3_

Ultrasonically dissolved 0.16 g of FeCl_3_ in 40 mL of deionized water to obtain a solution. Under the condition of magnetic stirring, 40 mL of absolute ethanol was added to this solution, and then sealed and stirred for 15 min. Transferred the entire solution to a 100 mL hydrothermal reaction kettle. After the outer shell was sealed, transfer it to an oven. The reaction temperature was set at 120 °C and lasted for 12 h. Let it cool down naturally. Took the product out of the reaction kettle, and repeatedly centrifuged and washed it with deionized water and absolute ethanol. The obtained solid substance was dried in a vacuum oven, and the powder obtained after drying was the precursor of Fe_2_O_3_.

### Synthesis of Ni(OH)_2_@Fe_2_O_3_

Took 0.05 g of the Fe_2_O_3_ precursor and dispersed it in 10 mL of deionized water to obtain a suspension. Transferred the suspension into a round‐bottomed flask and dispersed it ultrasonically. Then, successively put 0.29 g Ni(NO_3_)_2_·6H_2_O, 1 g PVP, and 1 g CO(NH_2_)_2_ into a beaker. Added 70 mL of absolute ethanol to this beaker. After ultrasonic dissolution, a mixed solution was obtained. Transferred this mixed solution into the round‐bottomed flask containing the Fe_2_O_3_ precursor. Stirred at room temperature for a period of time. After sealing, it was heated in an oil bath at 90 °C for 10 h. Kept stirring and refluxing during the heating process. Let it cool naturally to room temperature. Repeatedly centrifuged and washed the obtained product with deionized water and absolute ethanol. Finally, disperse the product in deionized water to obtain a dispersion. Subjected this dispersion to a freezing treatment. The powder after freeze‐drying was the Ni(OH)_2_/Fe_2_O_3_ core‐shell material.

### Synthesis of Ru_SAs_/Ni(OH)_2_@Fe_2_O_3_

Placed the Ni(OH)_2_/Fe_2_O_3_ core‐shell material in a beaker. Added a certain amount of anhydrous RuCl_3_ aqueous solution to this beaker. Stirred at room temperature for 1 h. Repeatedly centrifuged and washed the product with deionized water and anhydrous ethanol. Finally, disperse the product in 10 mL of deionized water to obtain a dispersion. Subjected this dispersion to a freezing treatment. The powder after freeze‐drying was the Ru_SAs_/Ni(OH)_2_@Fe_2_O_3_ core‐shell material.

### Synthesis of Ru_SAs_/Ni_2_P@Fe_3_O_4_

First, place the Ru_SAs_/Ni(OH)_2_@Fe_2_O_3_ core‐shell material in a porcelain boat. Second, put this porcelain boat at the downstream position of the tube furnace. Then, took NaH_2_PO_2_ and put it into another porcelain boat, and placed this porcelain boat at the upstream position of the tube furnace. The mass ratio of Ru_SAs_/Ni(OH)_2_@Fe_2_O_3_ core‐shell material to NaH_2_PO_2_ was 1:10. Evacuated the tube furnace and then introduced nitrogen for protection. Heated the tube furnace at a rate of 1 °C min^−1^ until it reached 300 °C, and then conducted a phosphating treatment on the Ru_SAs_/Ni(OH)_2_@Fe_2_O_3_ core‐shell material for 2 h. After it cooled down naturally to room temperature, I collected the powder and repeatedly centrifuged and washed it with deionized water and anhydrous ethanol to obtain a solid product. Dried this solid product in a vacuum oven. The powder after drying was the Ru_SAs_/Ni_2_P@Fe_3_O_4_ core‐shell material.

### Synthesis Control Sample: Ru_SAs_/Ni_2_P

First, successively added 0.29 g Ni(NO_3_)_2_·6H_2_O, 1 g PVP, and 1 g CO(NH_2_)_2_ into a flask. Then added 70 mL of absolute ethanol and 10 mL of deionized water. After ultrasonic dissolution, a mixed solution was obtained. Sealed it and heated it in an oil bath at 90 °C for 10 h. During this period, kept stirring and refluxing. Let it cool naturally to room temperature. The product was repeatedly centrifuged and washed with deionized water and absolute ethanol. Finally, dispersed it in 10 mL of deionized water to obtain a dispersion. After the freezing treatment, the freeze‐dried powder was Ni(OH)_2_ nanomaterial. Second, place the Ni(OH)_2_ nanomaterial in a beaker. Added the anhydrous ruthenium chloride aqueous solution in the same steps as those for preparing the Ru_SAs_/Ni(OH)_2_@Fe_2_O_3_ core‐shell material to obtain the Ru_SAs_/Ni(OH)_2_ nanomaterial. Finally, the Ru_SAs_/Ni_2_P nanomaterial was prepared in the same steps as those for preparing the Ru_SAs_/Ni_2_P@Fe_3_O_4_ core‐shell material.

### Synthesis Control Sample: Ni_2_P@Fe_3_O_4_

Placed the Ni(OH)_2_@Fe_2_O_3_ core‐shell material in a porcelain boat, and then put this porcelain boat at the downwind position of the tube furnace. Next, took NaH_2_PO_2_ and put it into another porcelain boat, and placed this porcelain boat at the upwind position of the tube furnace. The mass ratio of the Ni(OH)_2_@Fe_2_O_3_ core‐shell material to NaH_2_PO_2_ was 1:10. Evacuated the tube furnace and then introduced nitrogen for protection. Heated the tube furnace at a rate of 1 °C min^−1^ until it reached 300 °C, and then conducted a phosphating treatment on the Ni(OH)_2_@Fe_2_O_3_ core‐shell material for 2 h. After it cooled down naturally to room temperature, I collected the powder and repeatedly centrifuged and washed it with deionized water and anhydrous ethanol to obtain a solid product. Dried this solid product in a vacuum oven. The powder after drying was the Ni_2_P@Fe_3_O_4_ core‐shell material.

### Theoretical Calculation Section

All calculations for spin polarization were performed using the Vienna Ab initio Simulation Package (VASP).^[^ ^72^ ^]^ The electron interactions of the systems under investigation were accurately described using the generalized gradient approximations (GGA) developed by Perdew‐Burke‐Ernzerhof (PBE).^[^ ^73^ ^]^ The dispersion‐corrected DFT‐D3 schemes were used to describe the interactions between adsorbates and substrates.^[^ ^74^ ^]^ The cut‐off energy was set to 500 eV. A vacuum layer of 20 Å was adopted to simulate the hydrogen evolution reaction (HER) process. The Monkhorst‐Pack method was used for k‐point sampling, with a sampling value of 3 × 3 × 1. The convergence criteria were −0.01 eV Å^−1^ for force and 1.0×10^−5^ eV for energy, respectively. The MAGMOM = Number of ions^*^3 with external magnetic field, and the MAGMOM = Number of ions^*^1 without external magnetic field.

### Electrochemical Measurements

The electrochemical performance was evaluated using an electrochemical workstation (CHI 760E, CH Instruments, Shanghai, China). The hydrogen evolution reaction (HER) performance was evaluated in a 1 m KOH solution. For this assessment, a traditional three‐electrode system was adopted. In this setup, the sample acted as the working electrode. A platinum plate served as the counter electrode, while a saturated Hg/HgO electrode was used as the reference electrode. Specifically, a mixture was first prepared. Its components included 10 µL of Nafion, 2 mg of Ru_SAs_/Ni_2_P@Fe_3_O_4_, 330 µL of deionized water, 330 µL of isopropyl alcohol, and 330 µL of ethanol. This mixture was then applied to carbon paper. After that, the carbon paper with the mixture was dried at room temperature (25 °C). The linear sweep voltammetry (LSV) measurement for HER was performed at a scan rate of 1 mV s^−1^.

The potential of HER and that of the reversible hydrogen electrode (RHE) were calculated according to the following formula:

(1) $$E_{\mathrm{RHE}}=E_{\mathrm{Hg}/\mathrm{HgO}}+0.098+0.0591\times \mathrm{pH}$$

Among them, E_Hg/HgO_ is the measured potential, referring to the Hg/HgO reference electrode.

Electrochemical impedance spectroscopy (EIS) was performed at a potential of −0.1 V vs RHE, covering a frequency range for the hydrogen evolution reaction (HER) from 0.1 Hz to 100 kHz. The electrochemically active surface area (ECSA) of the catalysts was estimated. This estimation was based on the electrochemical double‐layer capacitance (C_dl_) of the catalytic surface. For this process, the following equation was used:

(2) $$\mathrm{ECSA}=\left(C_{\mathrm{dl}}/C_{S}\right)\times S$$

herein, C_dl_ was derived from cyclic voltammetry (CV) measurements, which were dependent on the scan rate. The scan rates ranged from 10 to 80 mV s^−1^, with increments of 10 mV s^−1^. In this regard, S denotes the electrode surface area, with a value of 1 cm^2^. The specific capacitance (C_S_) for a flat surface typically ranges from 20 to 60 µF cm^−2^. In the present work, a C_S_ value of 40 µF cm^−2^ was employed for ECSA estimation.

Additionally, during the electrochemical tests, magnetic fields with strengths of 0.1, 0.2, and 0.3 T were applied to the catalyst via an electromagnet.

## Conflict of Interest

The authors declare no conflict of interest.

## Supporting information



Supporting Information

## Data Availability

The data that support the findings of this study are available from the corresponding author upon reasonable request.
