# Supplementary material for: Magnetic Field‐Driven Spin State Transformation in Promoting the Catalytic Activity of Doped Single‐Atom for Hydrogen Evolution Reaction
Source: Adv Mater. 2025 Nov 7;38(6):e13213. doi: 10.1002/adma.202513213 (PMC12848647; doi:10.1002/adma.202513213)
Supplement: Supplementary file 1 — Supporting Information [file ADMA-38-e13213-s001.docx]

Supporting Information

Magnetic Field-Driven Spin State Transformation in Promoting the Catalytic Activity of Doped Single-Atom for Hydrogen Evolution Reaction

Chenjing Wang^1^, Yuquan Yang^1^, Jinlong Zheng^*1, 2^, Yanru Yuan^1^, Dawei Pang^3^, Jiajia Liu^1^, Hongjing Wu^1^, Naiyan Liu^1^, Hui Ying Yang^*4^, and Xiaolu Pang^*5^

C. Wang, Y. Yang, J. Zheng, Y. Yuan, J. Liu, H. Wu, N. Liu

Beijing Advanced Innovation Center for Materials Genome Engineering, School of Advanced Materials Innovation, University of Science and Technology Beijing, Beijing 100083, China

J. Zheng

Shunde Innovation School, University of Science and Technology Beijing, Foshan 528399, China

E-mail: zhengjinlong@ustb.edu.cn

D. Pang

College of Materials Science and Engineering, Beijing University of Technology, Beijing 100083, China

H. Yang

Department of Materials Science and Engineering, College of Design and Engineering, National University of Singapore, 9 Engineering Drive 1, Singapore 117575, Singapore

E-mail: yanghuiying@nus.edu.sg

X. Pang

School of Materials Science and Engineering, State Key Laboratory of Nuclear Power Safety Technology and Equipment, University of Science and Technology Beijing, Beijing 100083, China

E-mail: pangxl@mater.ustb.edu.cn


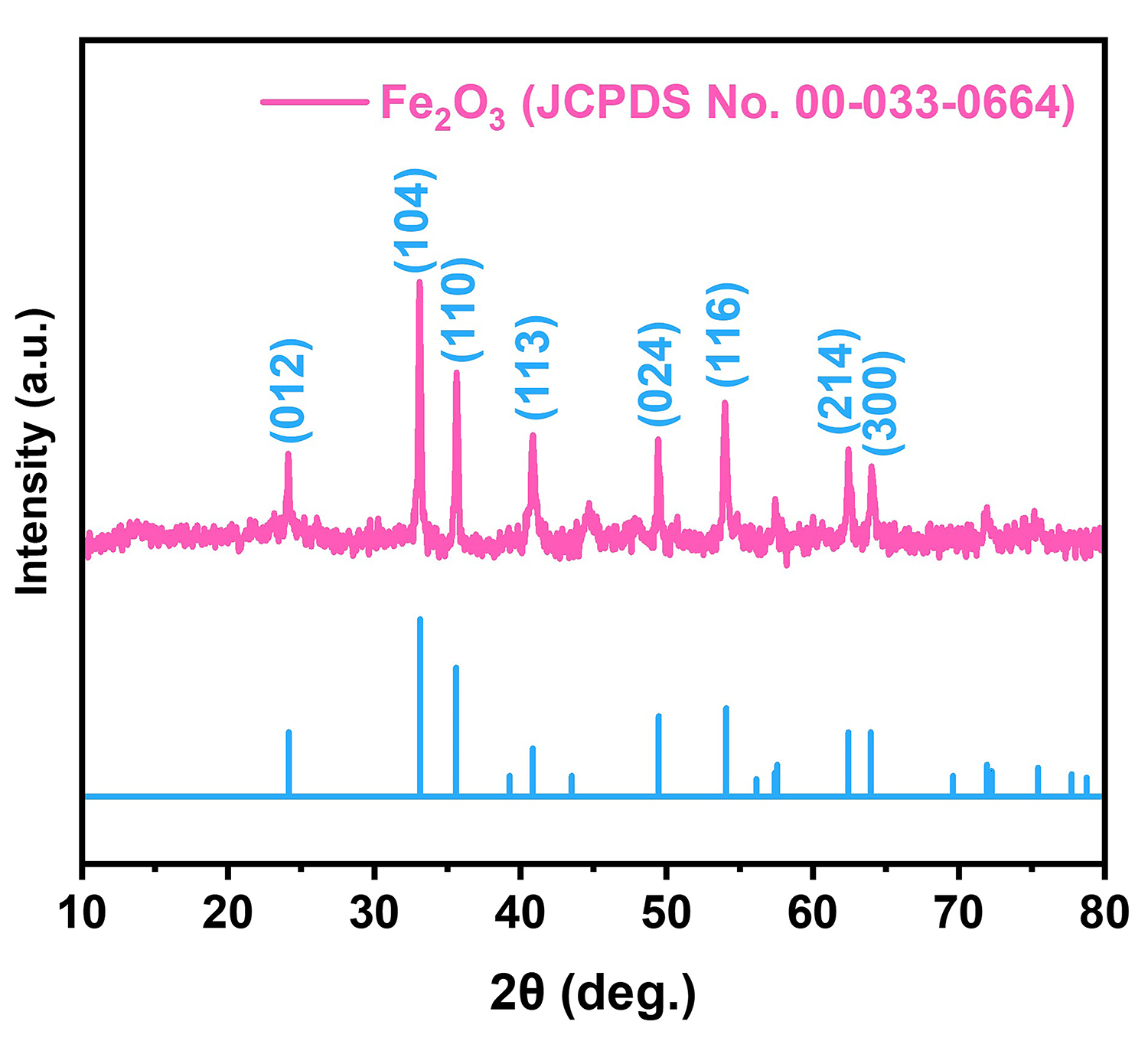


**Figure S1.** XRD pattern of the precursor Fe_2_O_3_.


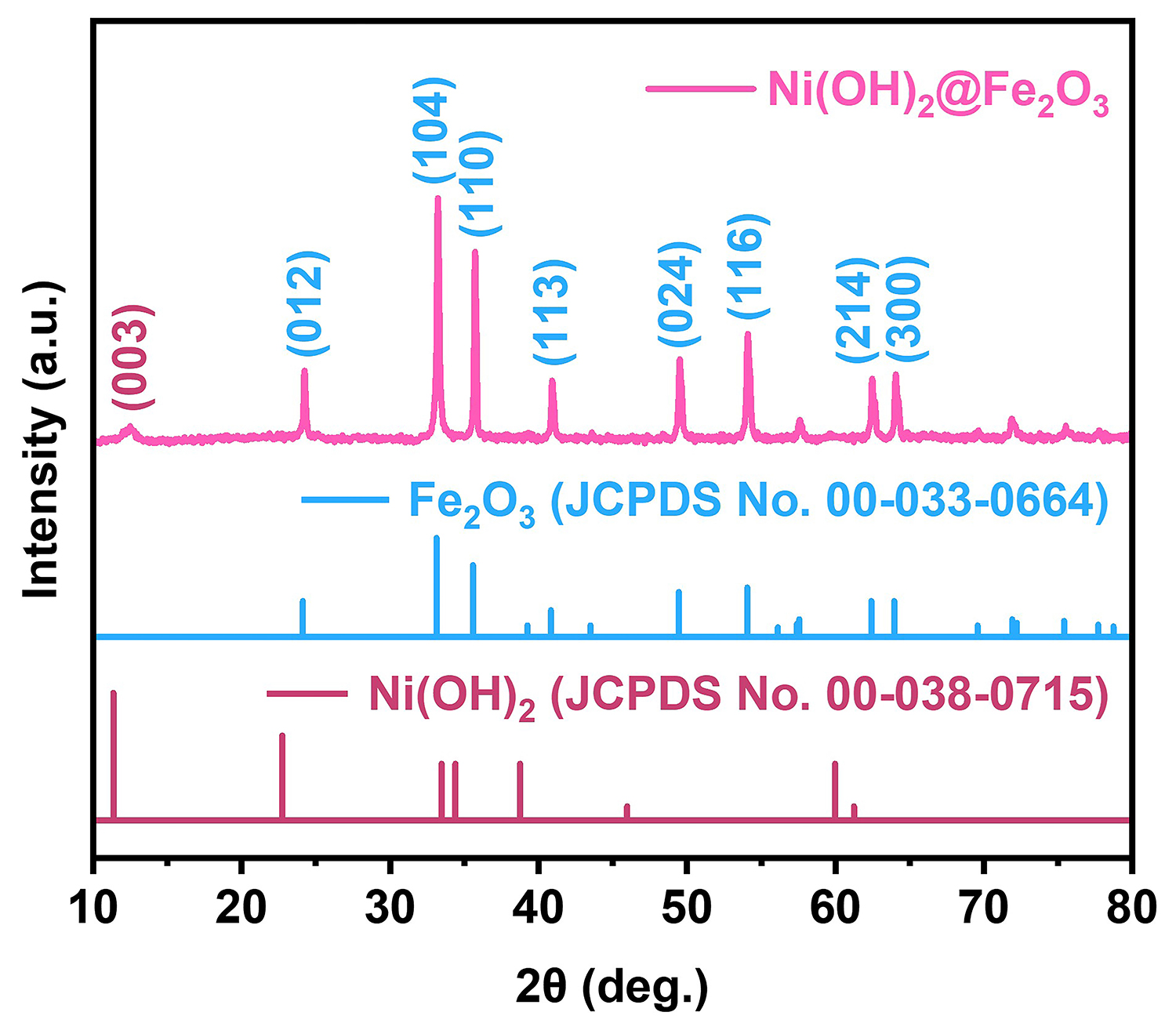


**Figure S2.** XRD pattern of Ni(OH)_2_@Fe_2_O_3_ core-shell structure.


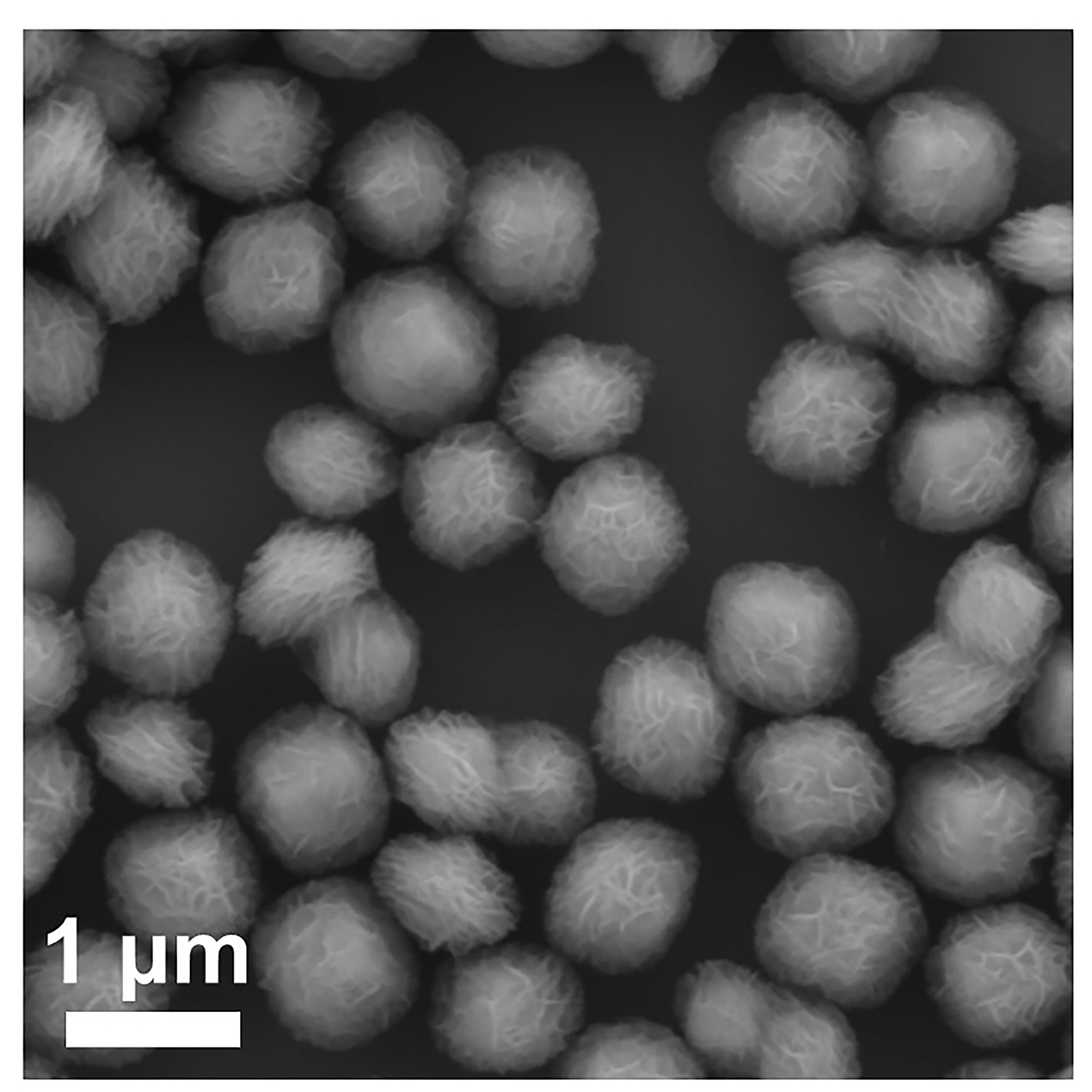


**Figure S3.** SEM image of Ni(OH)_2_@Fe_2_O_3_ core-shell structure.


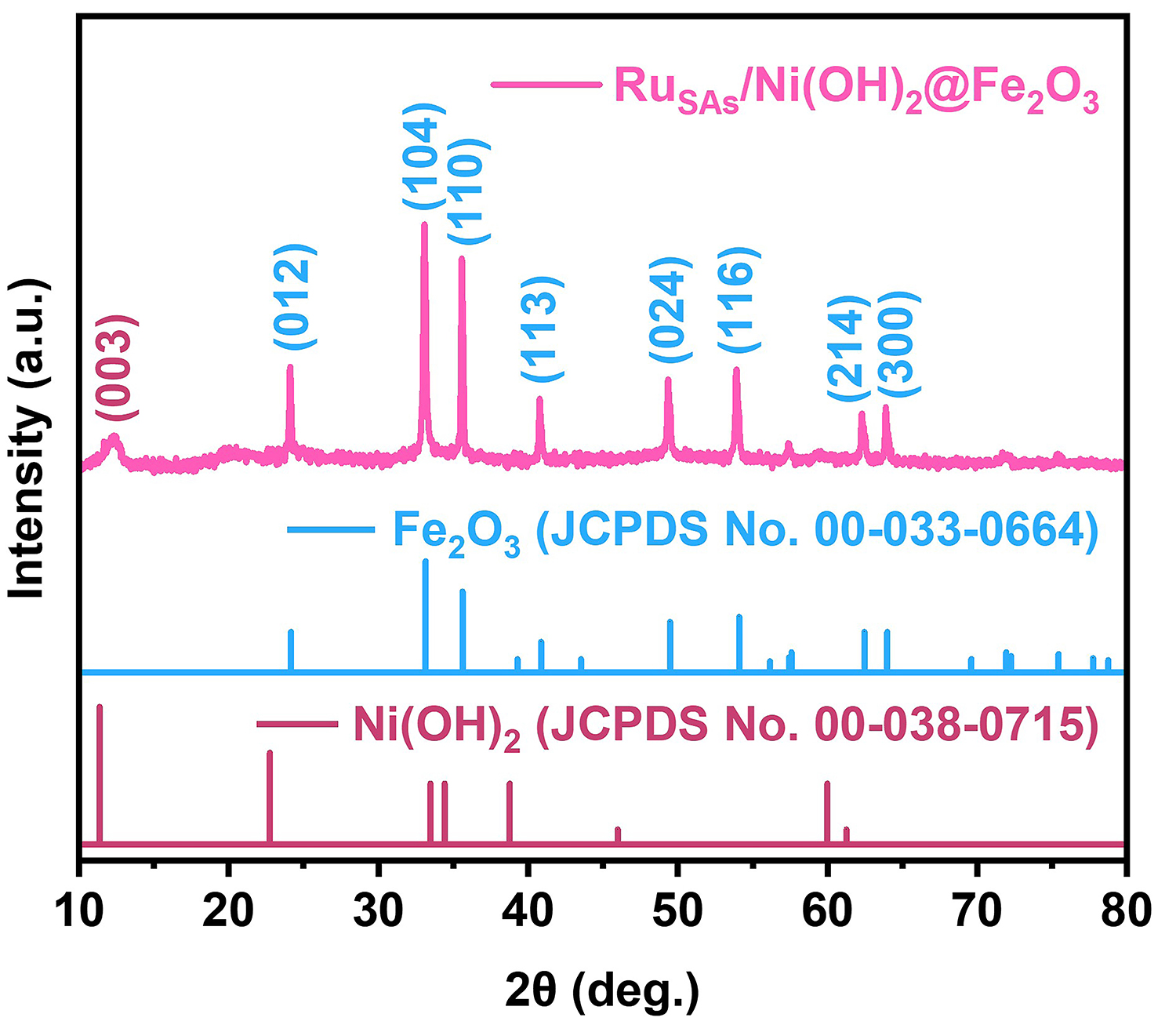


**Figure S4.** XRD pattern of Ru_SAs_/Ni(OH)_2_@Fe_2_O_3_ core-shell structure.

**Table S1.** ICP-OES results of Ru_SAs_/Ni_2_P@Fe_3_O_4_.

| **Test element** | **Test solution element concentration (C_0_ mg L^−1^)** | **Element content (wt %)** |
| --- | --- | --- |
| Fe | 10.85 | 39.89 |
| Ni | 3.85 | 14.14 |
| P | 5.96 | 21.92 |
| Ru | 4.37 | 1.61 |


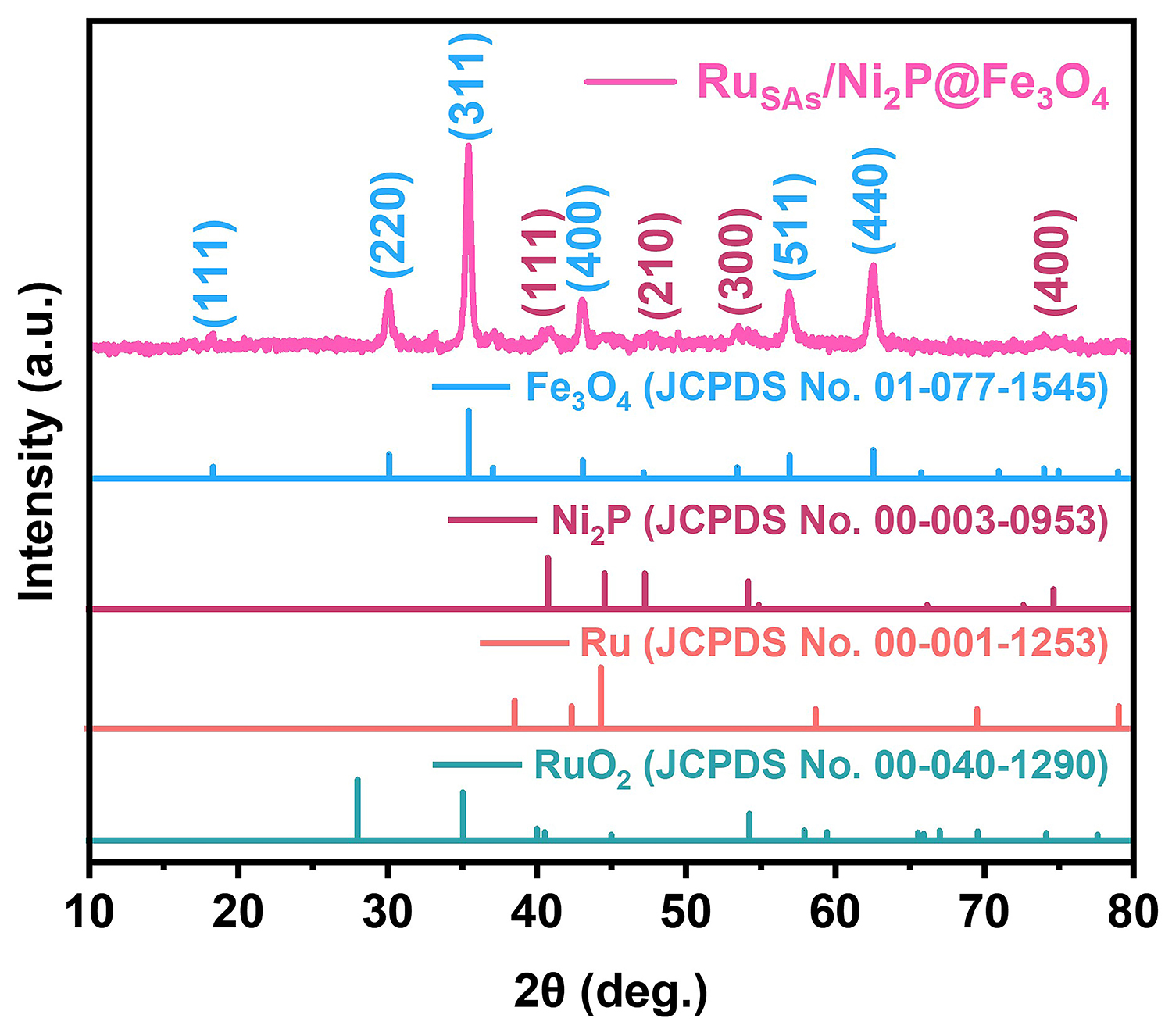


**Figure S5.** XRD pattern of Ru_SAs_/Ni_2_P@Fe_3_O_4_ core-shell structure.


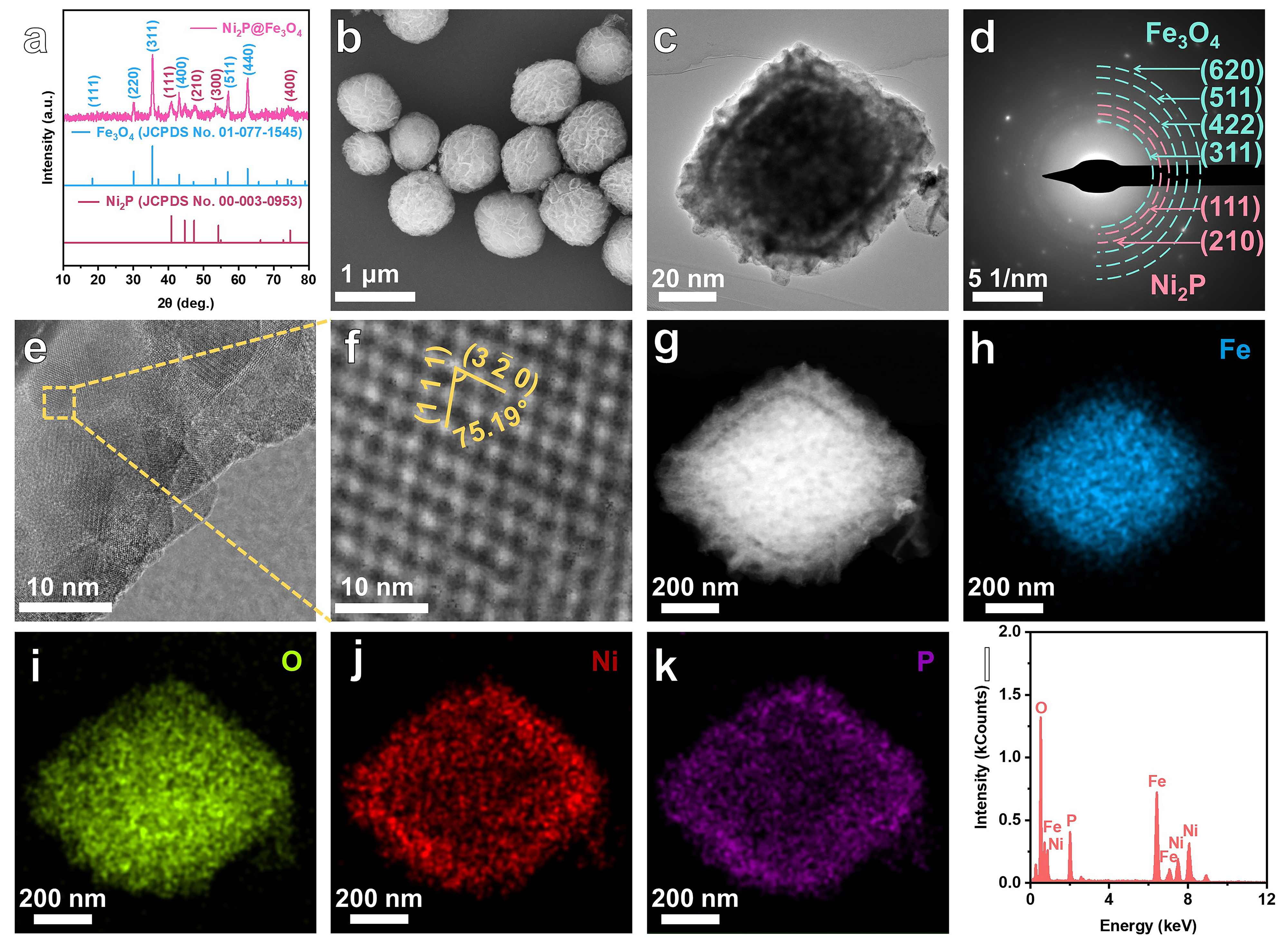


**Figure S6.** Atomic analytical characterization of Ni_2_P@Fe_3_O_4_ core-shell structure. a) XRD pattern. b) SEM image. c) TEM image. d) SAED pattern. e, f) HRTEM images. g) HAADF-STEM image. h-k) Related EDS mapping for elements of Fe, O, Ni, and P. l) EDX spectrum.


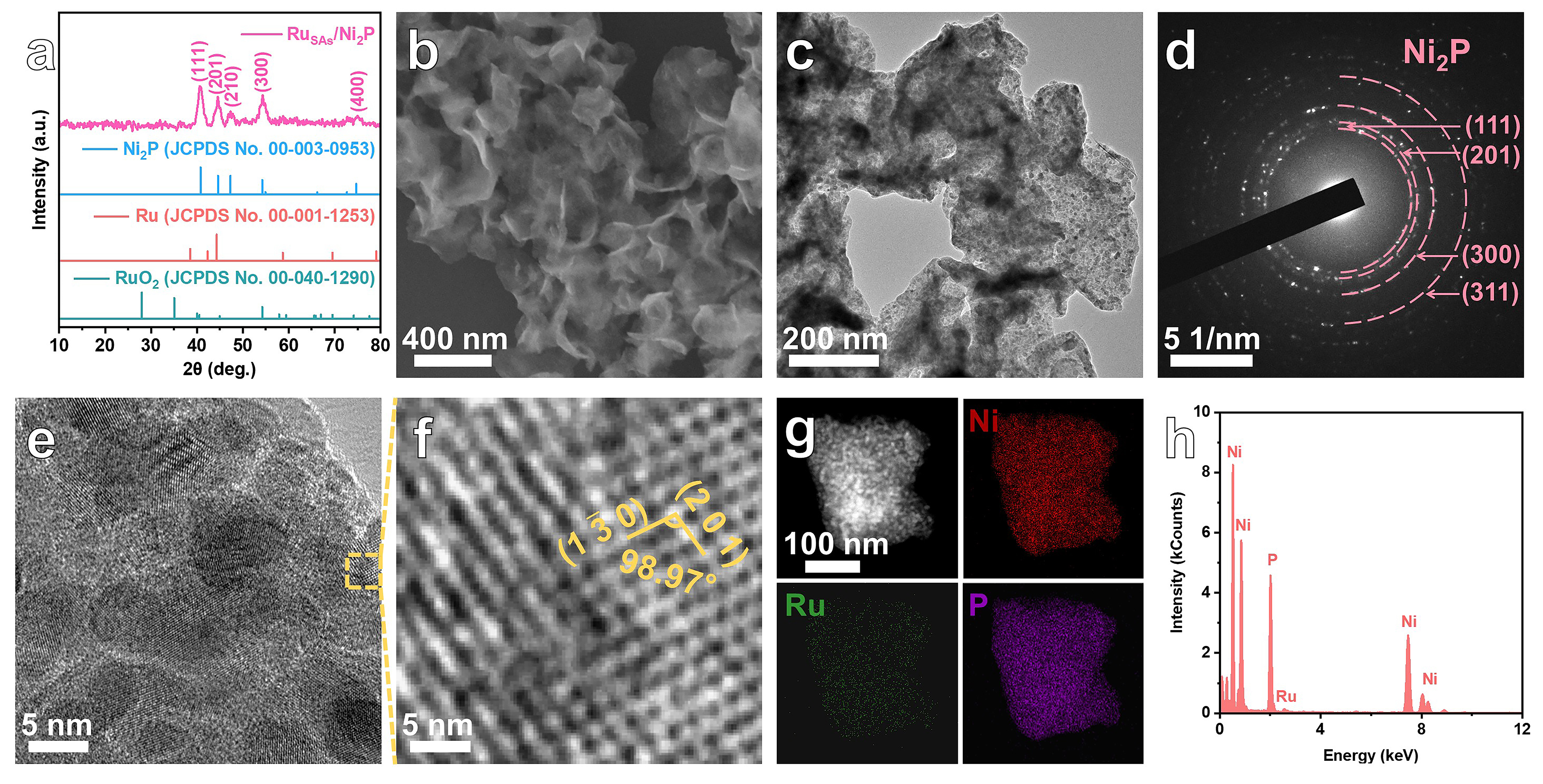


**Figure S7.** Atomic analytical characterization of Ru_SAs_/Ni_2_P materials. a) XRD pattern. b) SEM image. c) TEM image. d) SAED pattern. e, f) HRTEM images. g) HAADF-STEM image and the related EDS mapping for elements of Ni, Ru, and P. h) EDX spectrum.


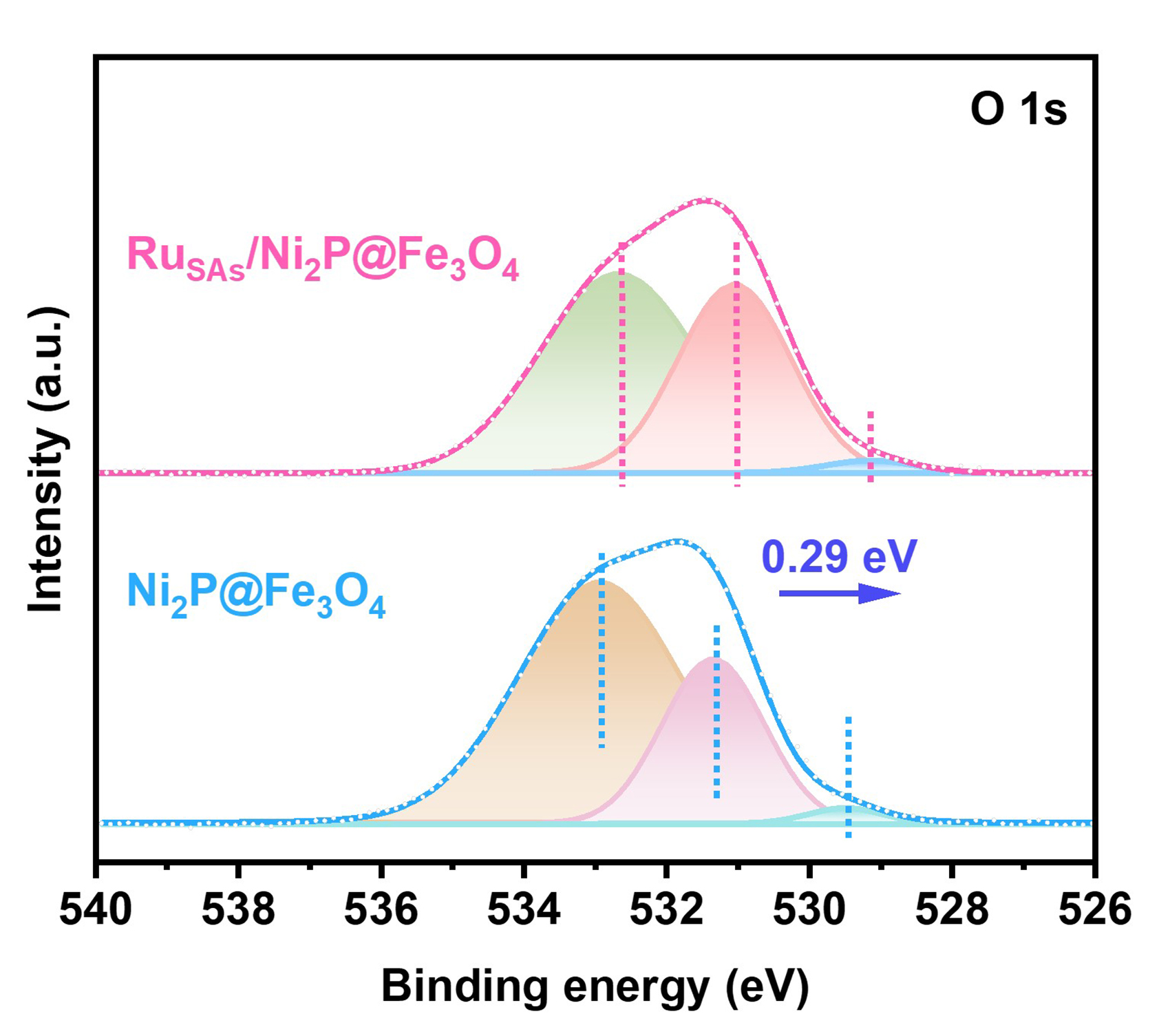


**Figure S8.** High-resolution XPS spectra of O 1s at Ru_SAs_/Ni_2_P@Fe_3_O_4_ and Ni_2_P@Fe_3_O_4_.


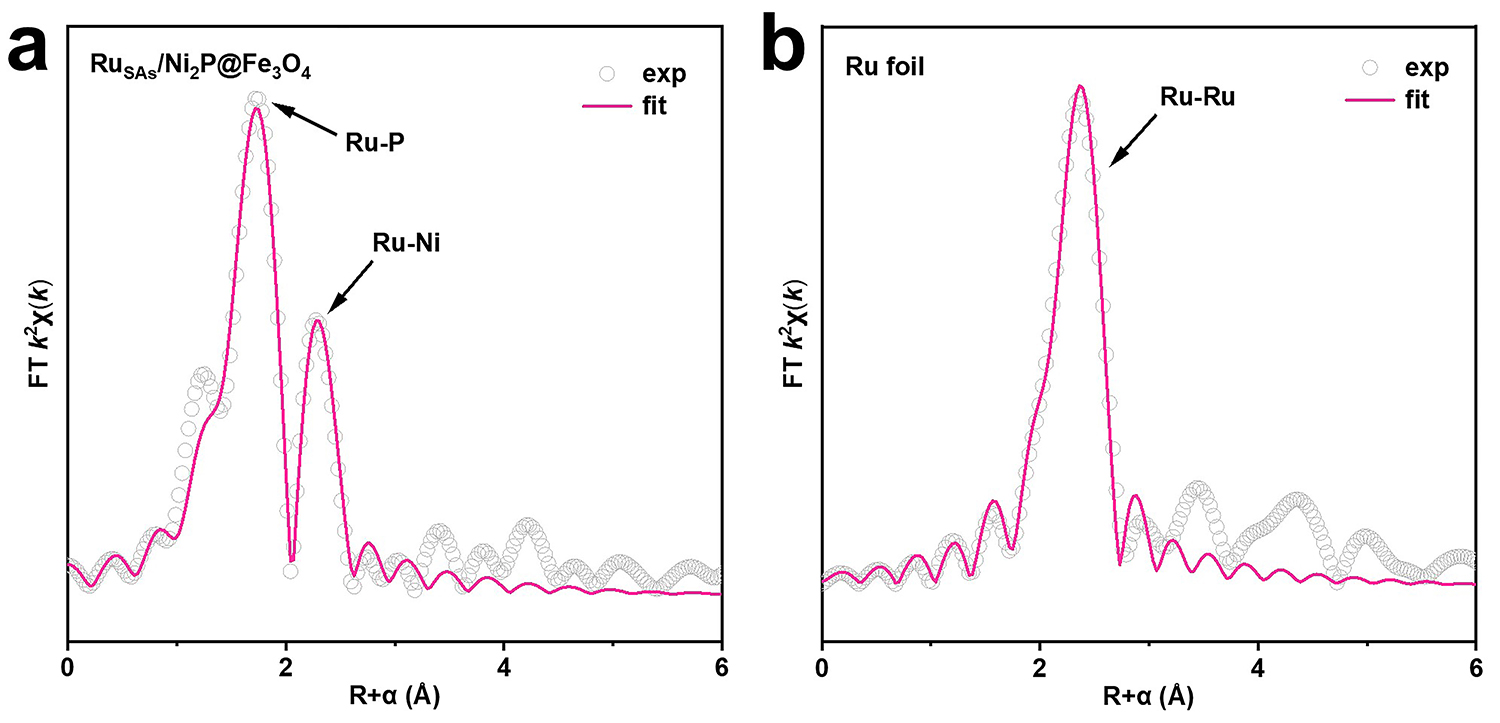


**Figure S9.** R space fitting spectra based on the EXAFS of Ru K-edge for a) Ru_SAs_/Ni_2_P@Fe_3_O_4_, and b) Ru foil.


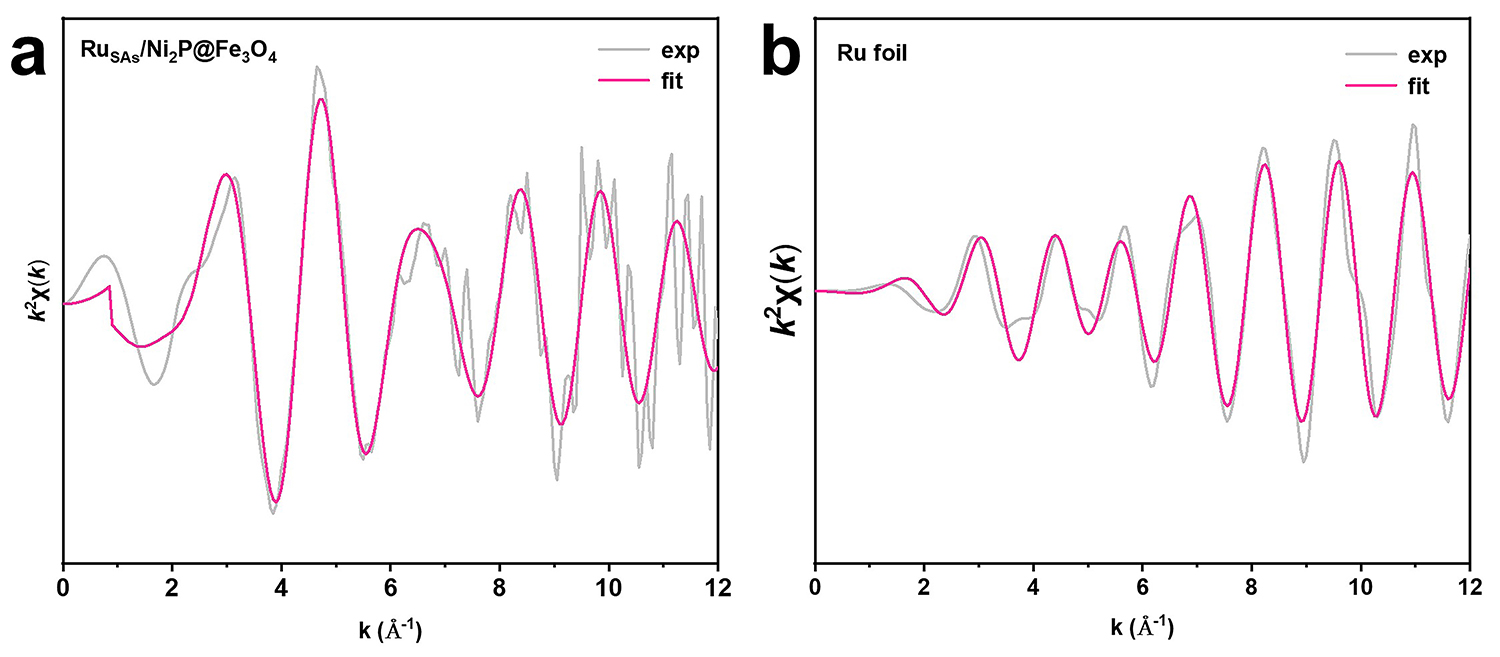


**Figure S10.** K space fitting spectra based on the EXAFS of Ru K-edge for a) Ru_SAs_/Ni_2_P@Fe_3_O_4_, and b) Ru foil.

**Table S2.** EXAFS fitting parameters at the Ru K-edge for various samples (*Ѕ*_0_^2^ = 0.72 from Ru-foil).

| Samples | Shell | CN*^a^* | R*^b^* (Å) | σ^2^*^c^* (Å^2^) | ΔE_0_*^d^* (eV) | R factor |
| --- | --- | --- | --- | --- | --- | --- |
| Ru-foil | Ru-Ru | 12 | 2.67 ± 0.01 | 0.0031 | −5.0 ± 0.9 | 0.0074 |
| Ru_SAs_/Ni_2_P@Fe_3_O_4_ | Ru-P | 3.2 ± 0.3 | 2.34 ± 0.02 | 0.0091 | 3.0 ± 1.4 | 0.0172 |
|  | Ru-Ni | 1.2 ± 0.2 | 2.63 ± 0.02 | 0.0043 |  |  |

*^a^CN*: coordination numbers; *^b^R*: bond distance; *^c^σ*^2^: Debye-Waller factors; *^d^* Δ*E*_0_: the inner potential correction. R factor: goodness of fit. A reasonable range of EXAFS fitting parameters: 0.70 < *Ѕ*_0_^2^ < 1.00; CN > 0; *σ*^2^ > 0 Å^2^; |Δ*E*_0_| < 15 eV; R factor < 0.02.


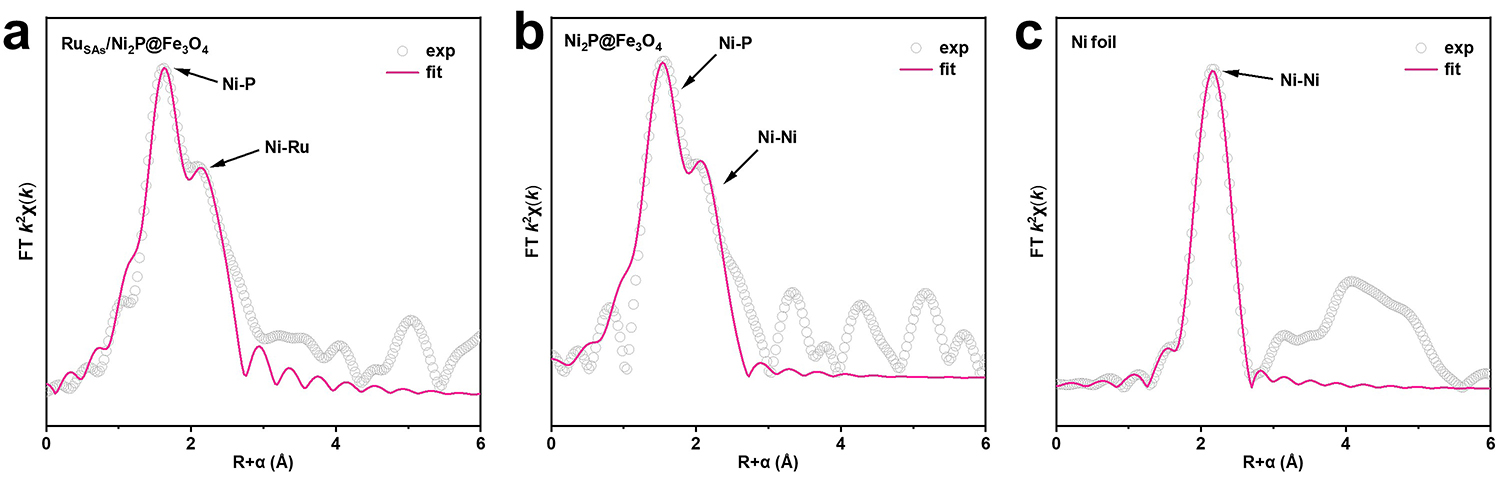


**Figure S11.** R space fitting spectra based on the EXAFS of Ni K-edge for a) Ru_SAs_/Ni_2_P@Fe_3_O_4_, b) Ni_2_P@Fe_3_O_4_, and c) Ni foil.


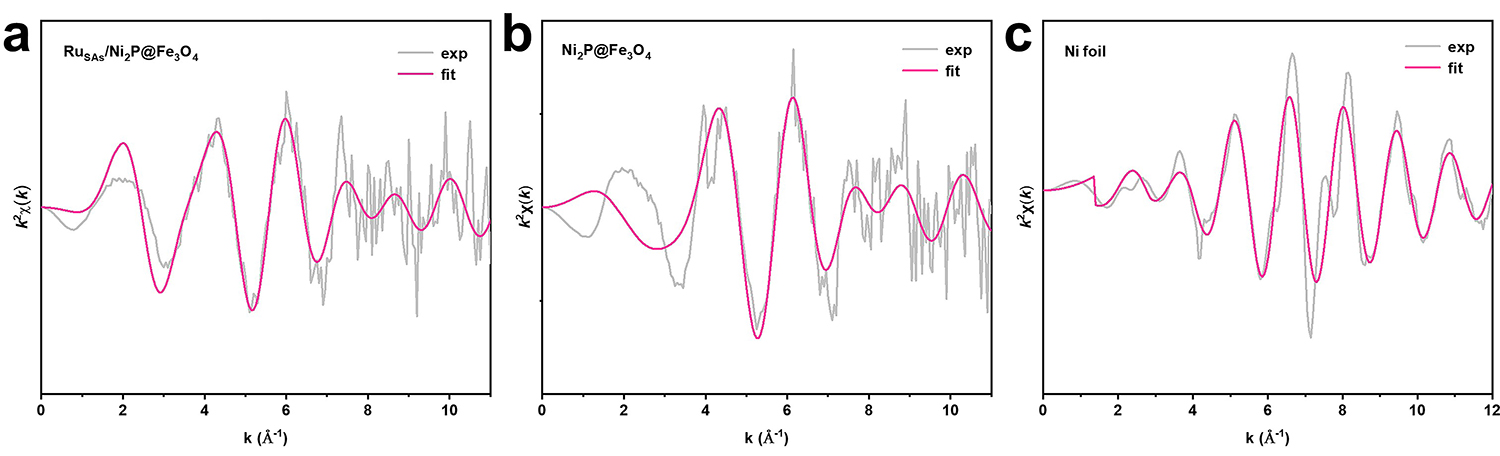


**Figure S12.** K space fitting spectra based on the EXAFS of Ni K-edge for a) Ru_SAs_/Ni_2_P@Fe_3_O_4_, b) Ni_2_P@Fe_3_O_4_, and c) Ni foil.

**Table S3.** EXAFS fitting parameters at the Ni K-edge for various samples (*Ѕ*_0_^2^ = 0.77 from Ni-foil).

| Samples | Shell | CN*^a^* | R*^b^* (Å) | σ^2^*^c^* (Å^2^) | ΔE_0_*^d^* (eV) | R factor |
| --- | --- | --- | --- | --- | --- | --- |
| Ni-foil | Ni-Ni | 12 | 2.48 ± 0.01 | 0.0064 | 7.3 ± 0.7 | 0.0050 |
| Ni_2_P@Fe_3_O_4_ | Ni-P | 3.2 ± 0.3 | 2.08 ± 0.01 | 0.0067 | 3.3 ± 1.6 | 0.0152 |
|  | Ni-Ni | 4.0 ± 0.3 | 2.53 ± 0.01 | 0.0109 |  |  |
| Ru_SAs_/Ni_2_P@Fe_3_O_4_ | Ni-P | 4.2 ± 0.3 | 2.25 ± 0.01 | 0.0090 | −1.0 ± 0.7 | 0.0178 |
|  | Ni-Ru | 3.8 ± 0.5 | 2.60 ± 0.01 | 0.0075 |  |  |

*^a^CN*: coordination numbers; *^b^R*: bond distance; *^c^σ*^2^: Debye-Waller factors; *^d^* Δ*E*_0_: the inner potential correction. R factor: goodness of fit. A reasonable range of EXAFS fitting parameters: 0.70 < *Ѕ*_0_^2^ < 1.00; CN > 0; *σ*^2^ > 0 Å^2^; |Δ*E*_0_| < 15 eV; R factor < 0.02.


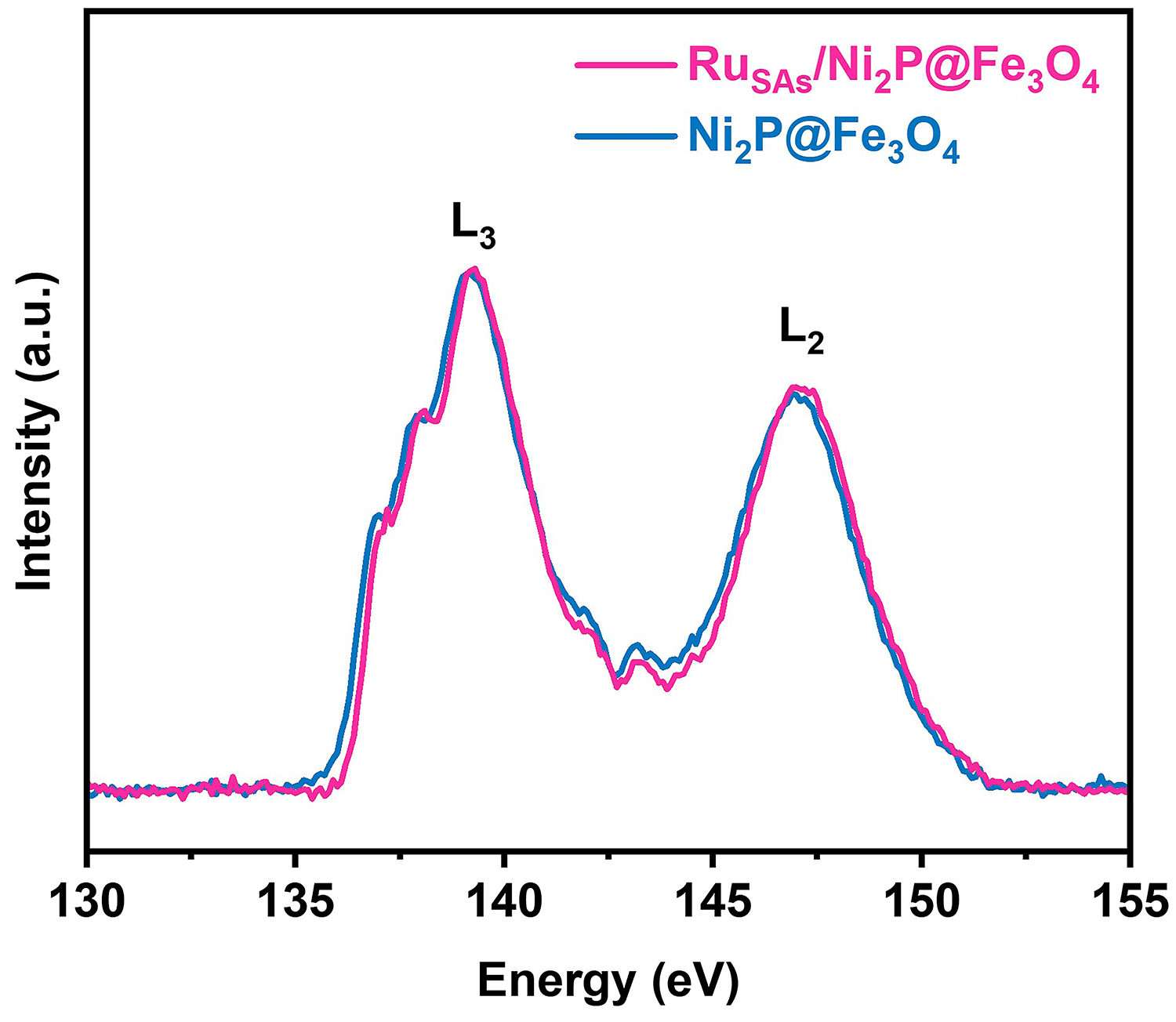


**Figure S13.** P L-edge XANES comparison between Ru_SAs_/Ni_2_P@Fe_3_O_4_ and Ni_2_P@Fe_3_O_4_.


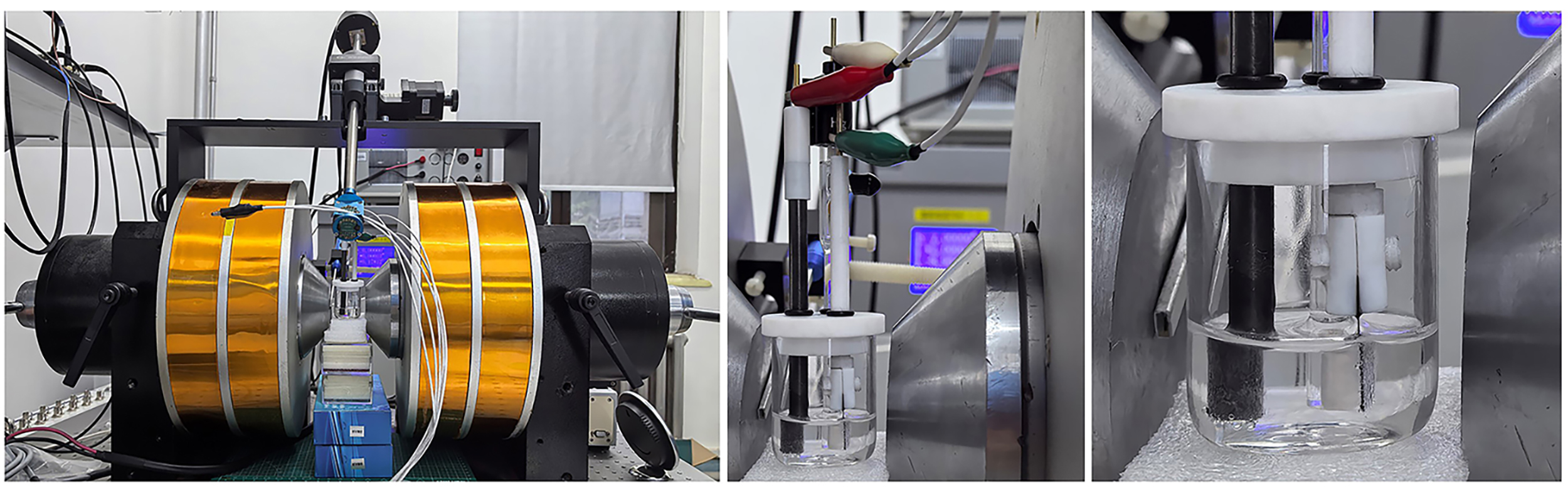


**Figure S14.** Optical images of the electrochemical testing apparatus and external magnetic field generator. (The magnetic field generator can adjust different magnetic field intensities).


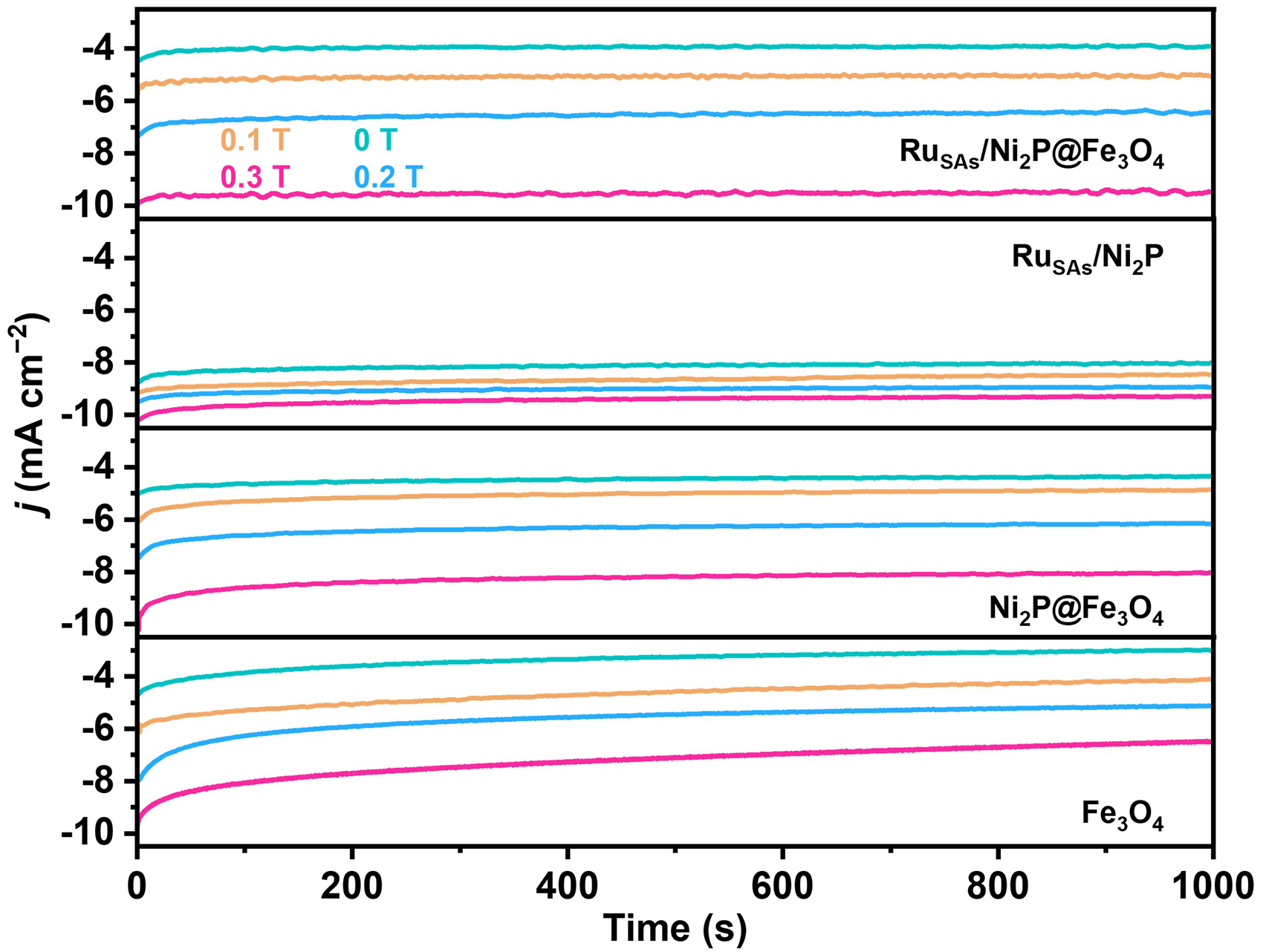


**Figure S15.** I-t curves of Ru_SAs_/Ni_2_P@Fe_3_O_4_, Ru_SAs_/Ni_2_P, Ni_2_P@Fe_3_O_4_, and Fe_3_O_4_ under different magnetic field strengths (0 T, 0.1 T, 0.2 T, and 0.3 T).

Note: In this test, the operational voltage is determined as the potential measured at 10 mA cm^−2^ for Ru_SAs_/Ni_2_P@Fe_3_O_4_−0.3 T (data corresponds to Figure 4b). I-t tests are subsequently carried out at this voltage under the magnetic field strengths of 0 T, 0.1 T, 0.2 T, and 0.3 T, respectively. Similarly, the operational voltages are determined as the potentials measured at 10 mA cm^−2^ for Ru_SAs_/Ni_2_P−0.3 T, Ni_2_P@Fe_3_O_4_−0.3 T, and Fe_3_O_4_−0.3 T (Figure 4b), respectively. I-t tests are then performed at these respective determined voltages under the magnetic fields of 0 T, 0.1 T, 0.2 T, and 0.3 T. (Ru_SAs_/Ni_2_P@Fe_3_O_4_: −0.039 V *vs*. RHE; Ru_SAs_/Ni_2_P: −0.067 V *vs*. RHE; Ni_2_P@Fe_3_O_4_: −0.111 V *vs*. RHE; Fe_3_O_4_: −0.319 V *vs*. RHE)


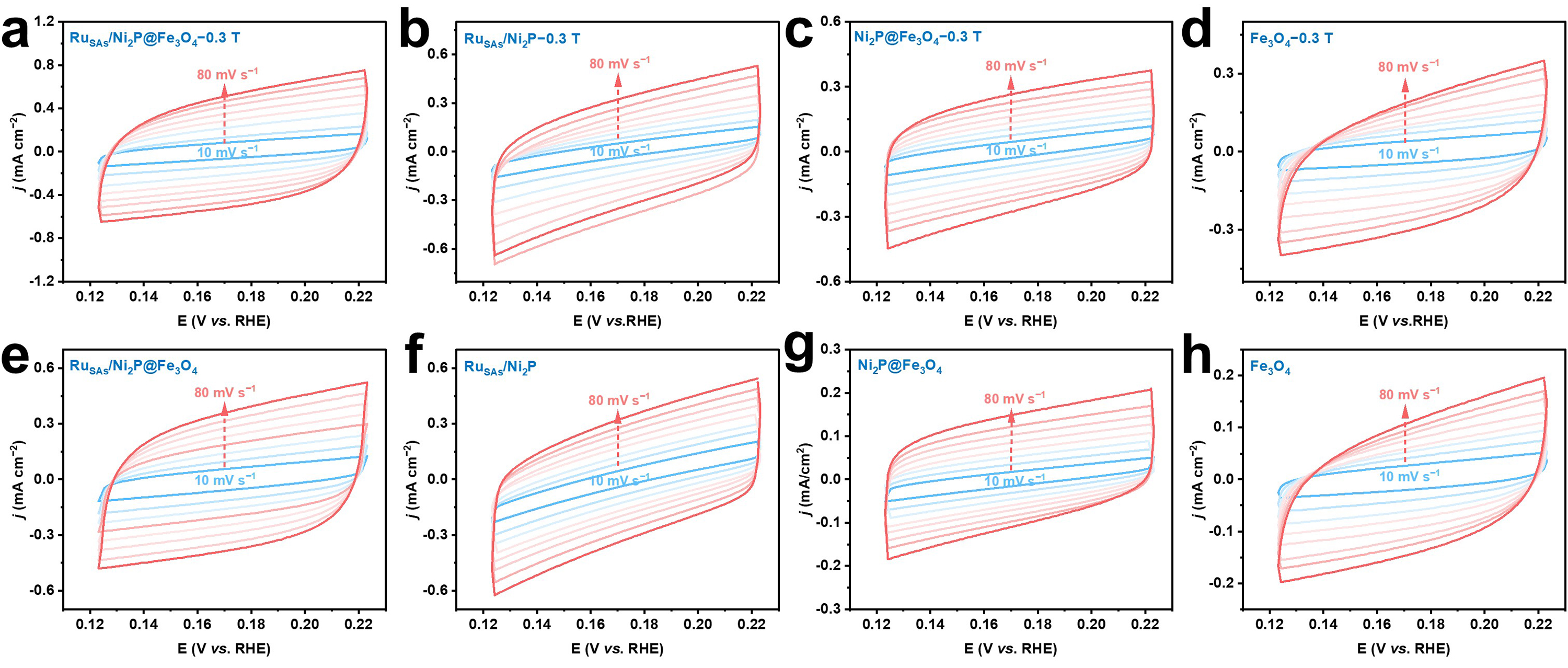


**Figure S16.** CV curves of a) Ru_SAs_/Ni_2_P@Fe_3_O_4_−0.3 T, b) Ru_SAs_/Ni_2_P−0.3 T, c) Ni_2_P@Fe_3_O_4_−0.3 T, d) Fe_3_O_4_−0.3 T, e) Ru_SAs_/Ni_2_P@Fe_3_O_4_, f) Ru_SAs_/Ni_2_P, g) Ni_2_P@Fe_3_O_4_, and h) Fe_3_O_4_ at the range of 0.11 ~ 0.22 V *vs*. RHE with scanning rates of 10, 20, 30, 40, 50, 60, 70 and 80 mV s^−1^ under the conditions with and without a 0.3 T external magnetic field. (The magnetic field strength is 0.3 T).


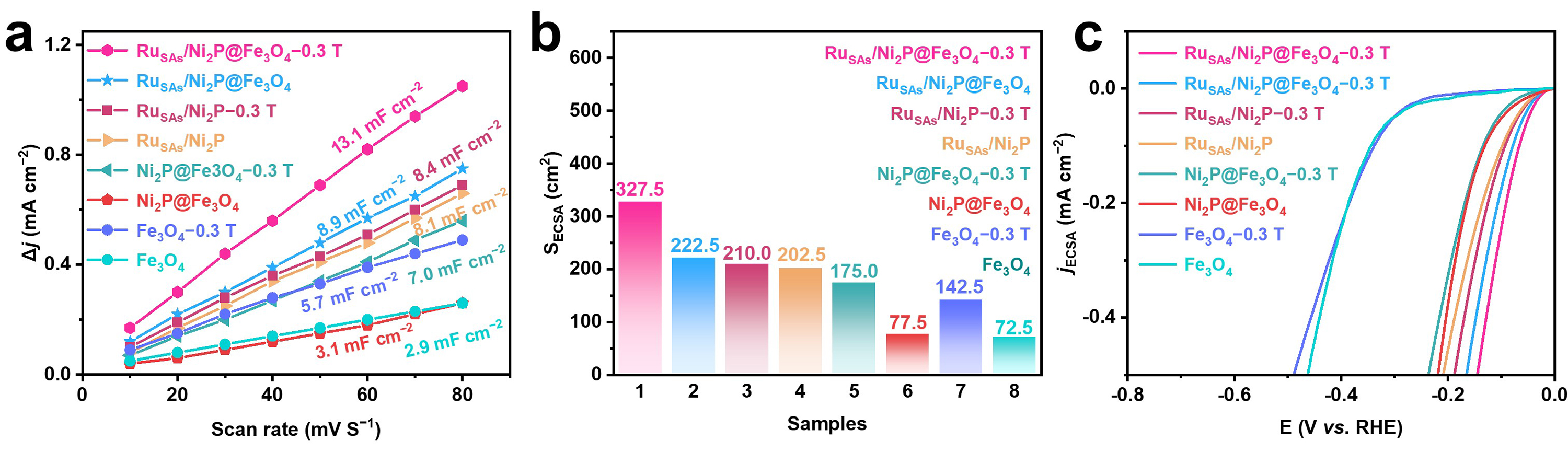


**Figure S17.** a) Double-layered capacitance, b) ECSA values, and c) ECSA-normalized LSV curves of Ru_SAs_/Ni_2_P@Fe_3_O_4_−0.3 T, Ru_SAs_/Ni_2_P@Fe_3_O_4_, Ru_SAs_/Ni_2_P−0.3 T, Ru_SAs_/Ni_2_P, Ni_2_P@Fe_3_O_4_−0.3 T, Ni_2_P@Fe_3_O_4_, Fe_3_O_4_−0.3 T, and Fe_3_O_4_ electrocatalysts under the conditions with and without a 0.3 T external magnetic field.


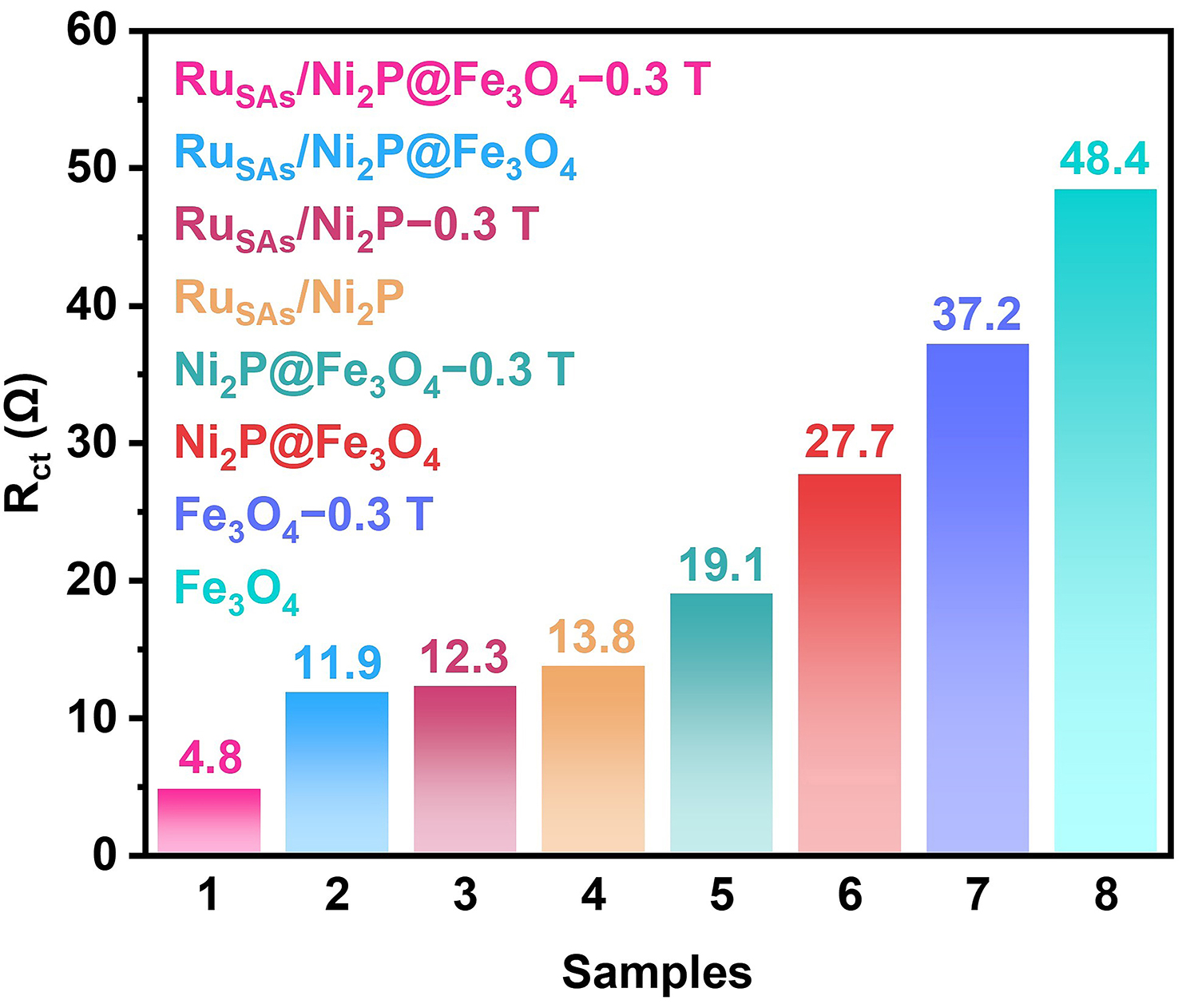


**Figure S18.** R_ct_ values of Ru_SAs_/Ni_2_P@Fe_3_O_4_−0.3 T, Ru_SAs_/Ni_2_P@Fe_3_O_4_, Ru_SAs_/Ni_2_P−0.3 T, Ru_SAs_/Ni_2_P, Ni_2_P@Fe_3_O_4_−0.3 T, Ni_2_P@Fe_3_O_4_, Fe_3_O_4_−0.3 T, and Fe_3_O_4_ (sample 1 to 8) electrocatalysts under the conditions with and without a 0.3 T external magnetic field.


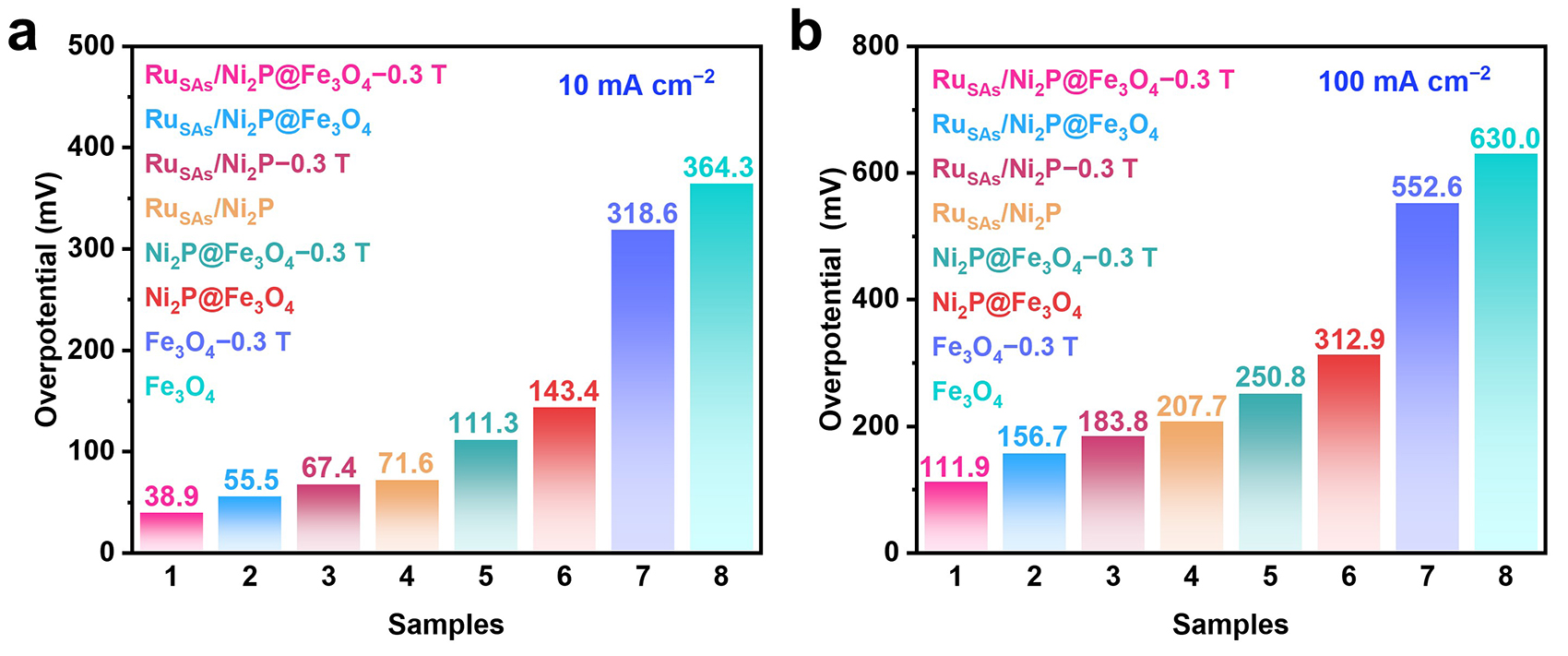


**Figure S19.** Overpotentials of Ru_SAs_/Ni_2_P@Fe_3_O_4_−0.3 T, Ru_SAs_/Ni_2_P@Fe_3_O_4_, Ru_SAs_/Ni_2_P−0.3 T, Ru_SAs_/Ni_2_P, Ni_2_P@Fe_3_O_4_−0.3 T, Ni_2_P@Fe_3_O_4_, Fe_3_O_4_−0.3 T, and Fe_3_O_4_ (sample 1 to 8) electrocatalysts under the conditions with and without a 0.3 T external magnetic field when the current density is a) 10 mA cm^−2^, and b) 100 mA cm^−2^.

**Table S4.** Comparison of the performance between Ru_SAs_/Ni_2_P@Fe_3_O_4_ and Ru_SAs_/Ni_2_P@Fe_3_O_4_−0.3 T with that of the reported Ru SAs, Ni-based, and Fe-based HER catalysts, as well as with other catalysts under the action of an external magnetic field.

| **Catalyst** | **Electrolyte** | **η_10_ (mV)** | **Tafel slope (mV dec^−1^)** | **Reference** |
| --- | --- | --- | --- | --- |
| Ru-Co_2_P@Ru-N-C | 1 M KOH | 69 | 65 | **Adv. Funct. Mater.**, **34, 2024**, 2316709^[1]^ |
| Ru-(Ni/Fe)C_2_O_4_ | 1 M KOH | 42 | 39 | **Appl Catal B: Environ.**, **2023**, 325, 122354^[2]^ |
| A-Fe-CoP/CPN | 1 M KOH | 110 | 101 | **J. Energy Chem.**, **2022**, 71, 36-44^[3]^ |
| NF/CoFeP | 1 M KOH | 80 | 67 | **Nano Lett.**, **2023**, 23, 8331-8338^[4]^ |
| Fe-Co-Ni MOF | 1 M KOH | 116 | 56 | **J. Am. Chem. Soc.**, **2022**, 144, 3411-3428^[5]^ |
| Cu_1_Co_2_-Ni_2_P/NF | 1 M KOH | 51 | 52.3 | **Adv. Mater.**, **2023**, 35, e2305598^[6]^ |
| I-Ni@C | 1 M KOH | 78 | 60 | **J. Am. Chem. Soc.**, **2024**, 146, 26844-26854 ^[7]^ |
| NiCo_2_S_4_/ReS_2_ | 1 M KOH | 85 | 78.3 | **Adv. Funct. Mater.**, **2022**, 33, 2210072^[8]^ |
| Ru-NiPS_3_ NSs | 1 M KOH | 59 | 64 | **Nat. Commun.**, **2023**, 14, 6462^[9]^ |
| MoO_2_/Ni_3_S_2_/NF | 1 M KOH | 74 | 82.5 | **Nano Energy**, **2024**, 122, 109299^[10]^ |
| Ni/Y_2_O_3_ | 1 M KOH | 61.1 | 52.8 | **Adv. Energy Mater.**, **2024**, 14, 2303563^[11]^ |
| Pt SA-NiSe-V | 1 M KOH | 45 | 52 | **Angew. Chem. Int. Ed.**, **2023**, 62, e202308686^[12]^ |
| 0.0039 T Gd@MoS_2_ | 1 M KOH | 57 | 50 | **Small**, **2023**, 19, e2206155^[13]^ |
| 0.3 T Ni_3_Fe-CW | 1 M KOH | 76 | 137 | **Chem. Eng. J.**, **2022**, 439, 135722^[14]^ |
| 0.02 T Fe_29_Co_29_Ni_29_P_3.9_B_9.1_ | 1 M KOH | 350 | 82.5 | **ACS Appl. Mater. Interfaces**, **2022**, 14, 15243-15249^[15]^ |
| **Ru_SAs_/Ni_2_P@Fe_3_O_4_−0.3 T** | **1 M KOH** | **38.9** | **39.5** | **This work** |
| **Ru_SAs_/Ni_2_P@Fe_3_O_4_** | **1 M KOH** | **55.5** | **50.1** |  |


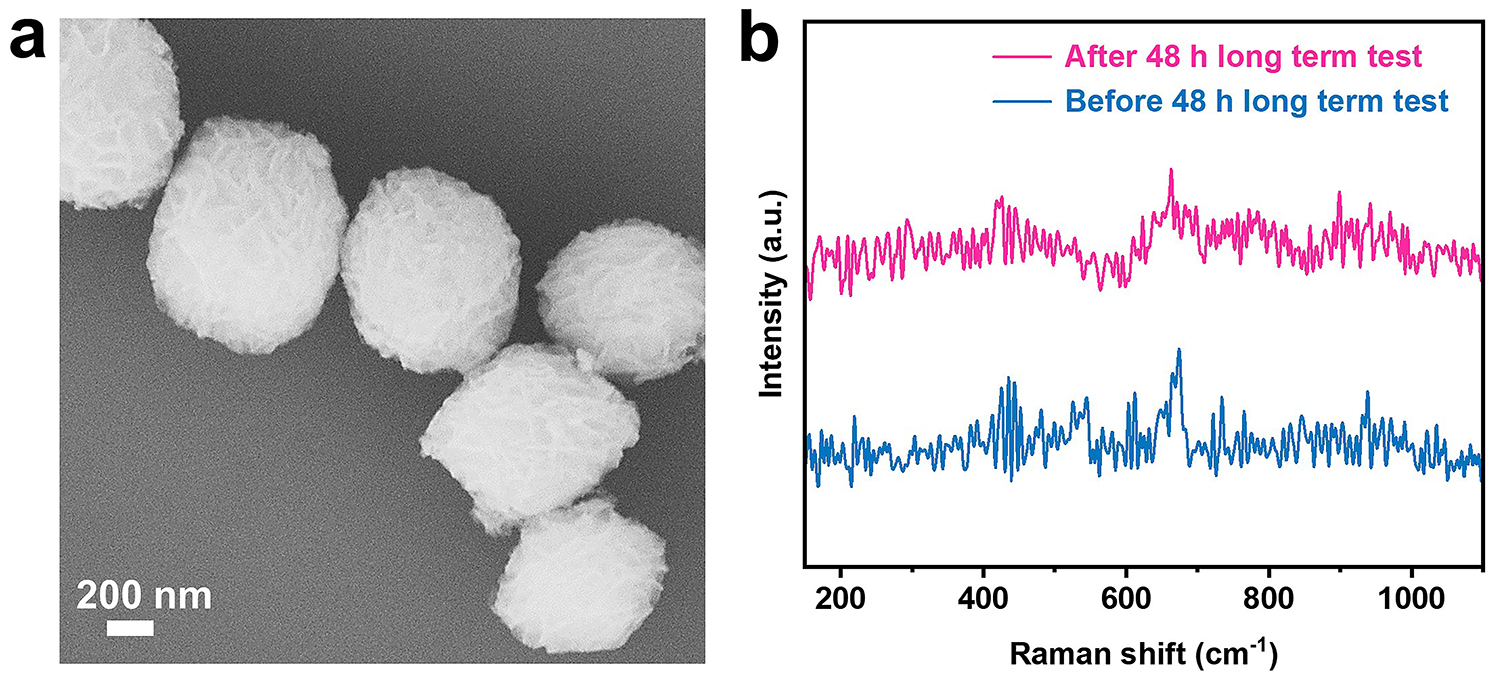


**Figure S20.** a) SEM image and b) Raman of Ru_SAs_/Ni_2_P@Fe_3_O_4_−0.3 T electrocatalysts before and after a 48-hour long-term test under a 0.3 T external magnetic field.


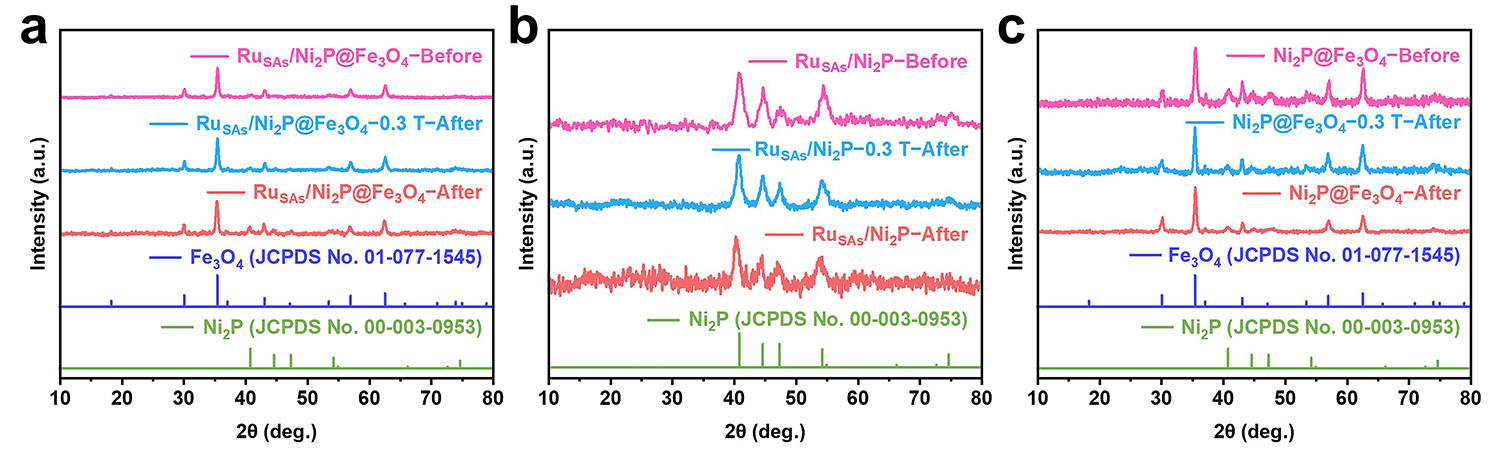


**Figure S21.** XRD patterns of a) Ru_SAs_/Ni_2_P@Fe_3_O_4_, b) Ru_SAs_/Ni_2_P, and c) Ni_2_P@Fe_3_O_4_ electrocatalysts before and after a 48-hour long-term test under the conditions with and without a 0.3 T external magnetic field.


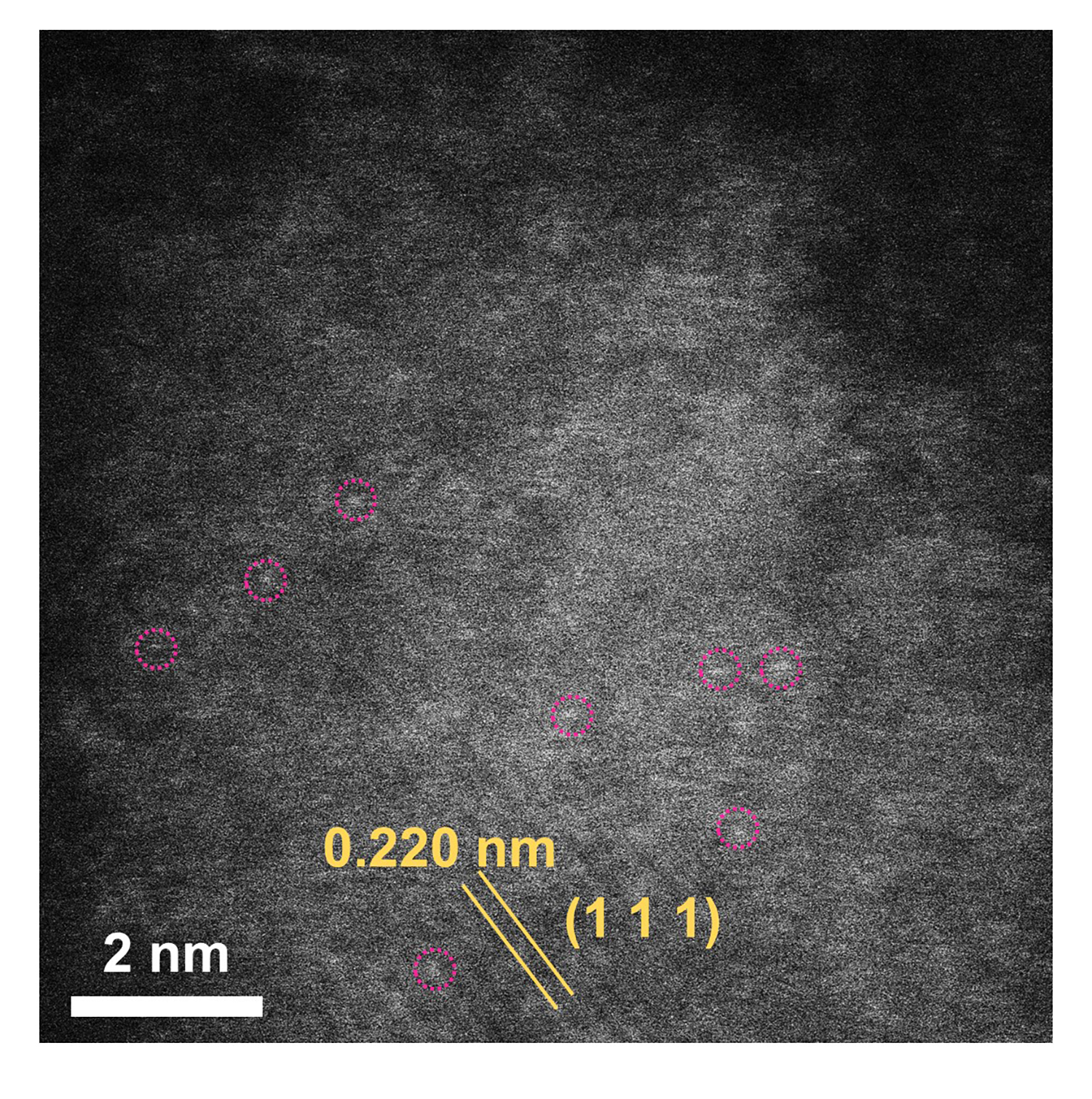


**Figure S22.** AC-HAADF-STEM of Ru_SAs_/Ni_2_P@Fe_3_O_4_−0.3 T electrocatalysts after a 48-hour long-term test under a 0.3 T external magnetic field.


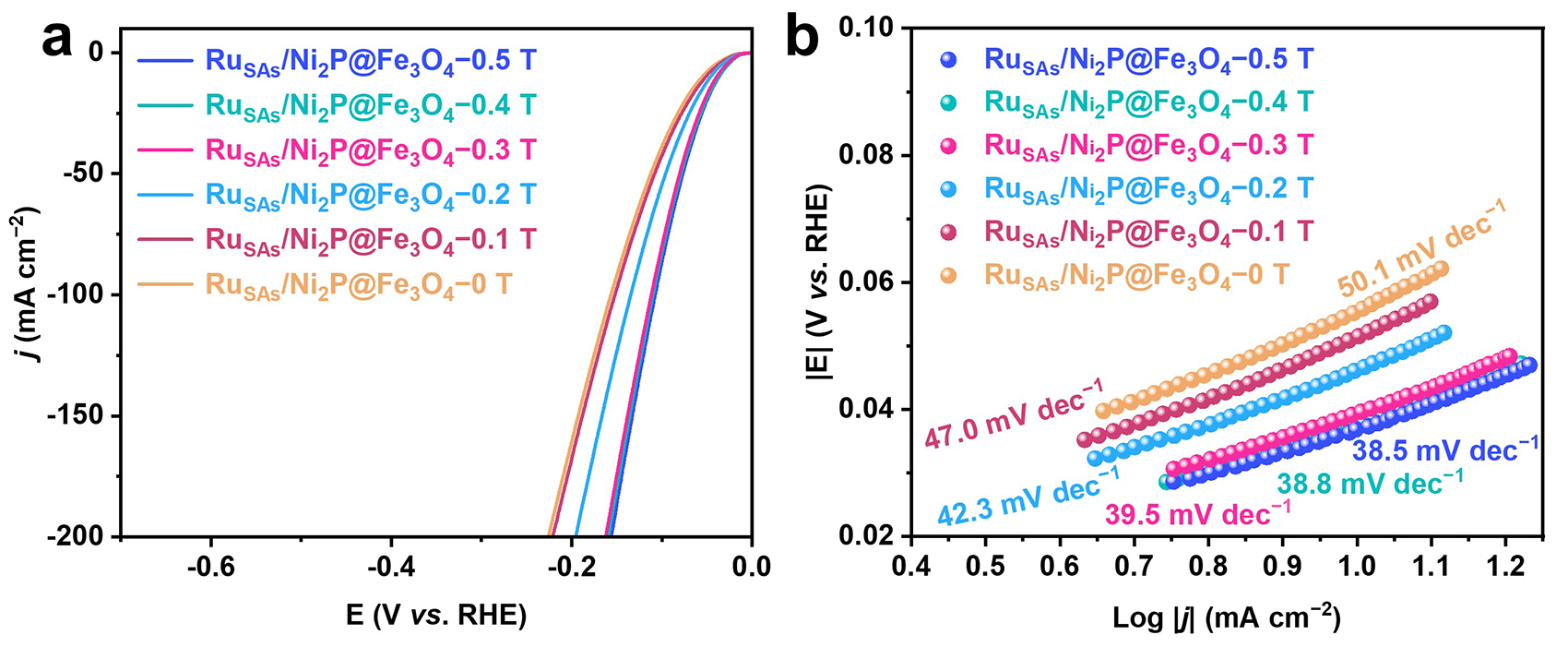


**Figure S23.** At different magnetic field strengths of 0 T, 0.1 T, 0.2 T, 0.3 T, 0.4 T, and 0.5 T, the a) LSV curves, and b) Tafel slopes of the Ru_SAs_/Ni_2_P@Fe_3_O_4_ electrocatalysts.


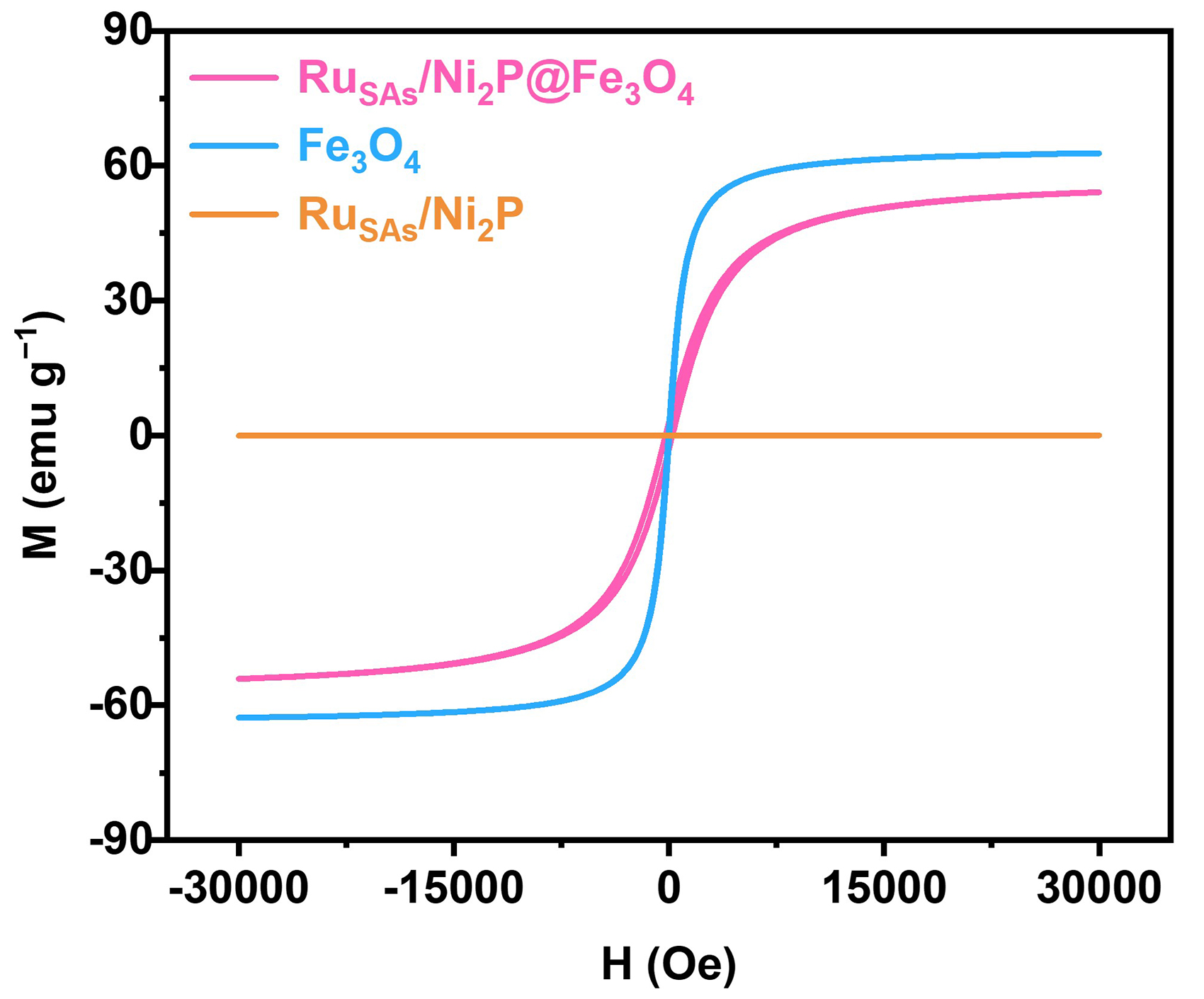


**Figure S24.** Magnetic hysteresis loop of Ru_SAs_/Ni_2_P@Fe_3_O_4_, Fe_3_O_4_, and Ru_SAs_/Ni_2_P.


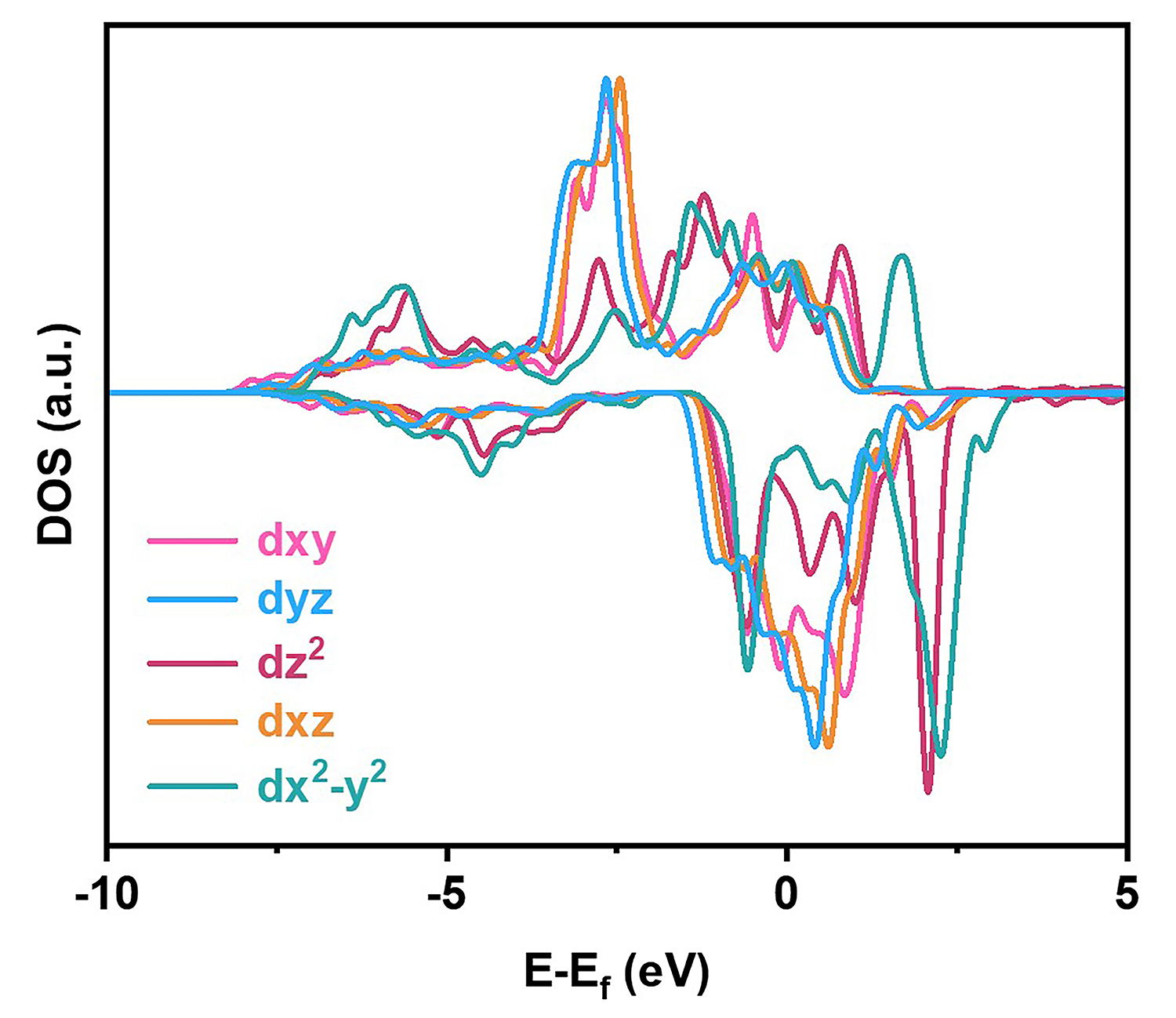


**Figure S25.** Density of states of Fe sites in Fe_3_O_4_.

**Table S5.** Summary of the branching ratio I (L_3_) / [I (L_3_) + I (L_2_)] of Fe element for Ru_SAs_/Ni_2_P@Fe_3_O_4_ and Ru_SAs_/Ni_2_P@Fe_3_O_4_−0.3 T.

| **Catalyst** | **I (L_3_)** | **I (L_2_)** | **I (L_3_) / [I (L_3_) + I (L_2_)]** |
| --- | --- | --- | --- |
| Ru_SAs_/Ni_2_P@Fe_3_O_4_ | 2.420 | 0.709 | 0.774 |
| Ru_SAs_/Ni_2_P@Fe_3_O_4_−0.3 T | 2.562 | 1.999 | 0.778 |

**Table S6.** Summary of the Mössbauer parameters and assignments to different Fe species in Ru_SAs_/Ni_2_P@Fe_3_O_4_.

|  | **IS (mm s****^−1^)** | **QS (mm s^−1^)** | **Γ (mm s^−1^)** | **Content (%)** |
| --- | --- | --- | --- | --- |
| Fe_3_O_4_ (A) | 0.39 | −0.20 | 0.73 | 27.30 |
| Fe_3_O_4_ (B) | 0.80 | 0.05 | 1.03 | 54.10 |
| Fe^2+^ (LS) | 0.68 | 2.37 | 0.57 | 6.30 |
| Fe^3+^ (LS) | 0.18 | 0.39 | 0.32 | 2.40 |
| Fe^2+^ (HS) | 1.39 | 2.31 | 0.67 | 8.70 |
| Fe^3+^ (HS) | 0.37 | 0.42 | 0.27 | 1.20 |

**Table S7.** Summary of the Mössbauer parameters and assignments to different Fe species in Ru_SAs_/Ni_2_P@Fe_3_O_4_−0.3 T.

|  | **IS (mm s^−1^)** | **QS (mm s^−1^)** | **Γ (mm s^−1^)** | **Content (%)** |
| --- | --- | --- | --- | --- |
| Fe_3_O_4_ (A) | 0.34 | −0.20 | 0.73 | 26.70 |
| Fe_3_O_4_ (B) | 0.86 | 0.05 | 1.04 | 53.70 |
| Fe^2+^ (LS) | 0.69 | 2.39 | 0.48 | 6.40 |
| Fe^3+^ (LS) | 0.10 | 0.40 | 0.22 | 1.30 |
| Fe^2+^ (HS) | 1.37 | 2.43 | 0.66 | 8.80 |
| Fe^3+^ (HS) | 0.35 | 0.38 | 0.33 | 3.10 |


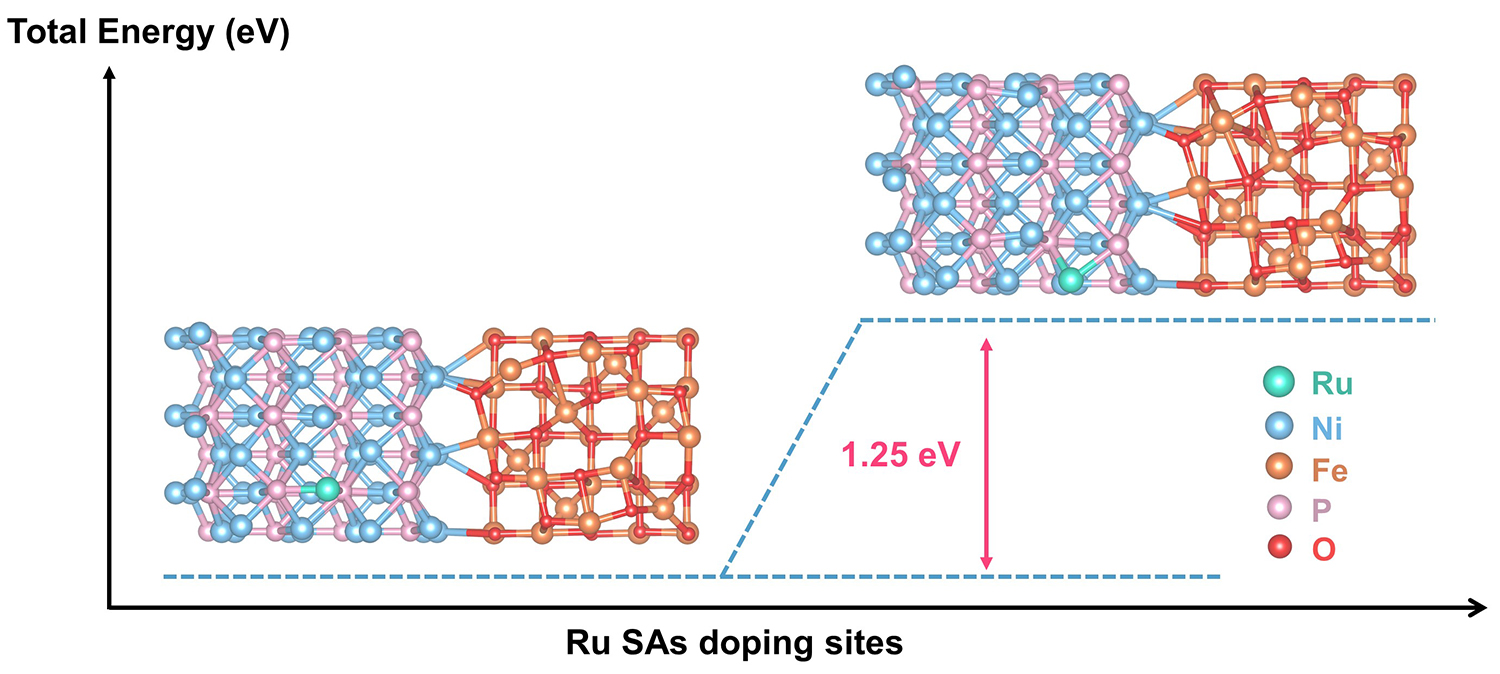


**Figure S26****.** Schematic diagram of energy comparison of systems with Ru SAs doping at different sites of Ni_2_P@Fe_3_O_4_. Among them, the structure with the lower energy value is in a relatively stable state.


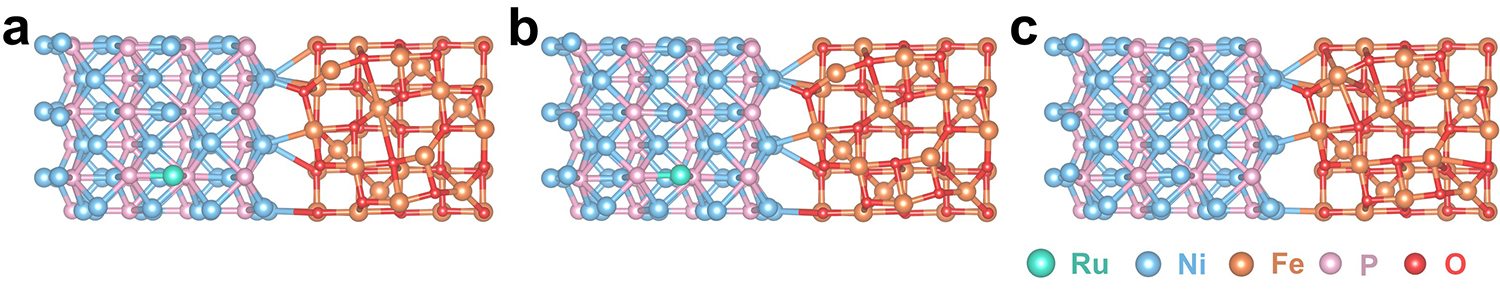


**Figure S27.** Crystal structure models of a) Ru_SAs_/Ni_2_P@Fe_3_O_4_−0.3 T, b) Ru_SAs_/Ni_2_P@Fe_3_O_4_, and c) Ni_2_P@Fe_3_O_4_.


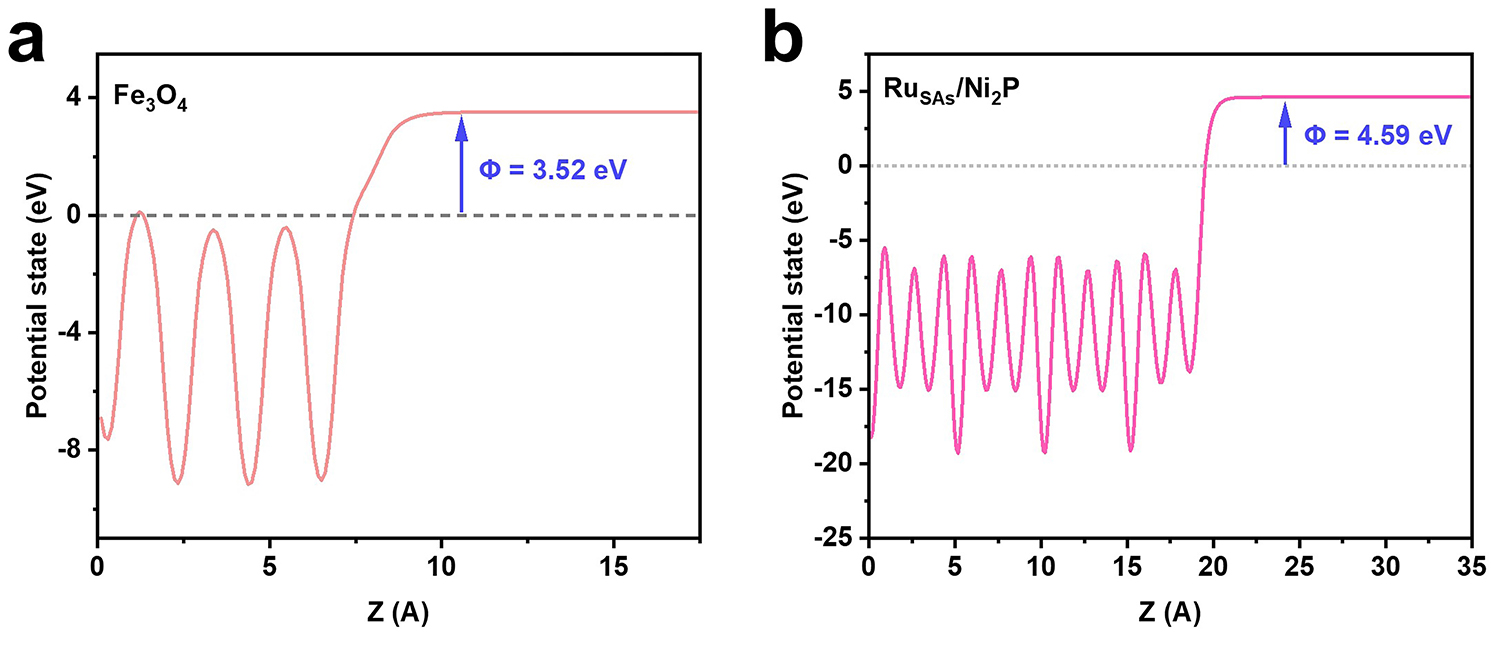


**Figure S28.** Work functions of a) Fe_3_O_4_ and b) Ru_SAs_/Ni_2_P from electrostatic potential calculations.


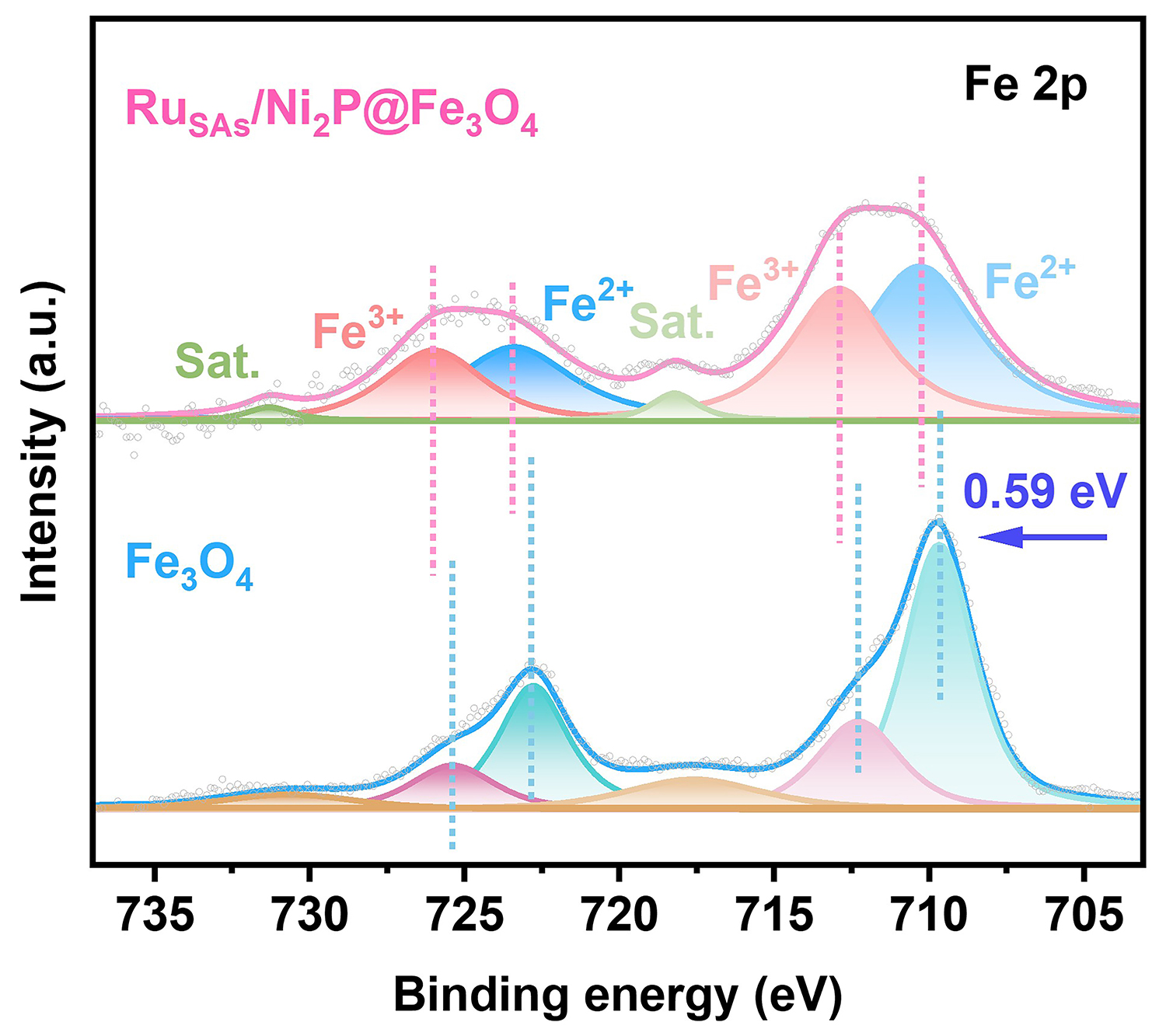


**Figure S29.** High-resolution Fe 2p spectra of Ru_SAs_/Ni_2_P@Fe_3_O_4_ and Fe_3_O_4_.


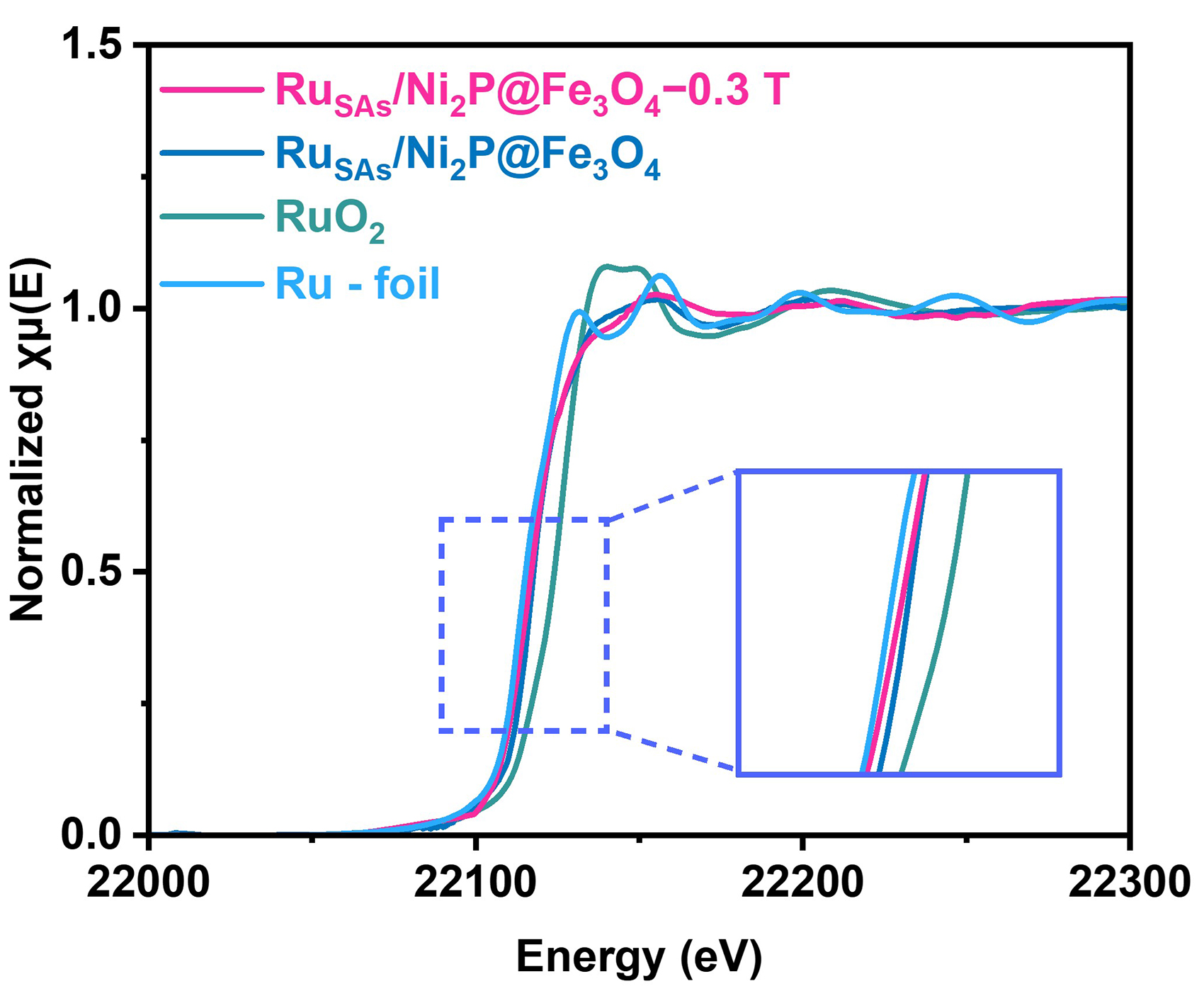


**Figure S30.** Ru K-edge XANES spectra of Ru_SAs_/Ni_2_P@Fe_3_O_4_−0.3 T, RuO_2_, and Ru foil.


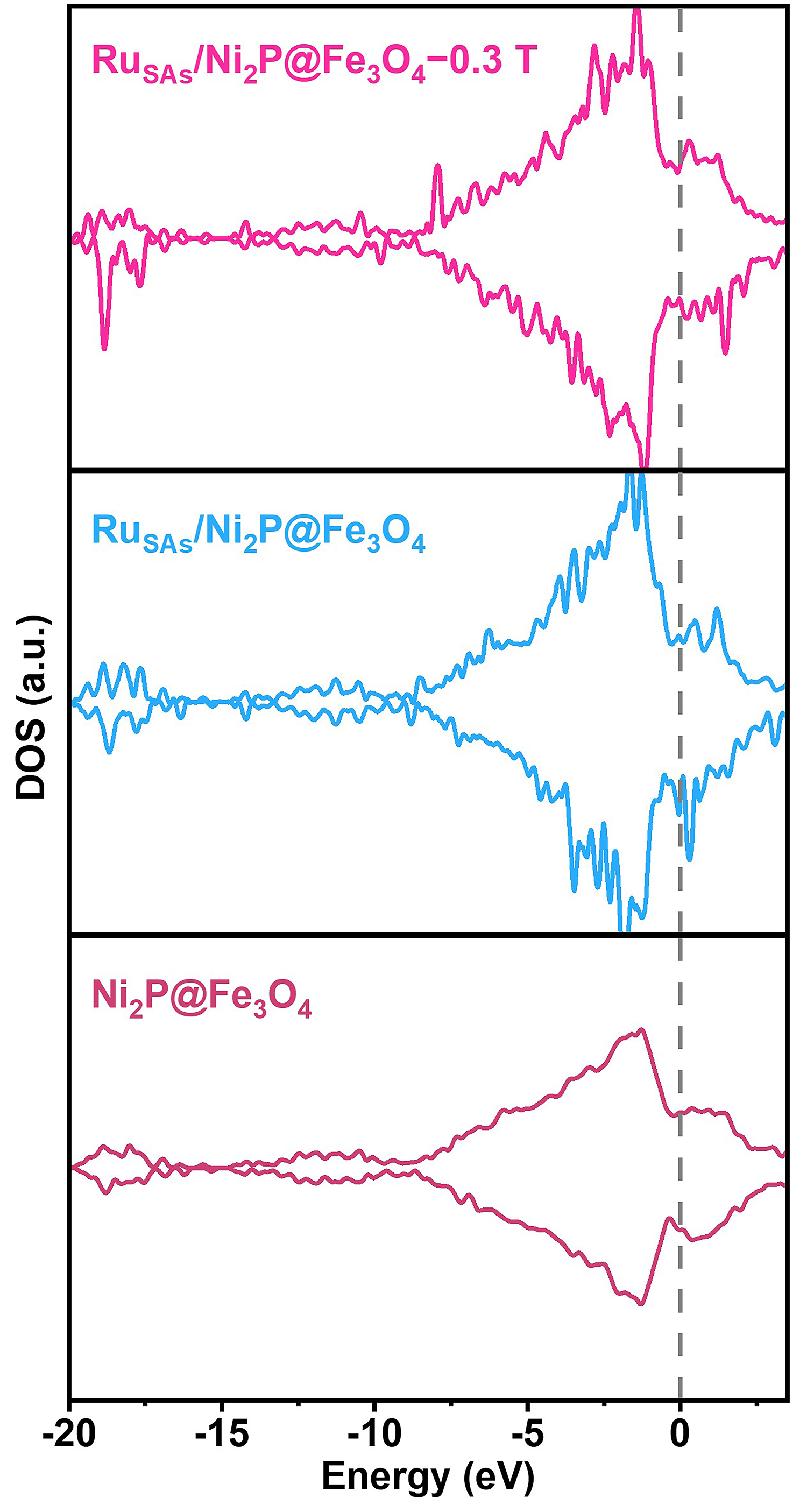


**Figure S31.** DOS spectra of Ru_SAs_/Ni_2_P@Fe_3_O_4_−0.3 T, Ru_SAs_/Ni_2_P@Fe_3_O_4_, and Ni_2_P@Fe_3_O_4_.

**Table S8.** Water dissociation of Ni_2_P@Fe_3_O_4_ on different sites.

| **slab** | **slab_H2O*_** | **TS (eV)** | **slab_(H-OH)*_** |
| --- | --- | --- | --- |
| Ni_2_P@Fe_3_O_4_ | 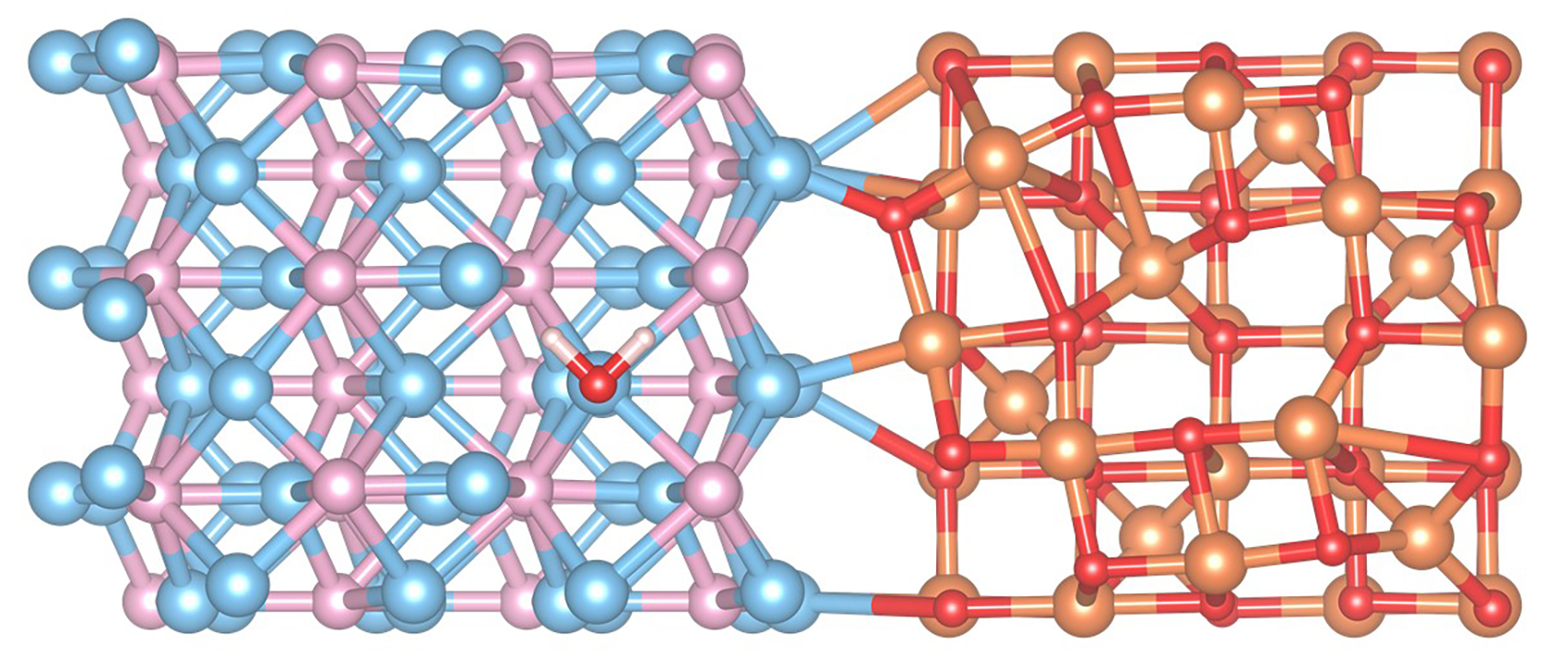 | 0.64 | 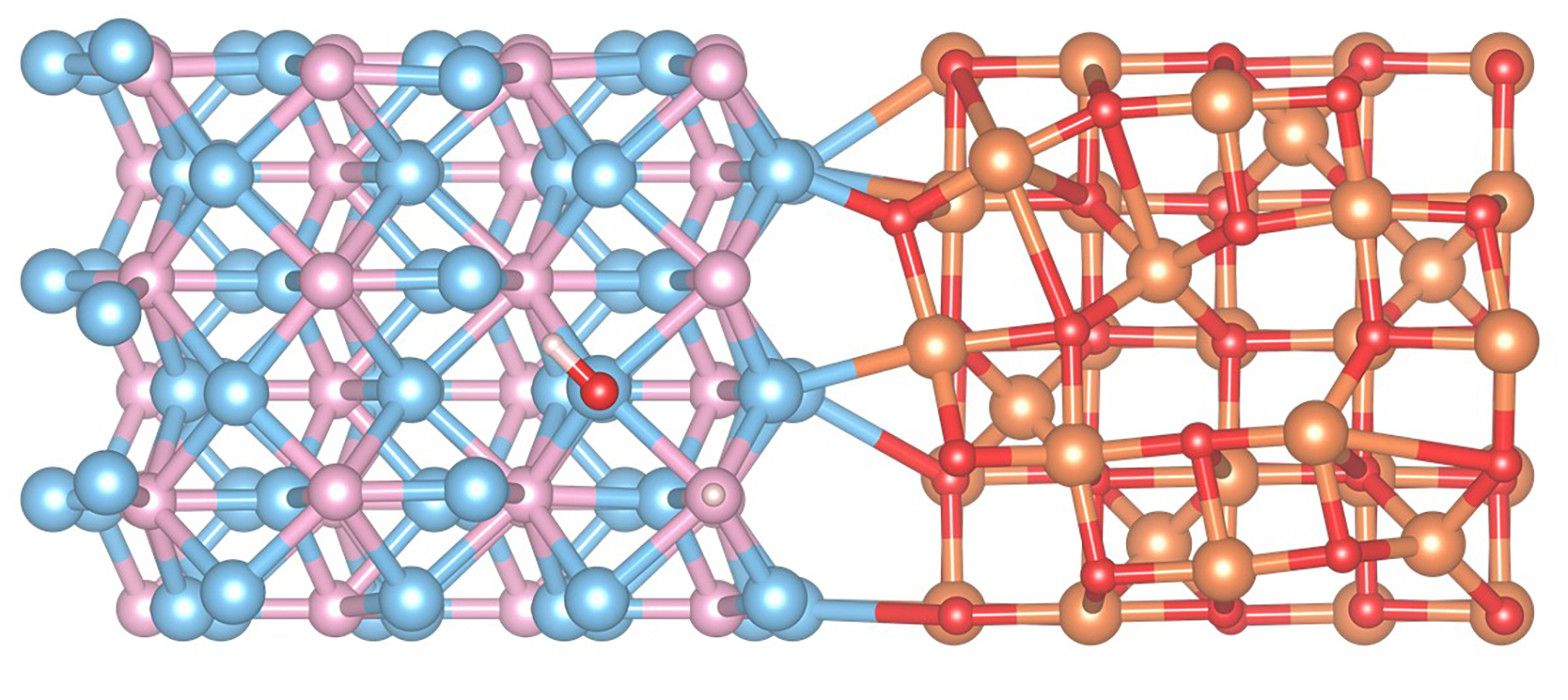 |
|  | 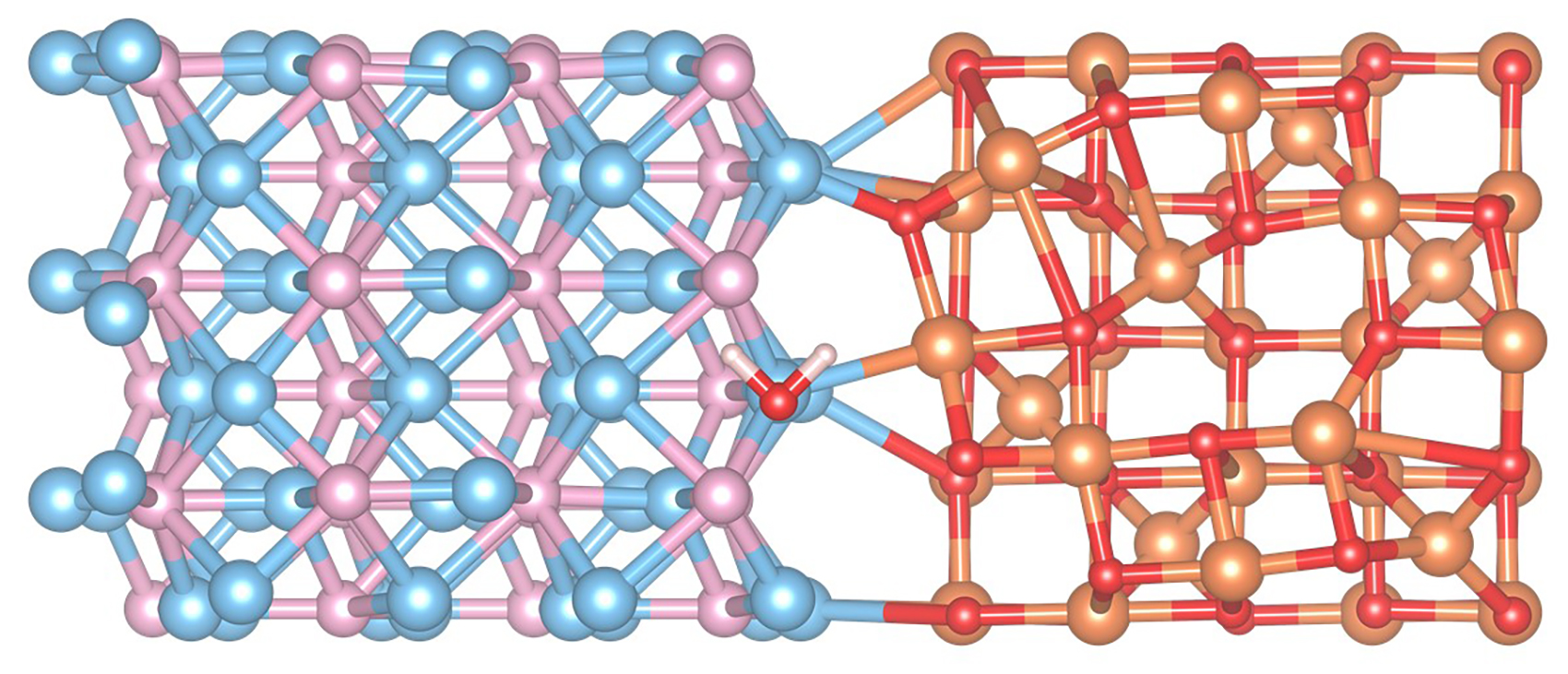 | 0.48 | 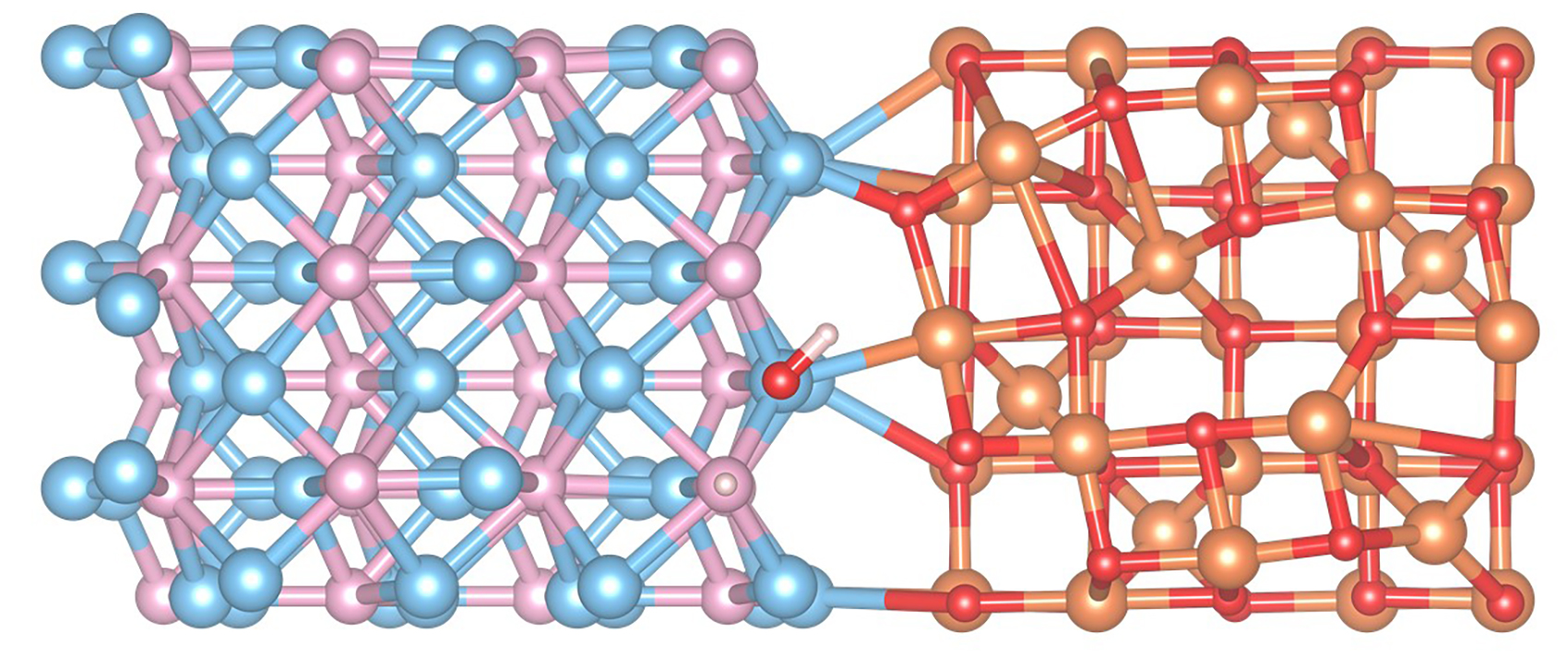 |
|  | 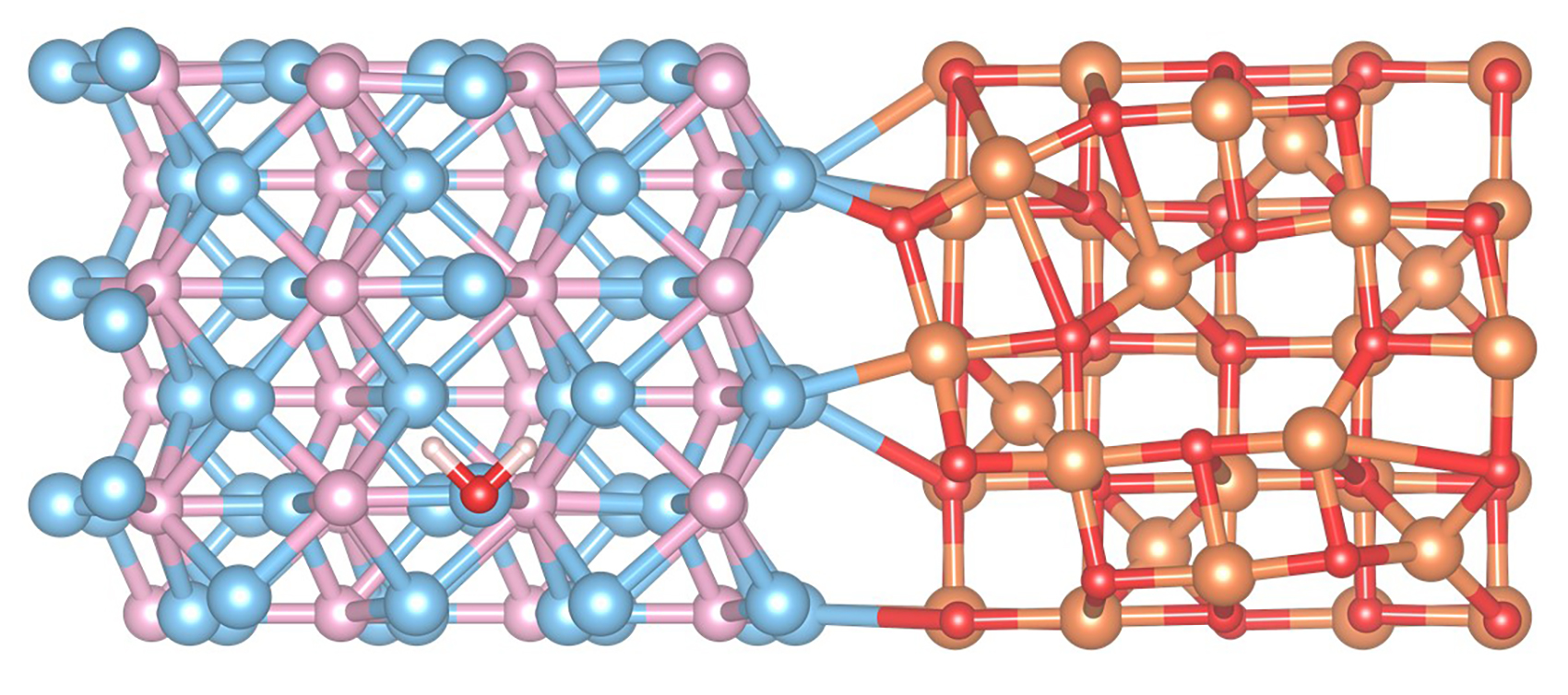 | 0.56 | 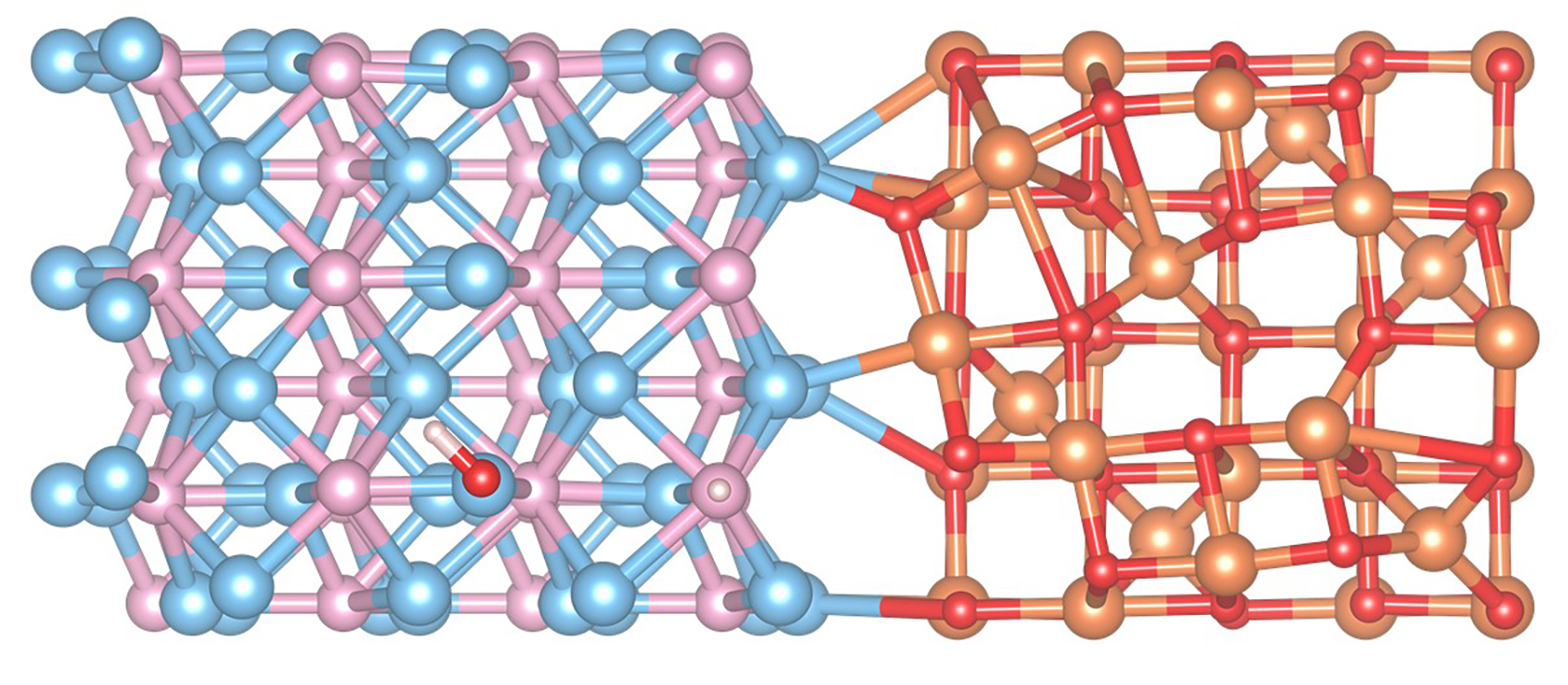 |
|  | 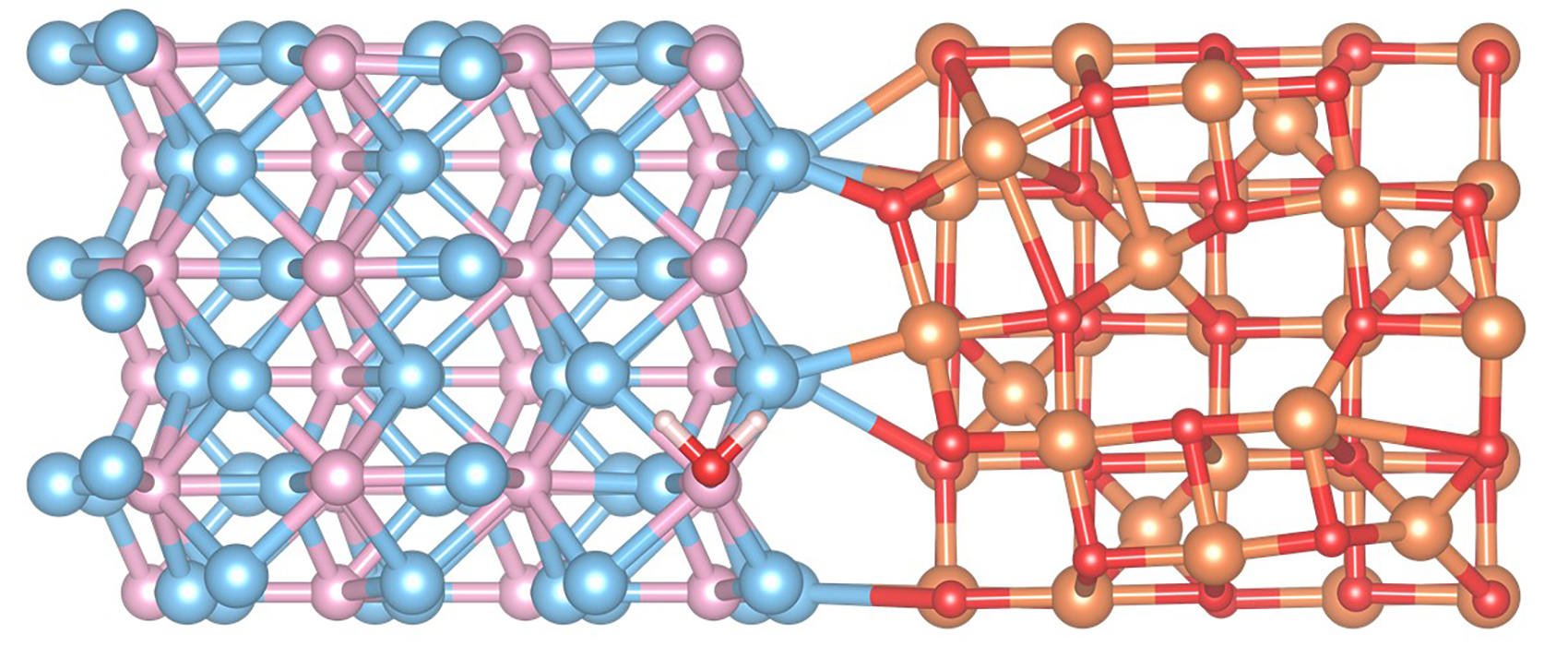 | 0.50 | 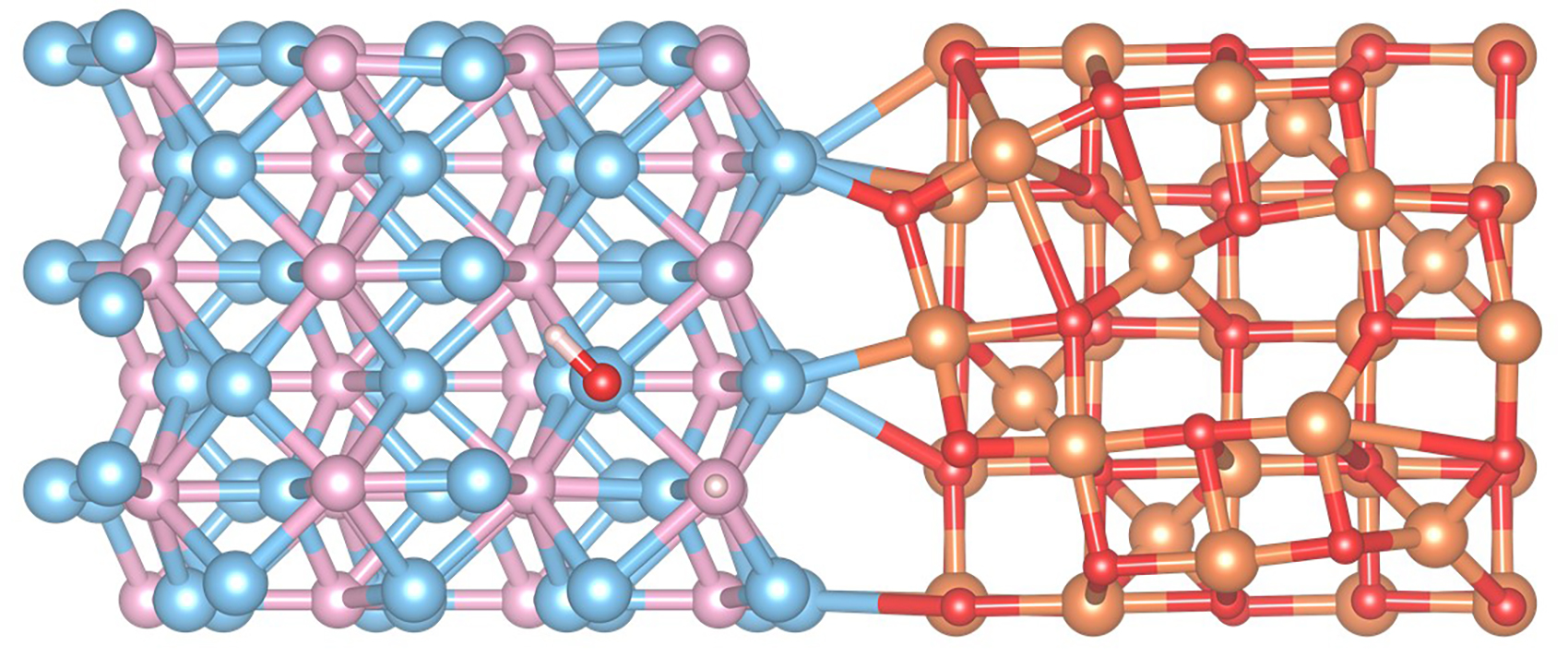 |


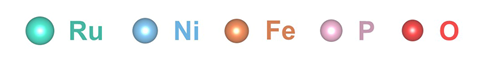


**Table S9.** Water dissociation of Ru_SAs_/Ni_2_P@Fe_3_O_4_ on different sites.

| **slab** | **slab_H_****_2O*_** | **TS (eV)** | **slab_(H-OH)*_** |
| --- | --- | --- | --- |
| Ru_SAs_/Ni_2_P@Fe_3_O_4_ | 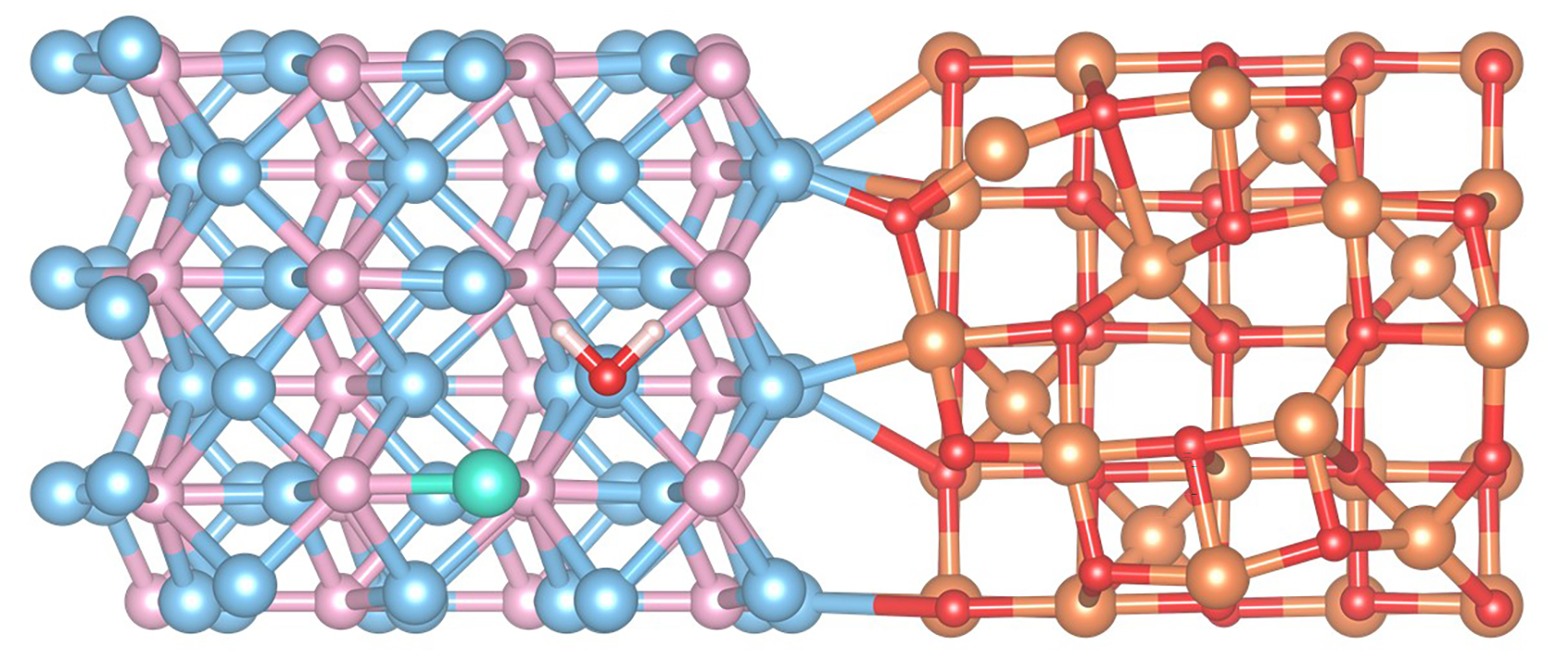 | 0.61 | 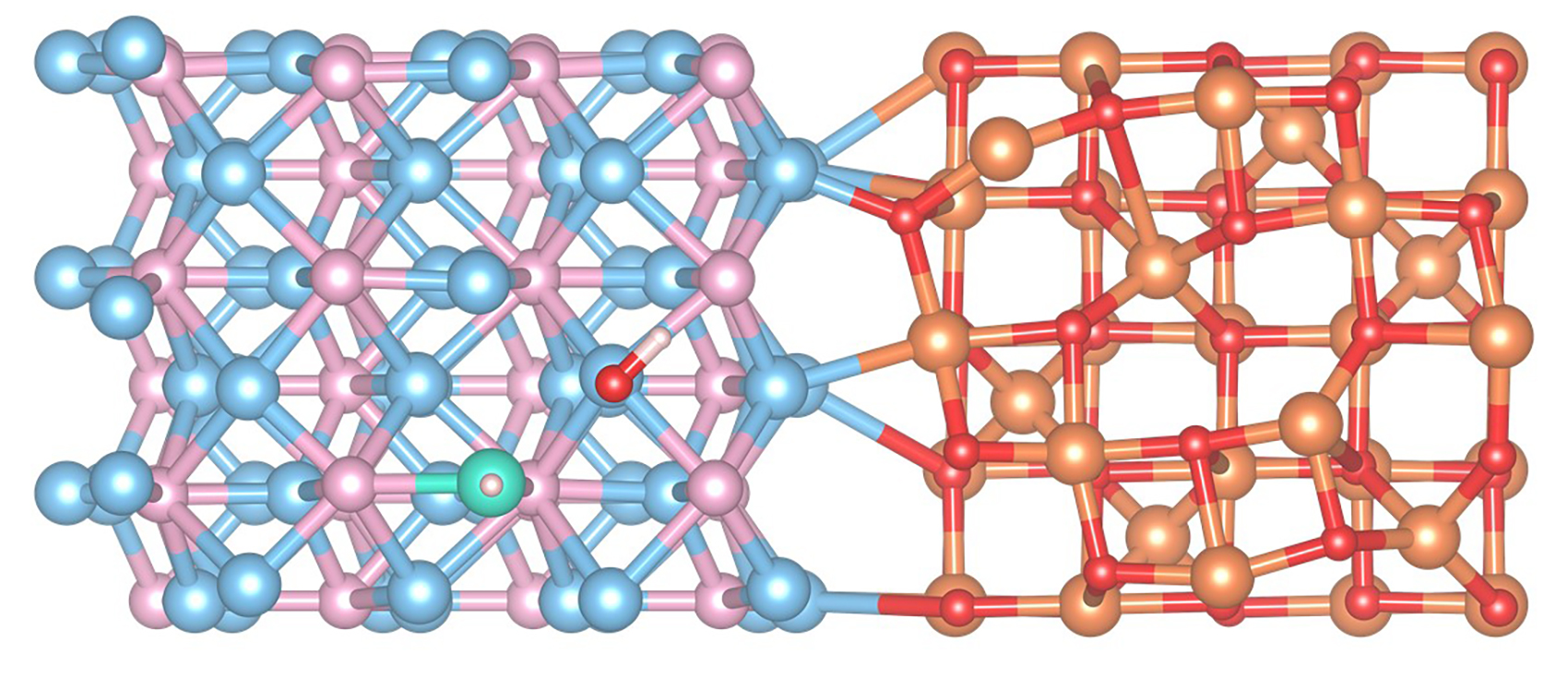 |
|  | 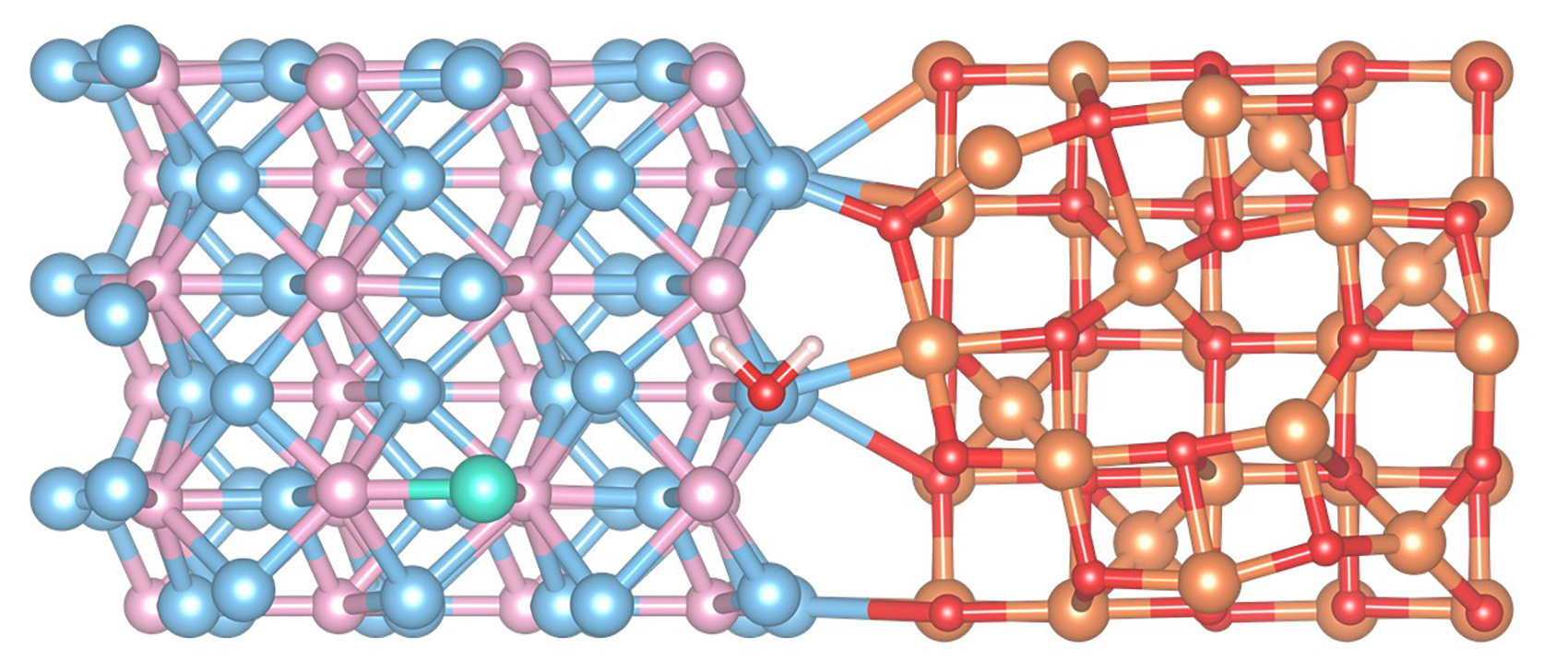 | 0.54 | 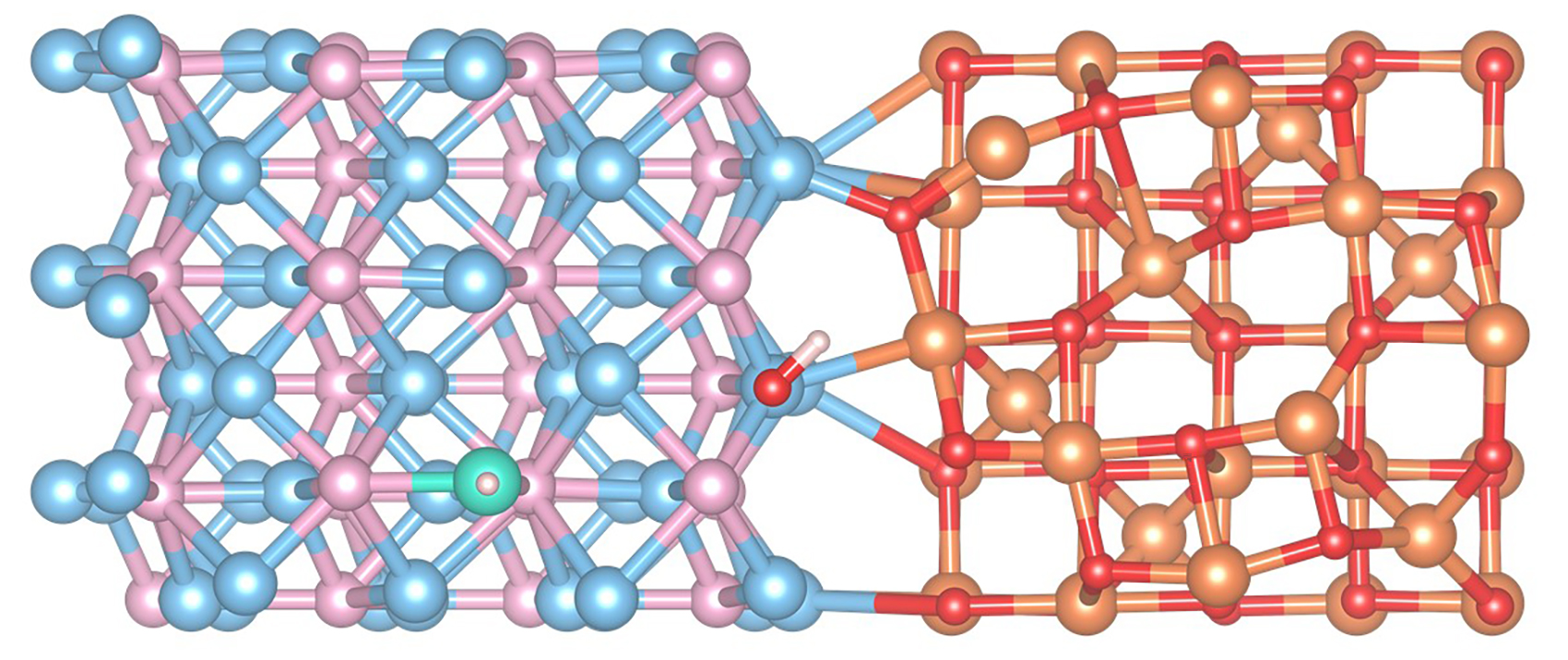 |
|  | 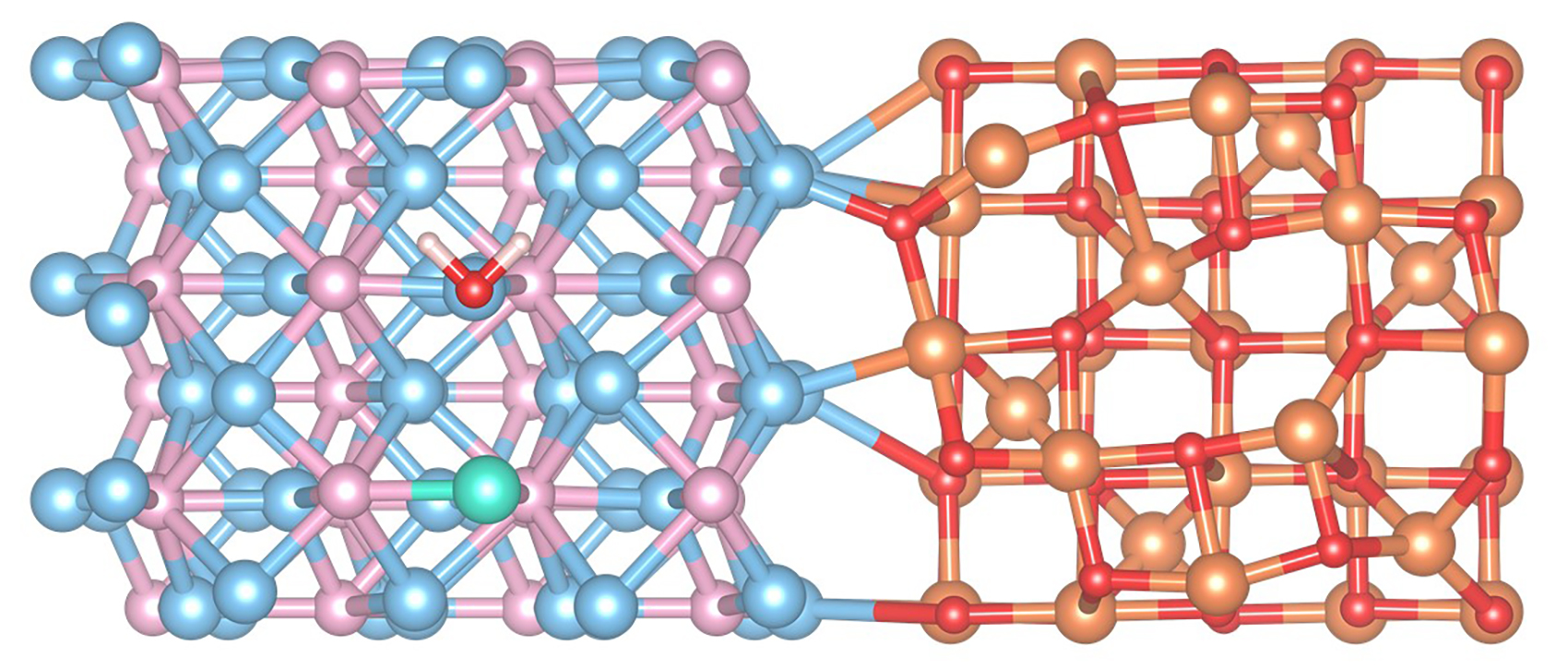 | 0.42 | 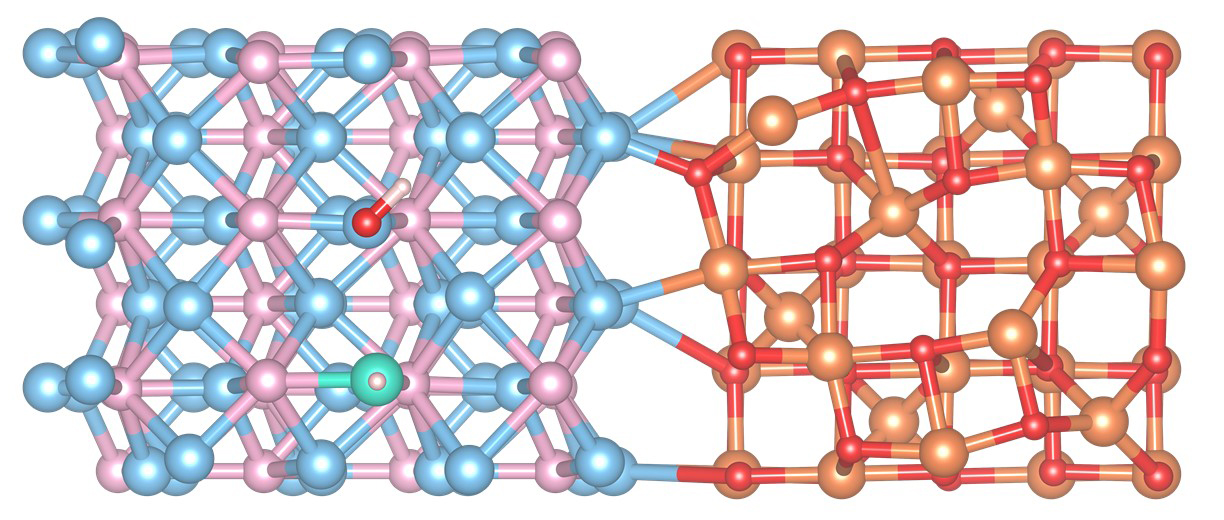 |
|  | 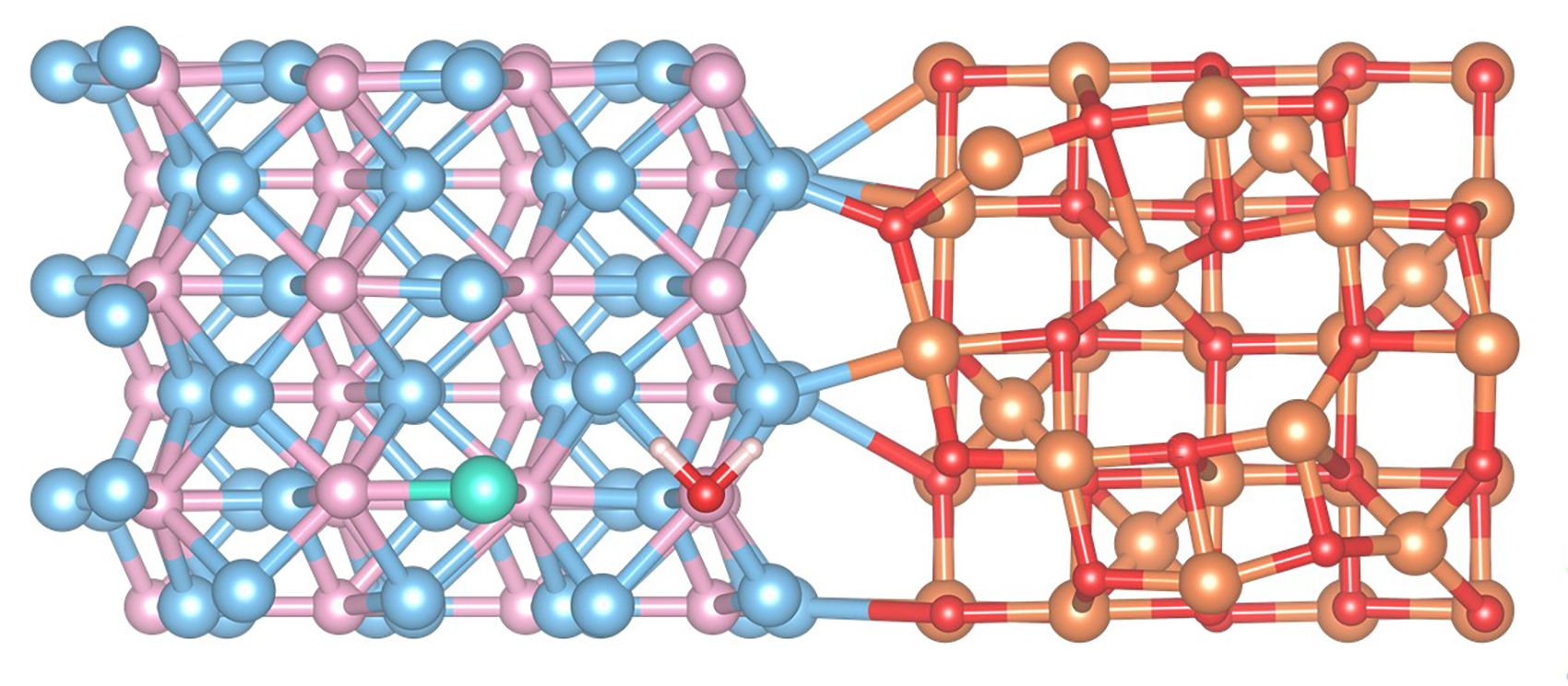 | 0.39 | 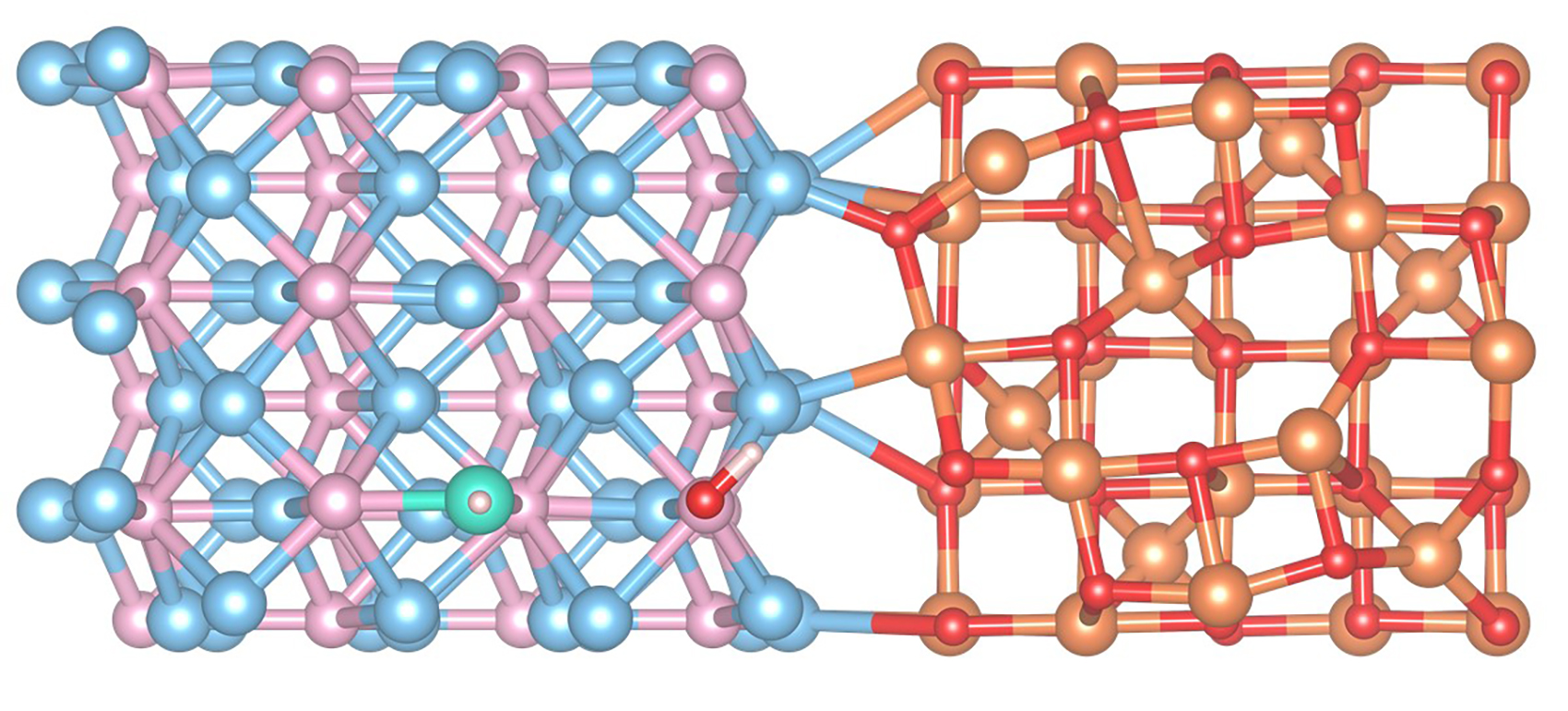 |
|  | 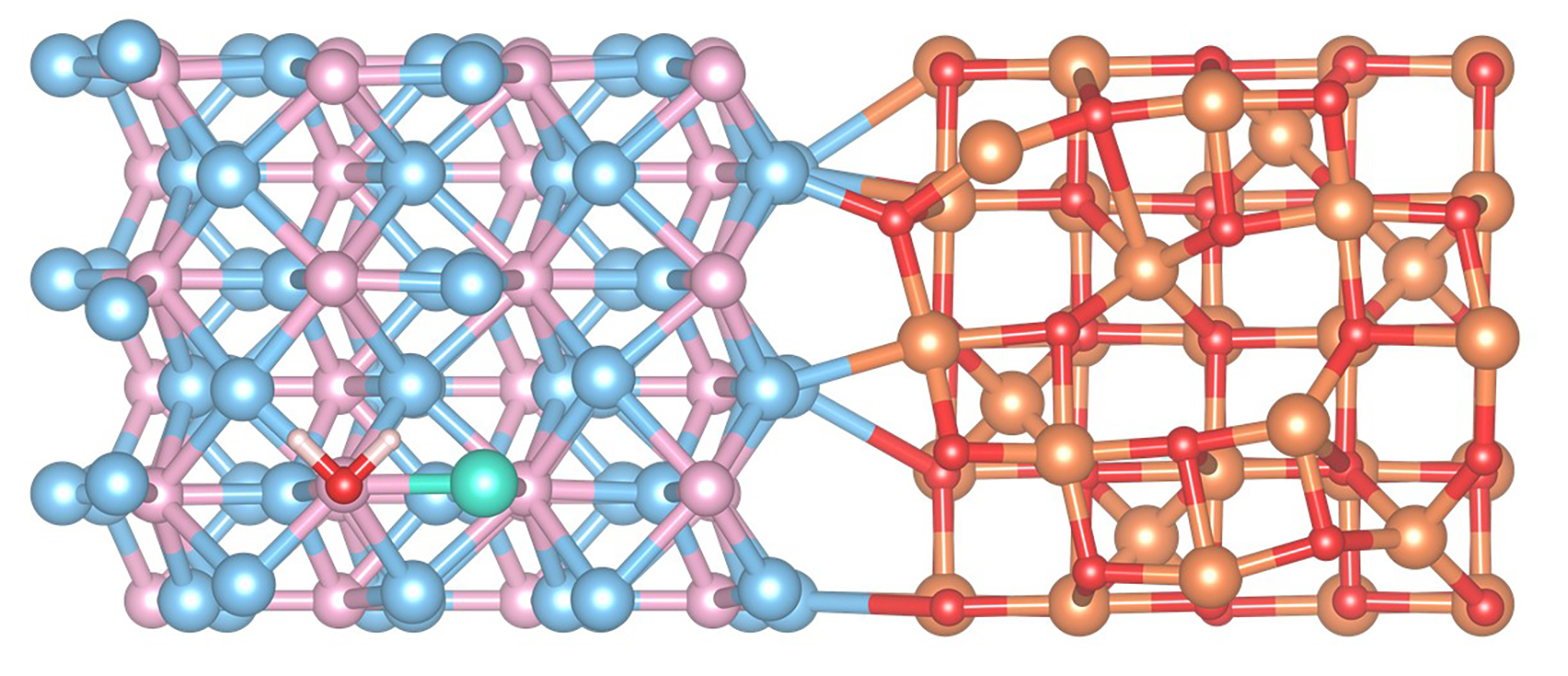 | 0.65 | 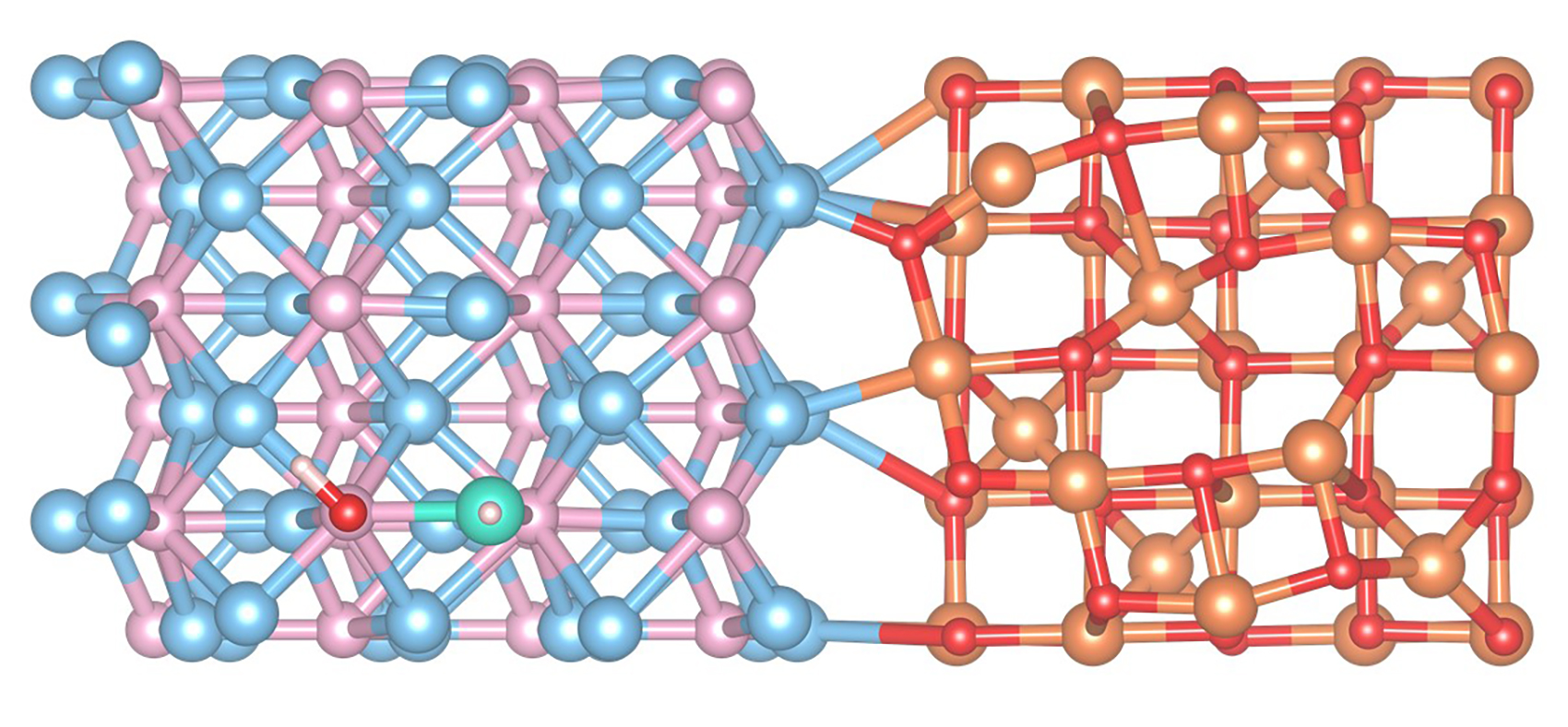 |
|  | 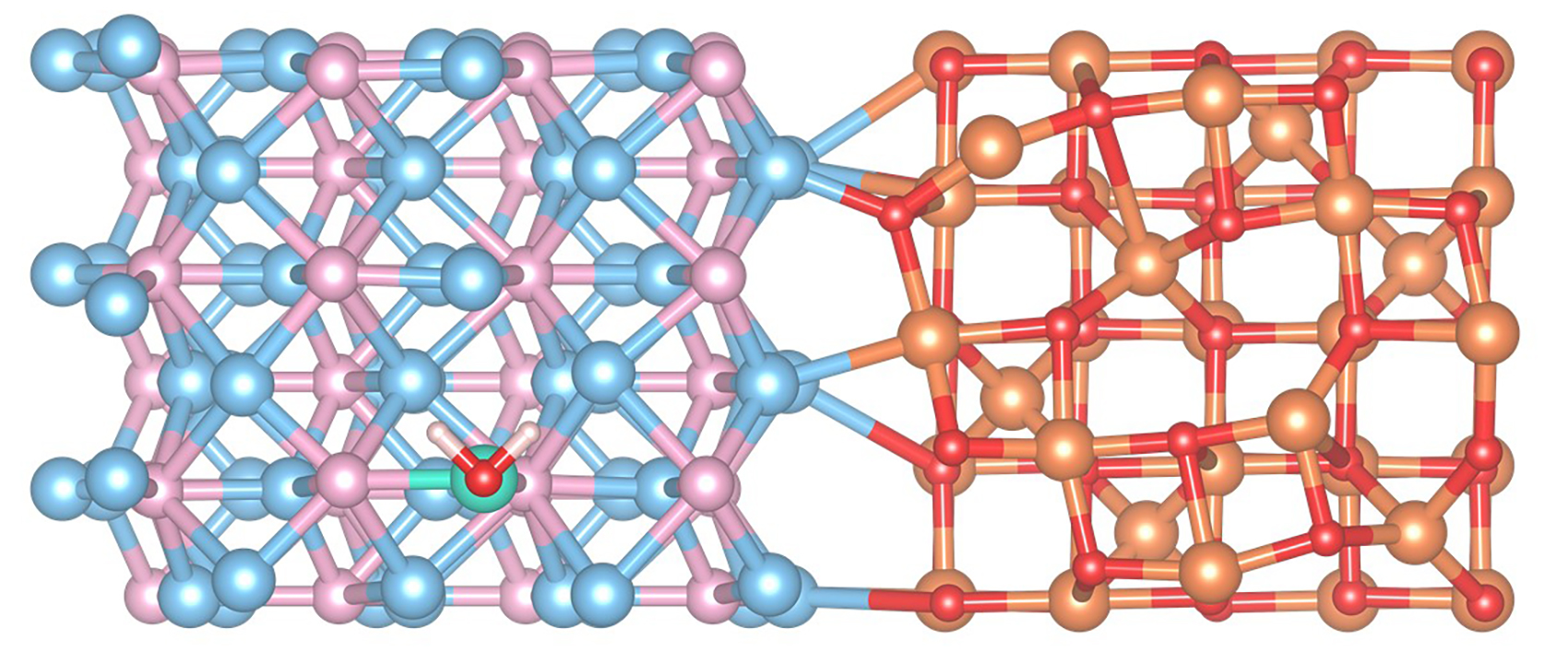 | 0.37 | 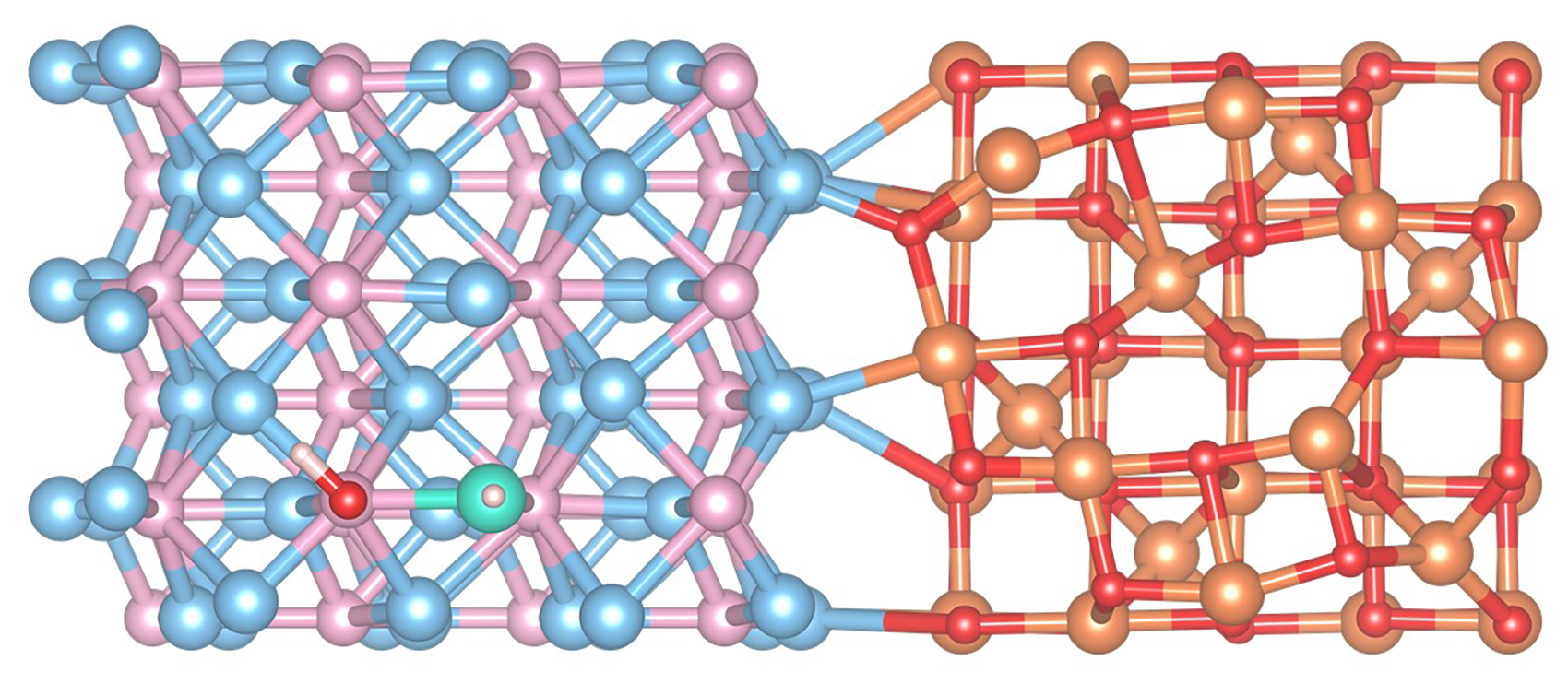 |


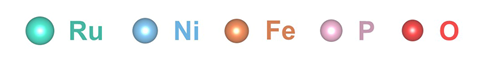


**Table S10.** Water dissociation of Ru_SAs_/Ni_2_P@Fe_3_O_4_−0.3 T on different sites.

| **slab** | **slab_H2O*_** | **TS (eV)** | **slab_(H-OH)*_** |
| --- | --- | --- | --- |
| Ru_SAs_/Ni_2_P@Fe_3_O_4_−0.3 T | 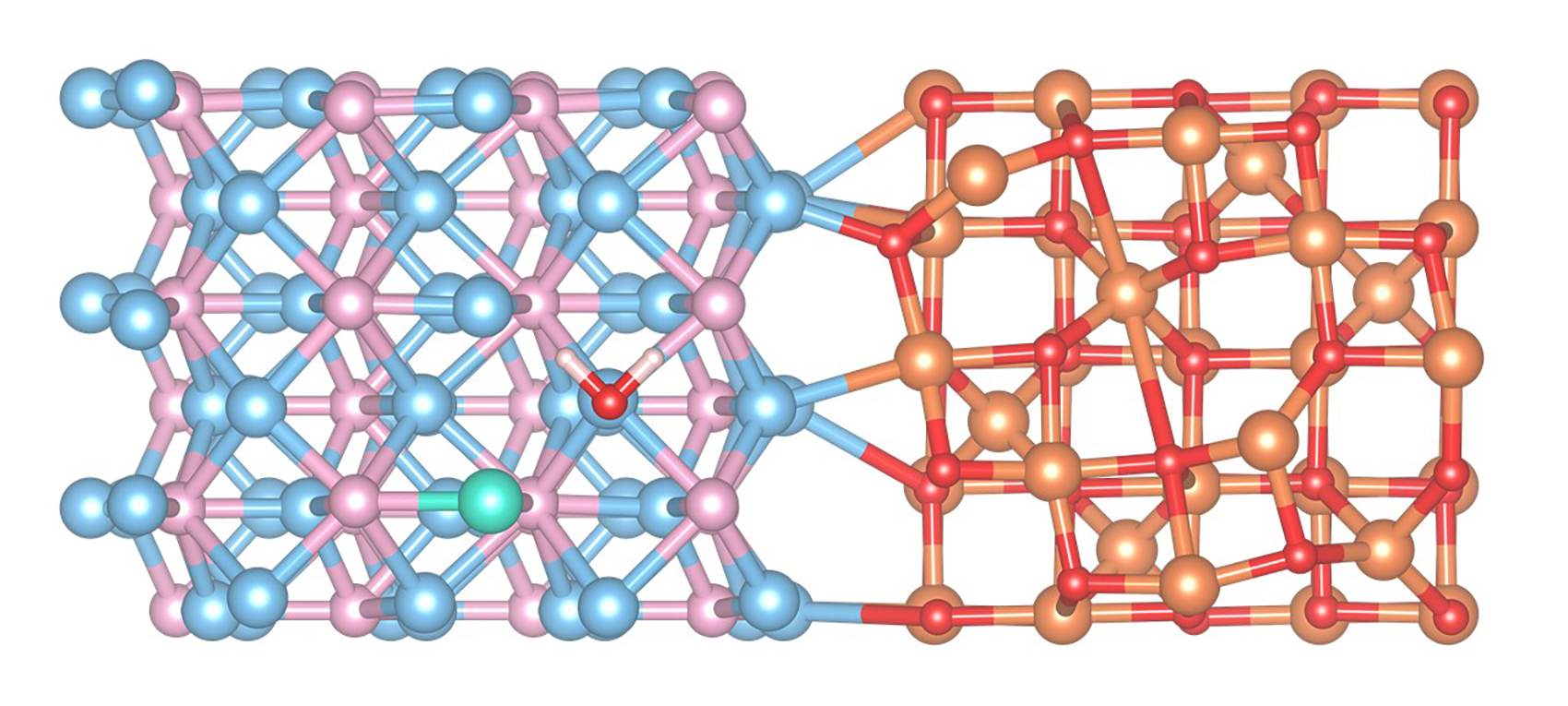 | 0.42 | 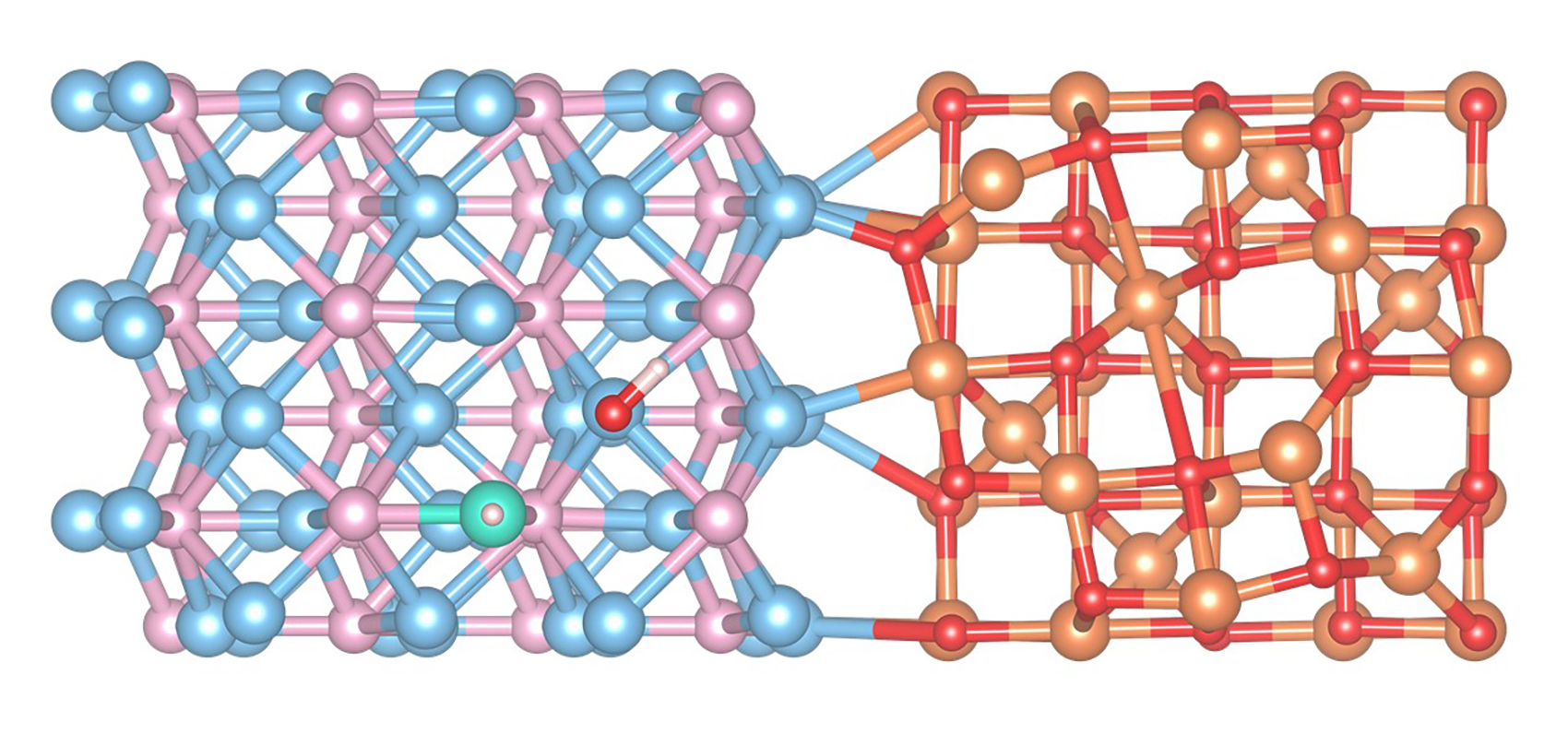 |
|  | 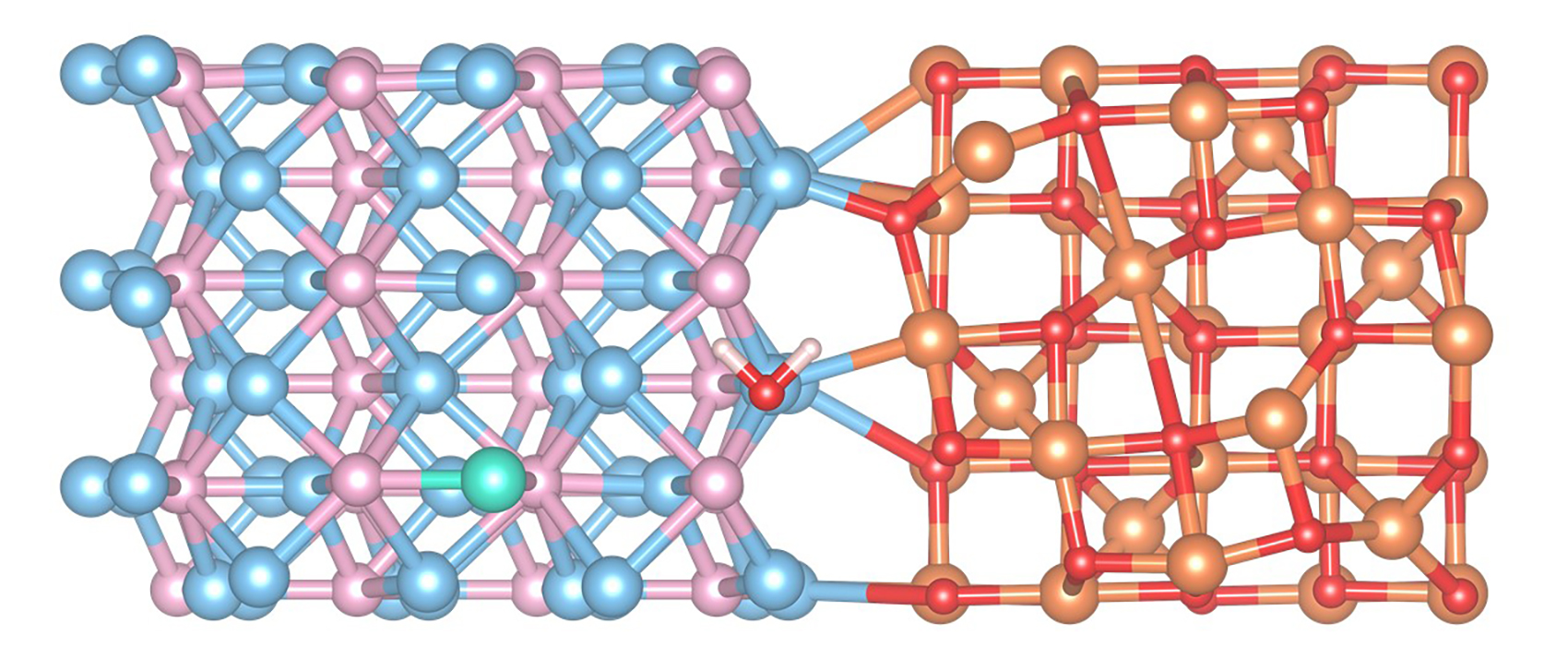 | 0.35 | 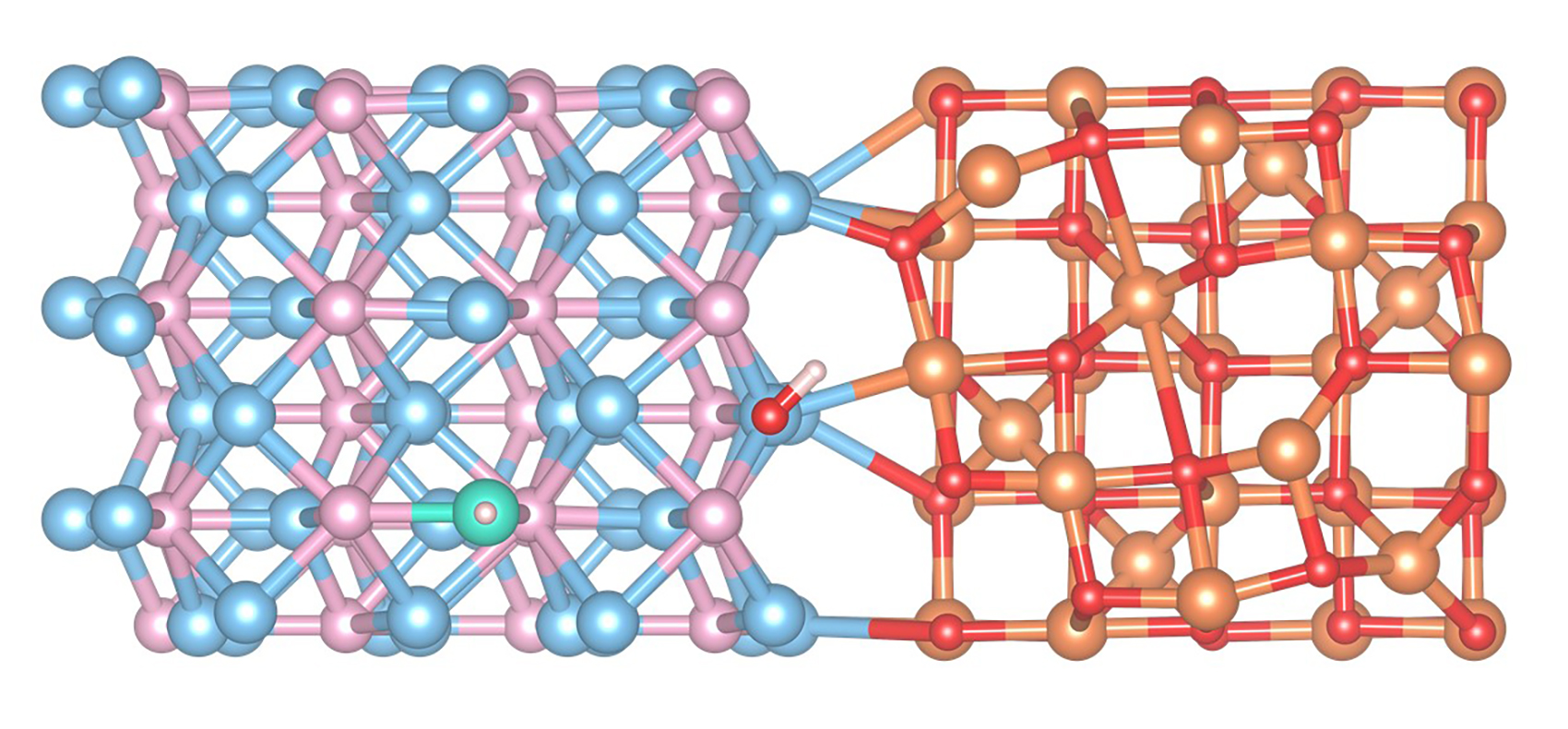 |
|  | 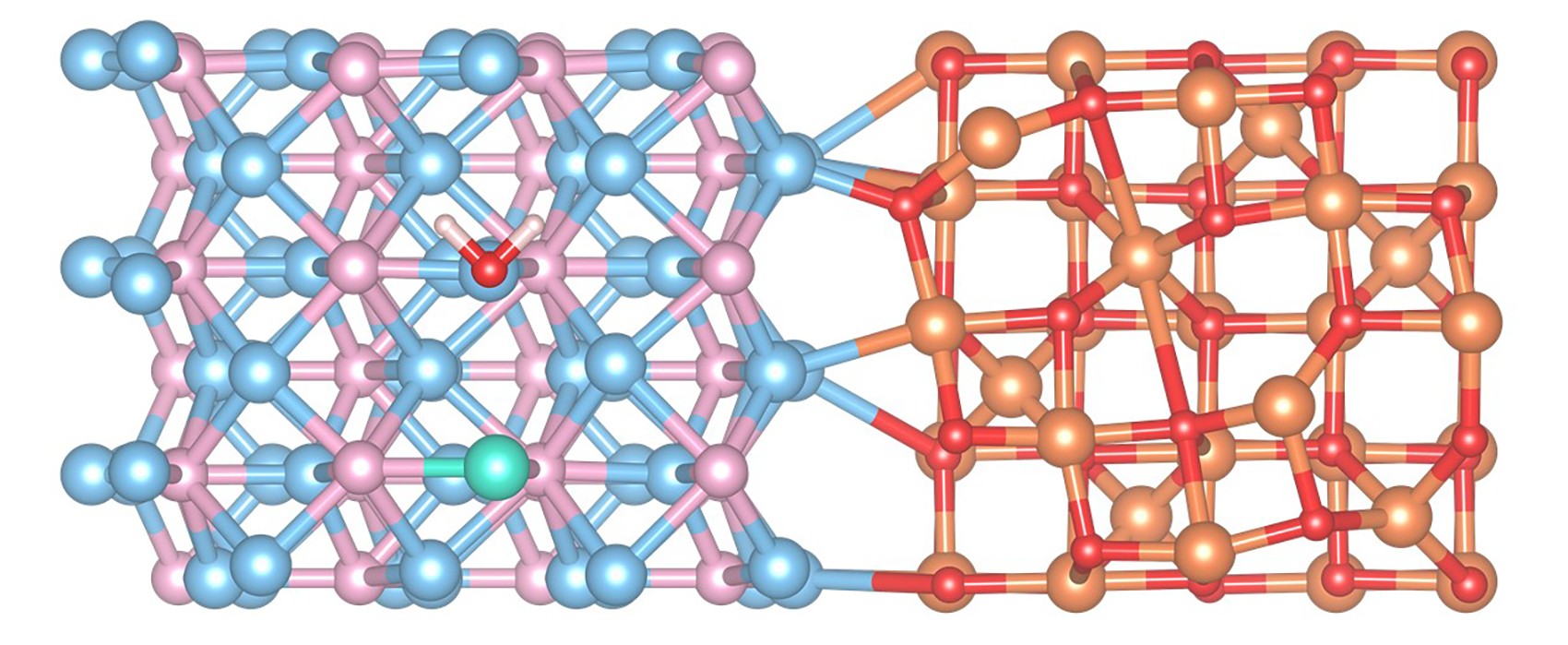 | 0.74 | 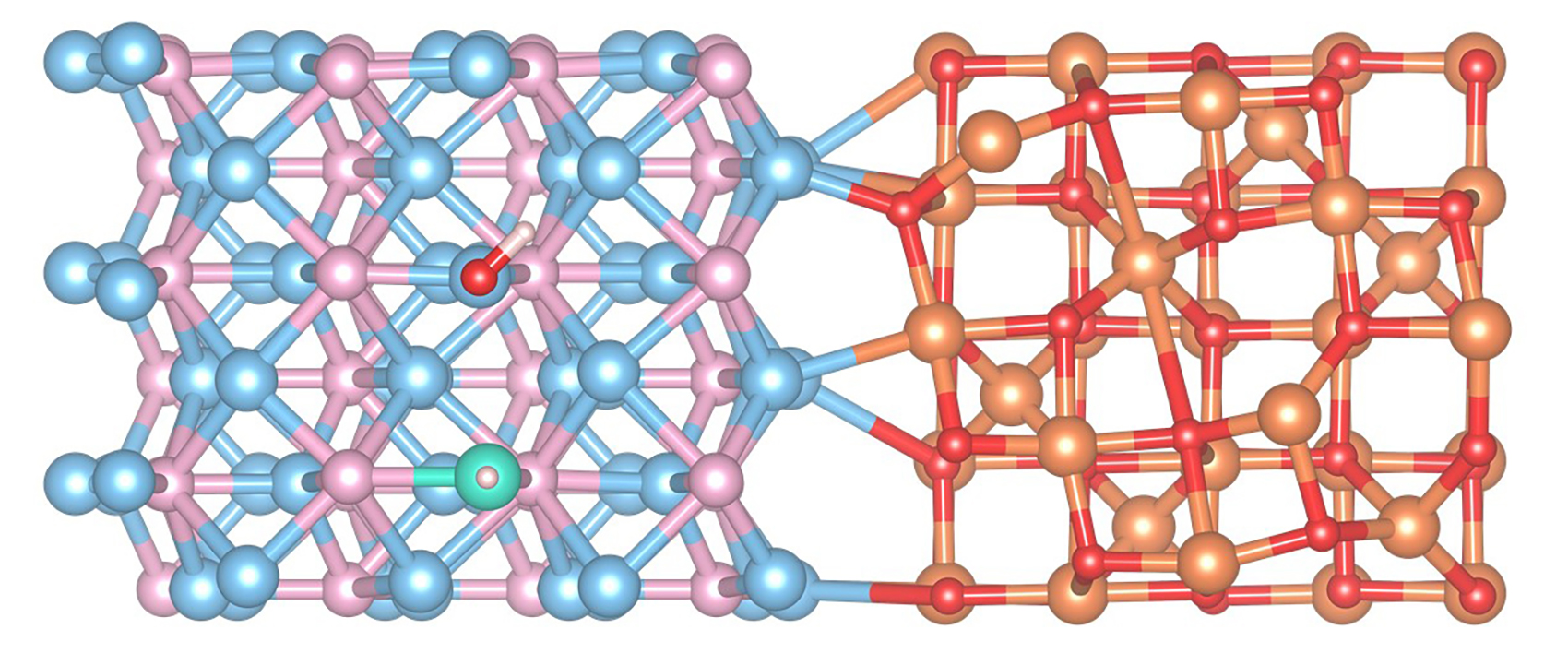 |
|  | 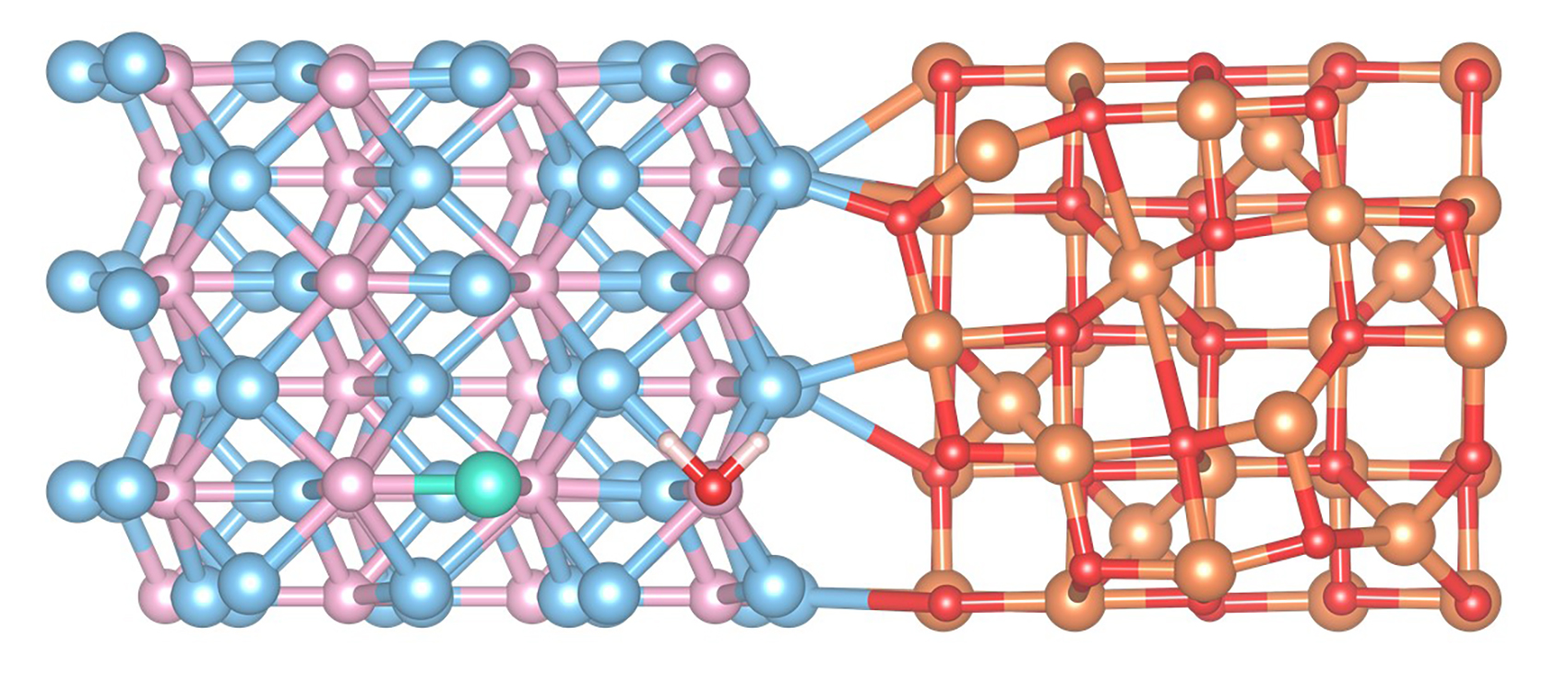 | 0.43 | 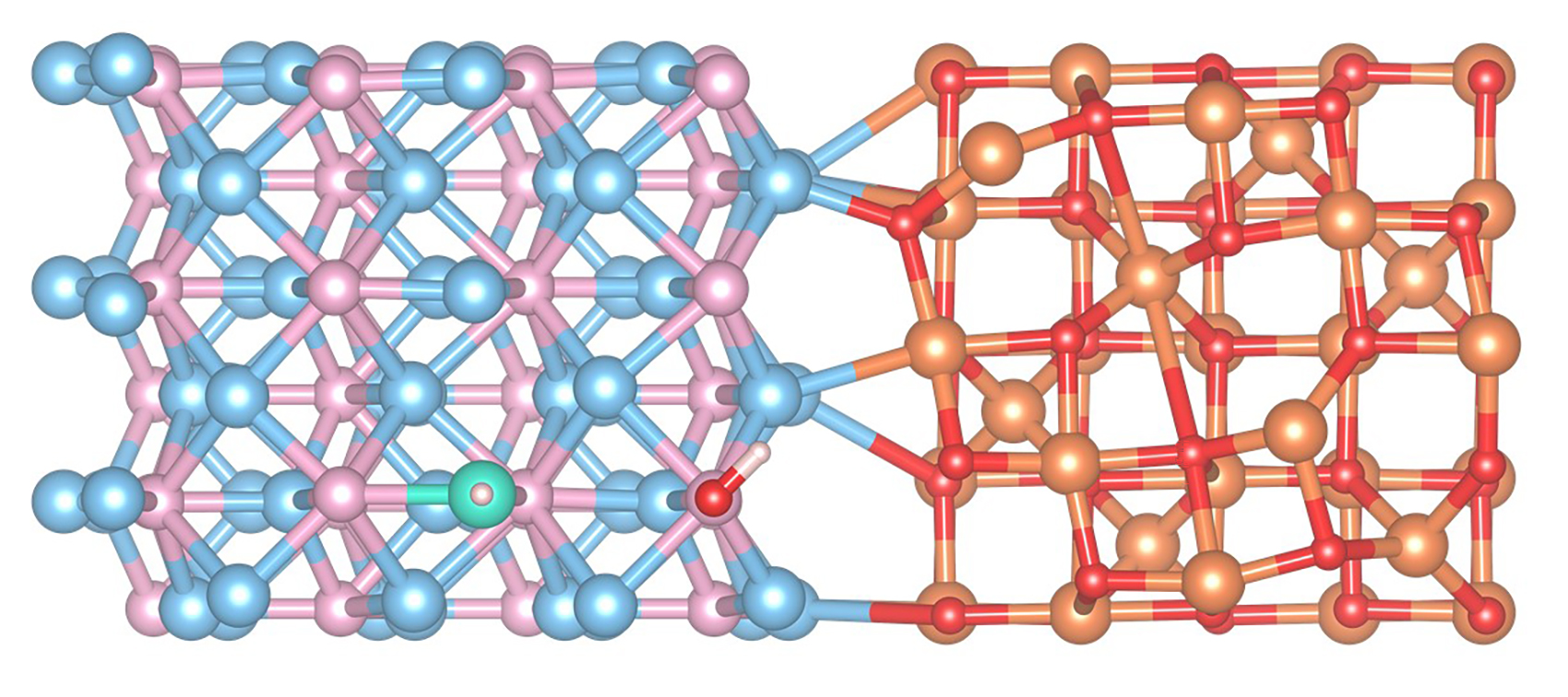 |
|  | 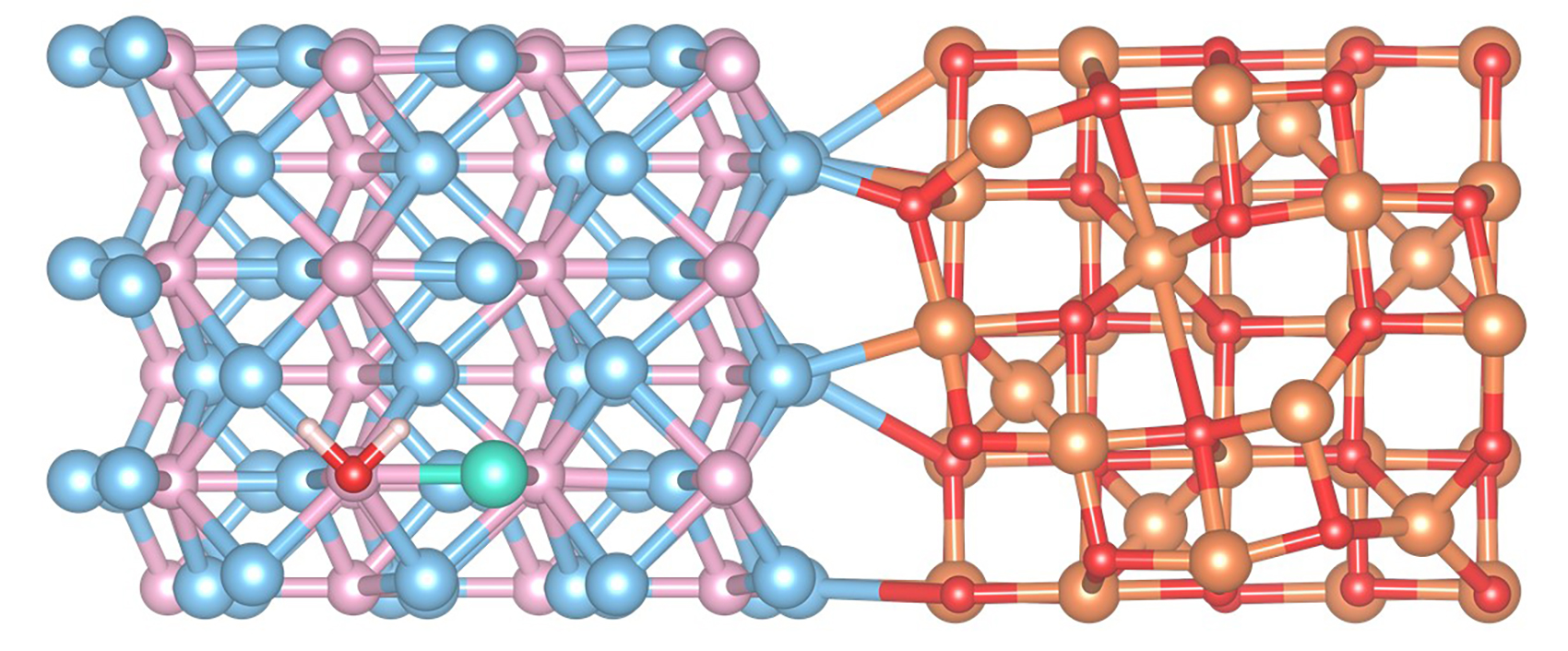 | 0.58 | 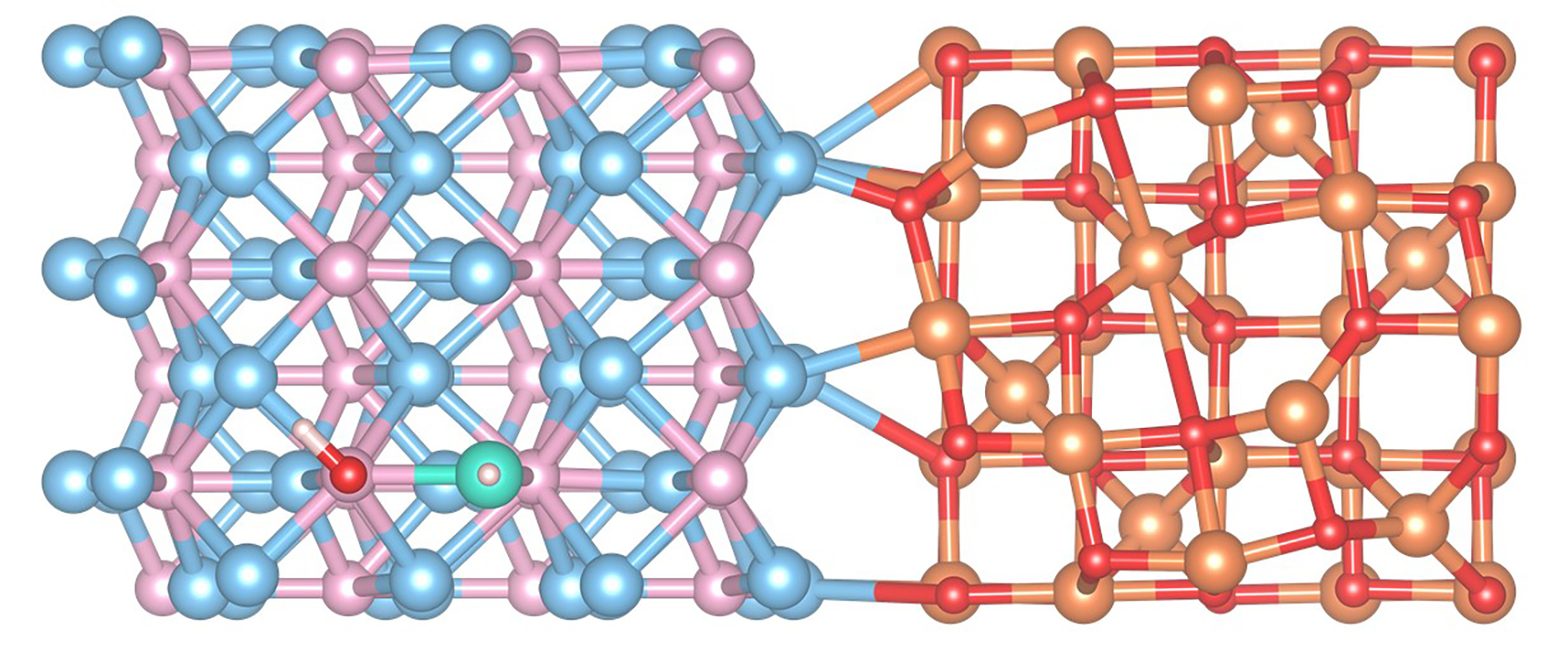 |
|  | 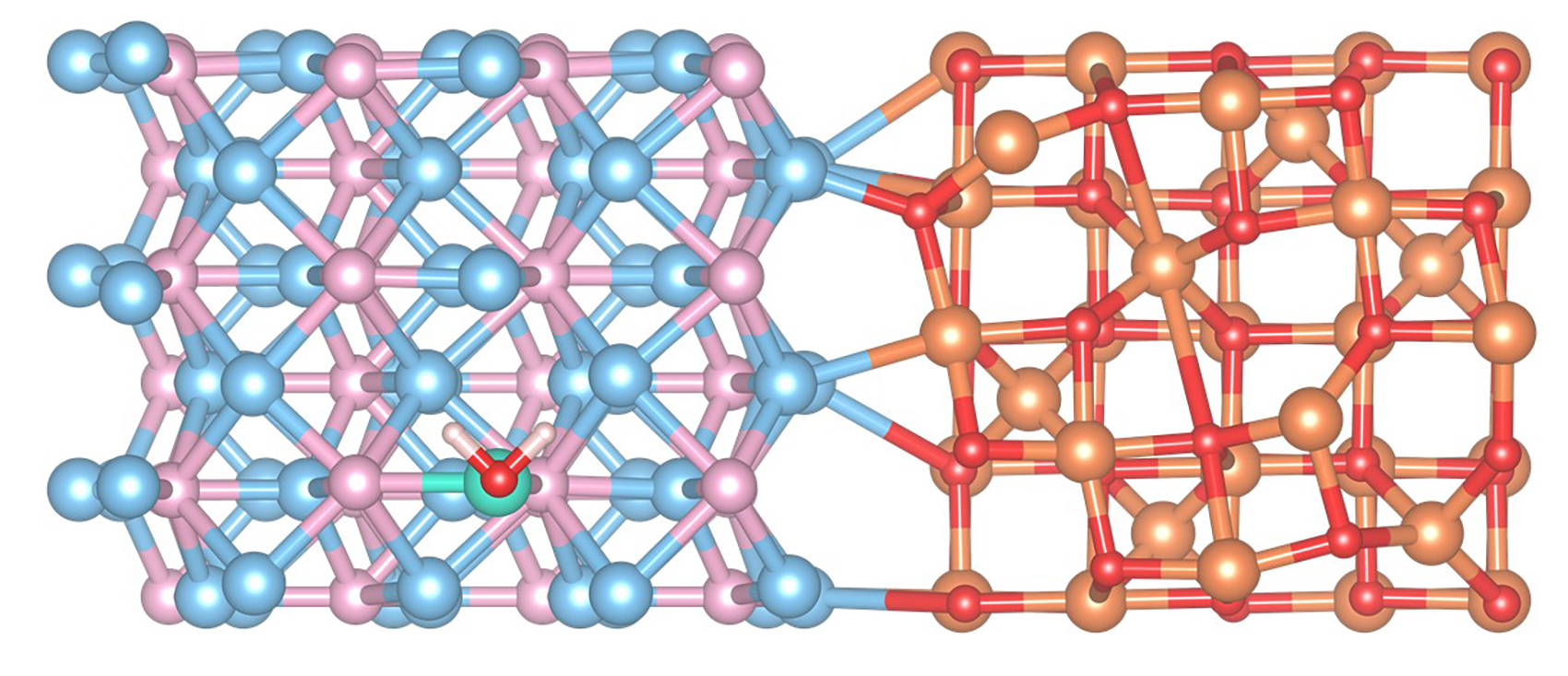 | 0.29 | 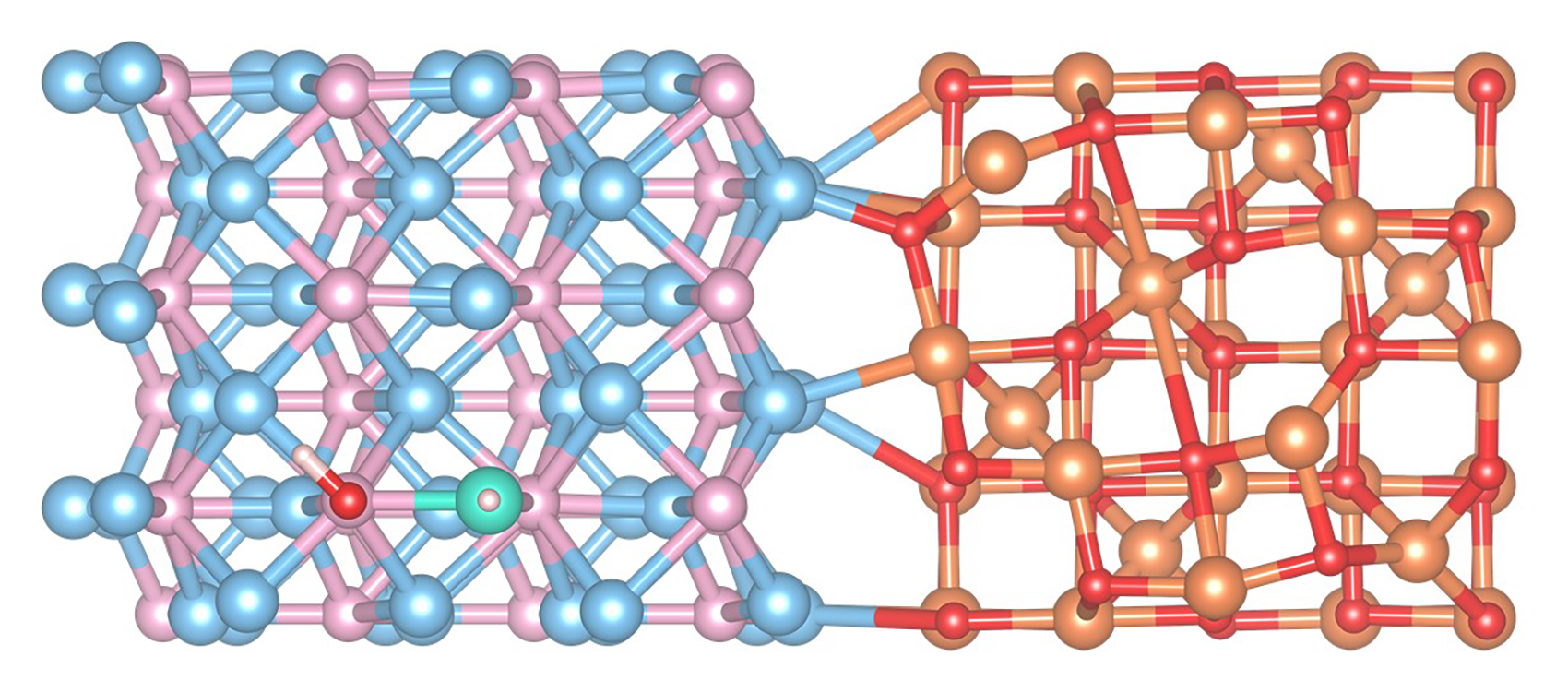 |


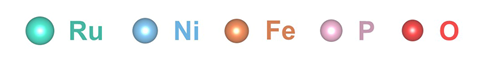


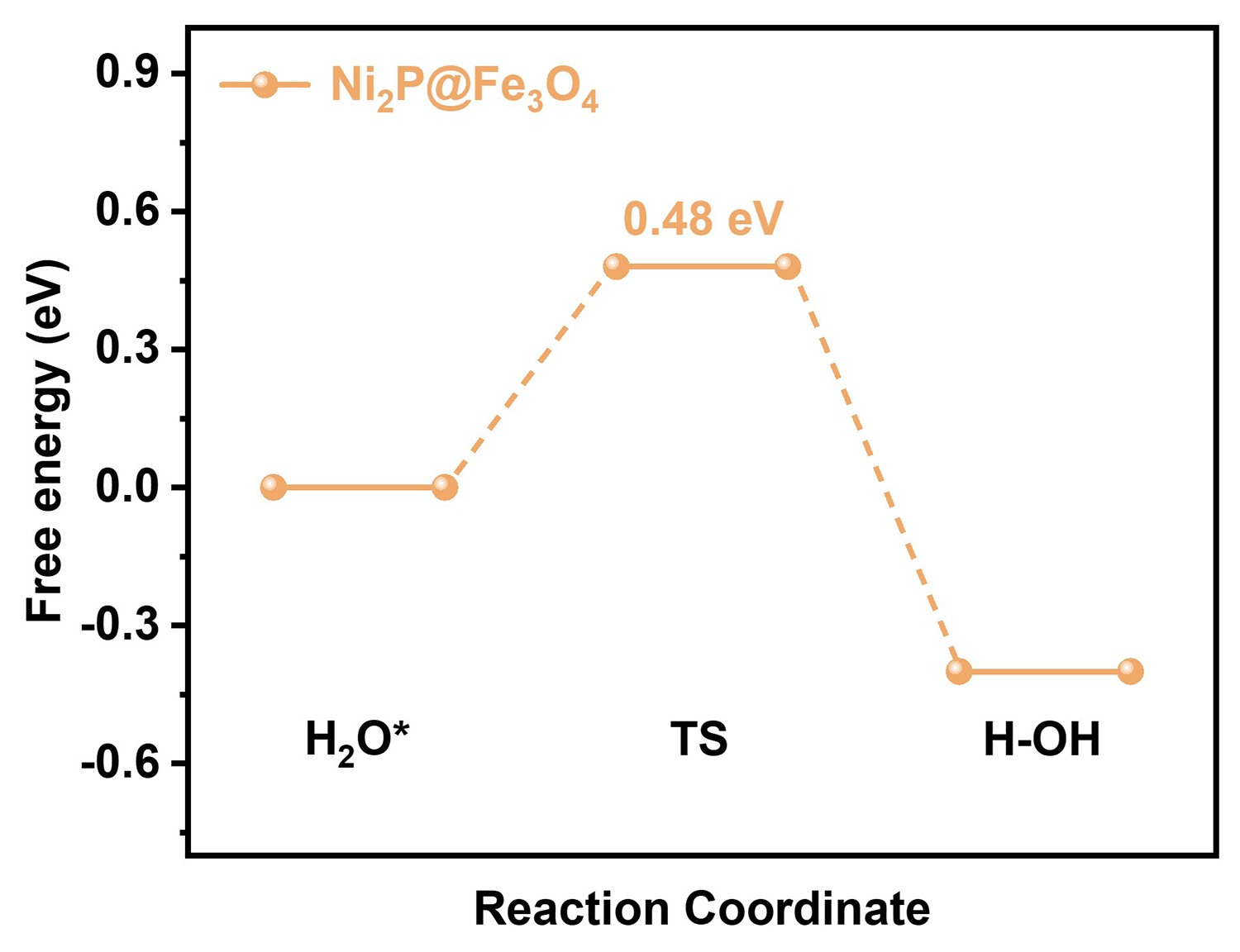


**Figure S32.** Relative energy profile of water dissociation on Ni sites of Ni_2_P@Fe_3_O_4_ surface.


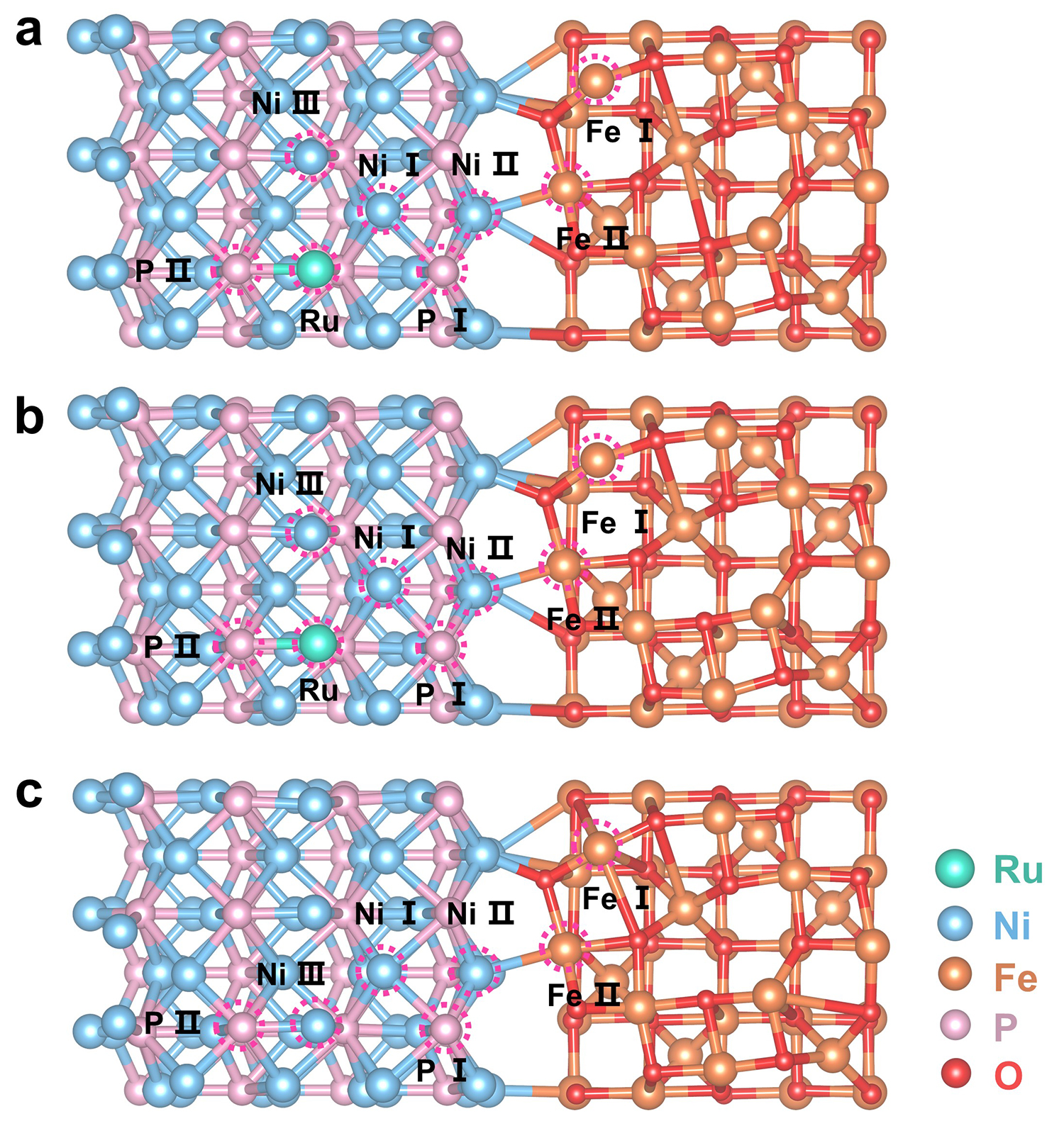


**Figure S33.** H* adsorption modeling of different sites for a) Ru_SAs_/Ni_2_P@Fe_3_O_4_−0.3 T, b) Ru_SAs_/Ni_2_P@Fe_3_O_4_, and c) Ni_2_P@Fe_3_O_4_, respectively.


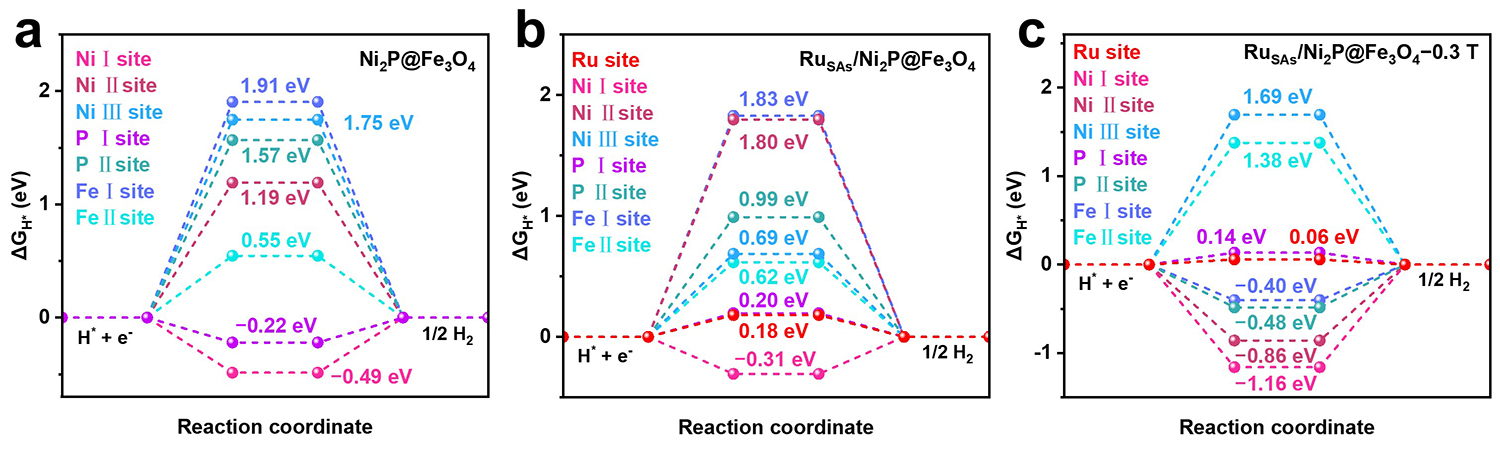


**Figure S34.** Gibbs free energy of adsorbed H* (ΔG_H*_) of a) Ni_2_P@Fe_3_O_4_, b) Ru_SAs_/Ni_2_P@Fe_3_O_4_, and c) Ru_SAs_/Ni_2_P@Fe_3_O_4_−0.3 T on different adsorption sites.


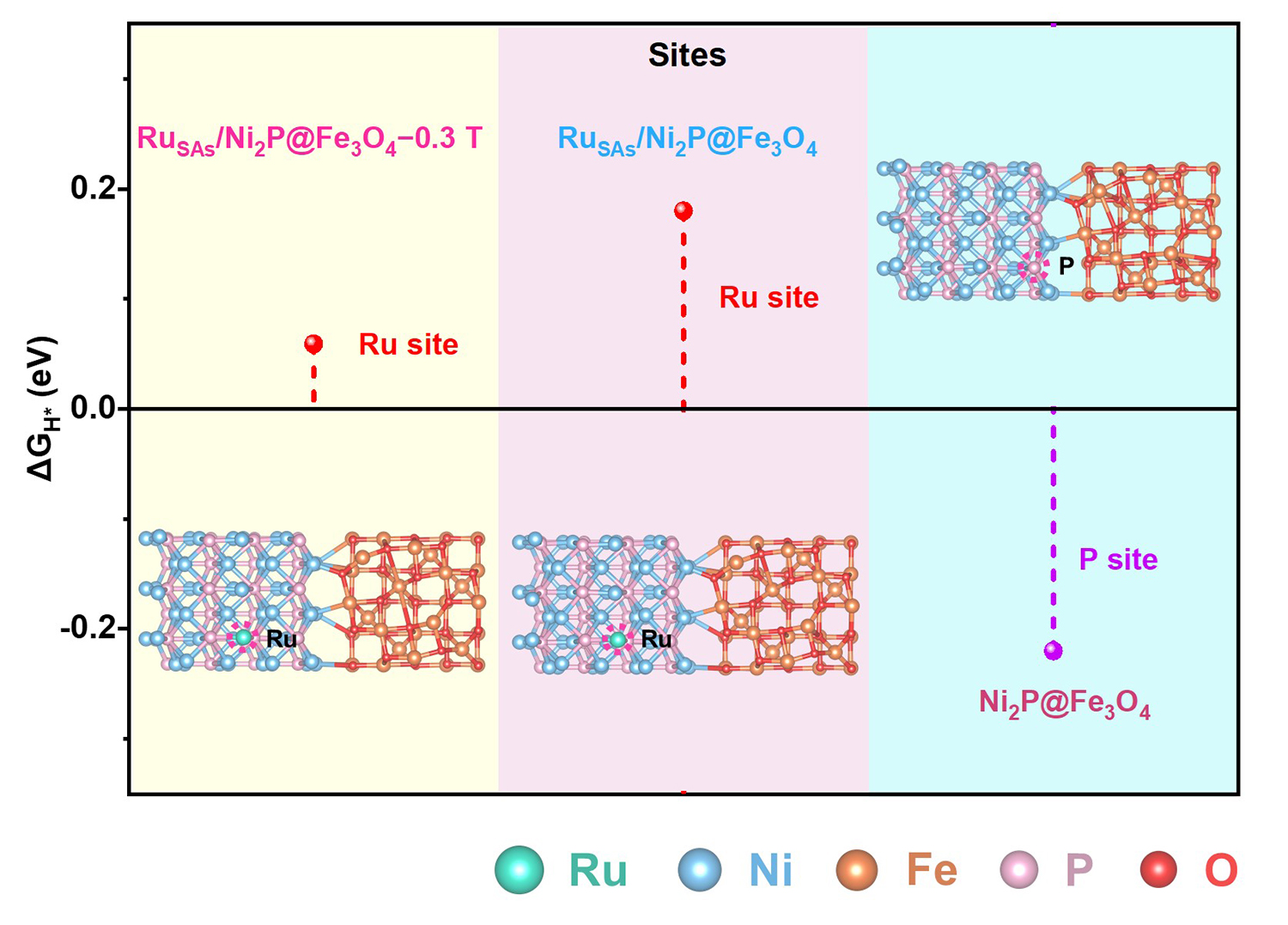


**Figure S35.** ΔG_H*_ of the active site in Ru_SAs_/Ni_2_P@Fe_3_O_4_−0.3 T, Ru_SAs_/Ni_2_P@Fe_3_O_4_, and Ni_2_P@Fe_3_O_4_, respectively.


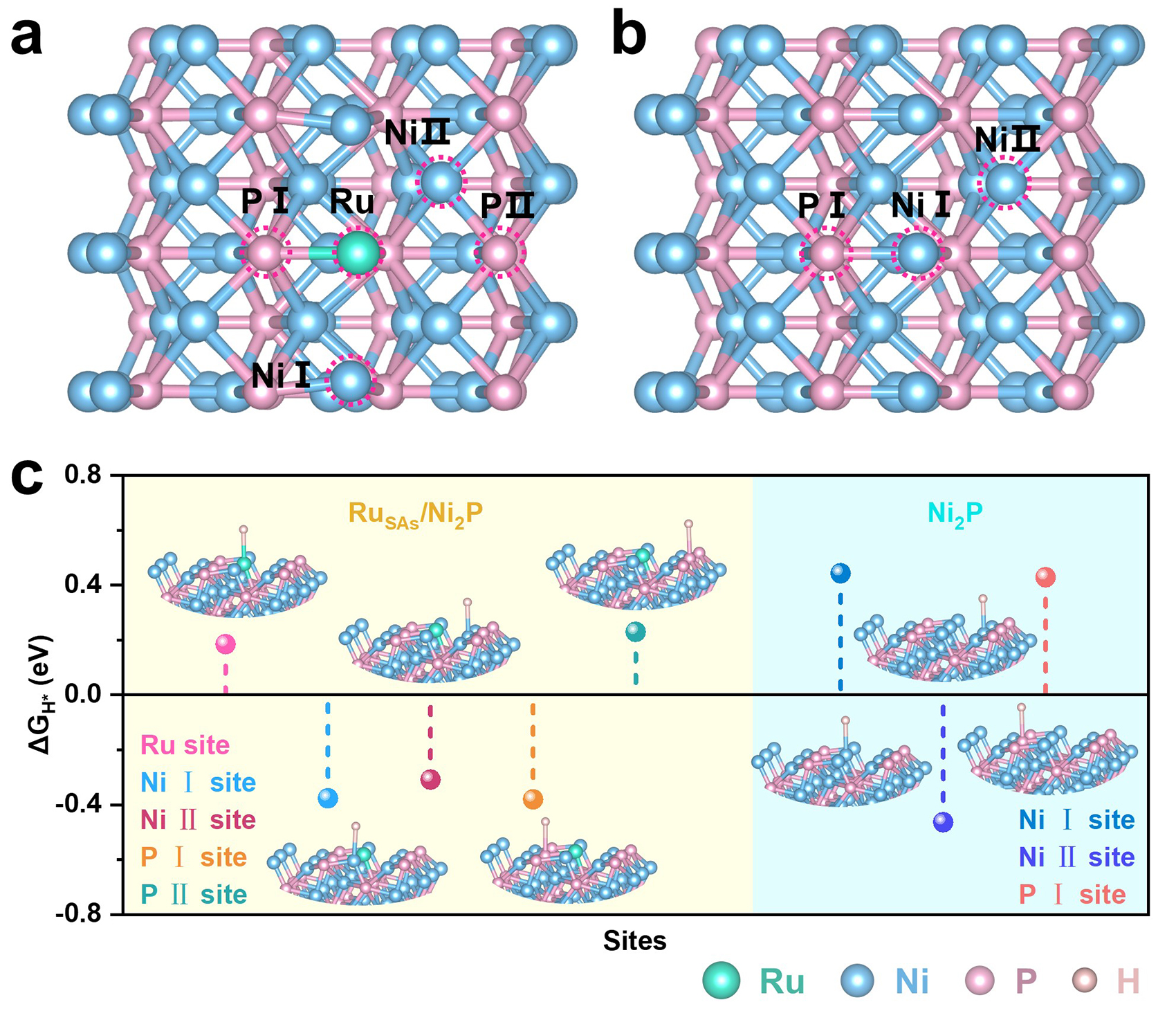


**Figure S36.** H* adsorption modeling of different sites for a) Ni_2_P and b) Ru_SAs_/Ni_2_P, respectively. c) ΔG_H*_ of active sites in Ni_2_P and Ru_SAs_/Ni_2_P.


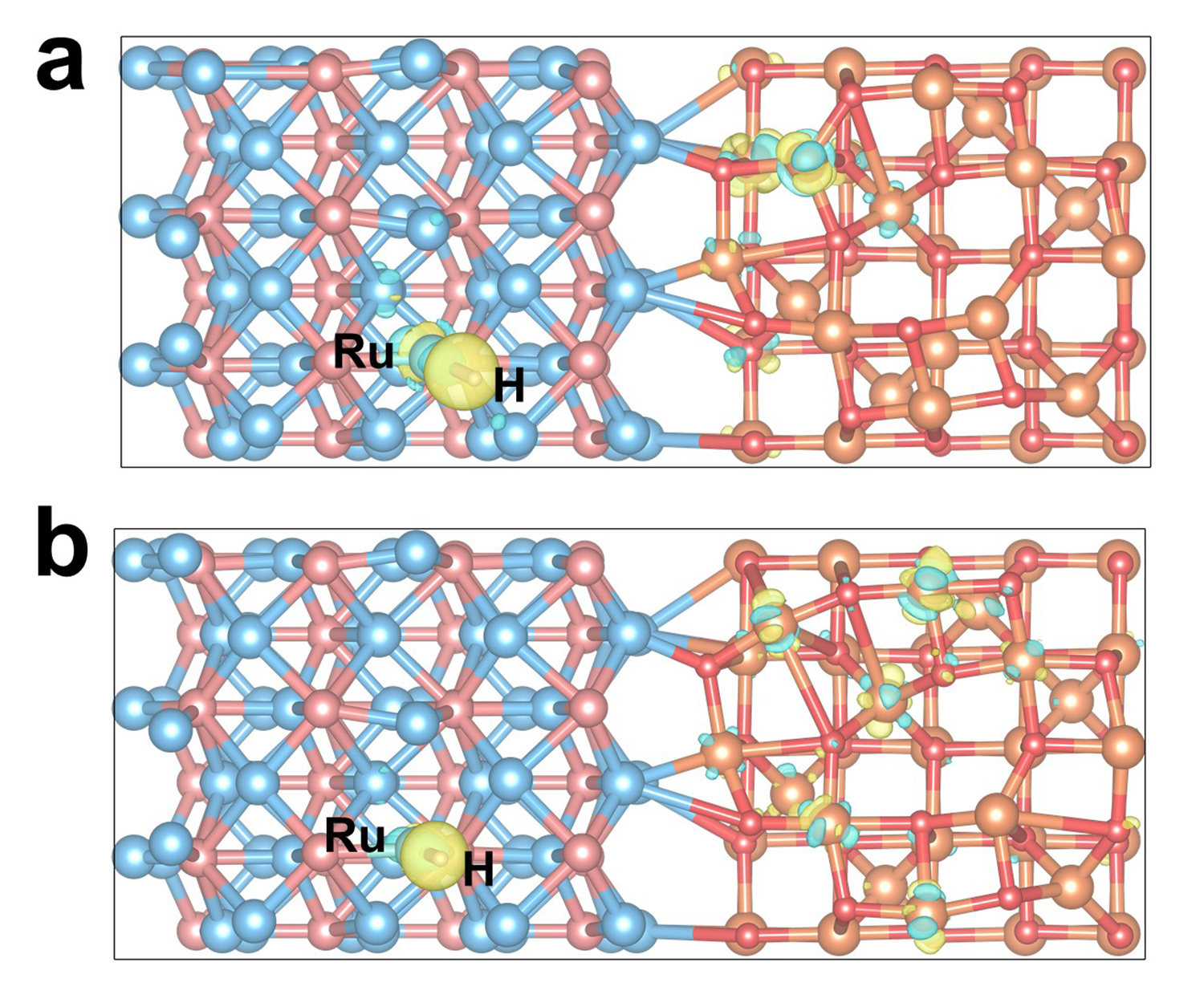


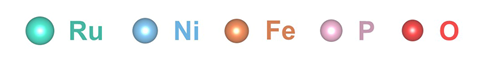


**Figure S37.** Three-dimensional charge density of adsorbed *H onto Ru site of a) Ru_SAs_/Ni_2_P@Fe_3_O_4_−0.3 T and b) Ru_SAs_/Ni_2_P@Fe_3_O_4_. (The isosurface value of the plot is 0.008 e bohr^−3^).

**References**

[1] P. Wang, K. Wang, Y. Liu, H. Li, Y. Guo, Y. Tian, S. Guo, M. Luo, Y. He, Z. Liu, S. Guo, Dual-Type Ru Atomic Sites for Efficient Alkaline Overall Water Splitting, *Adv. Funct. Mater.* **2024**, *34*, 2316709.

[2] J. Zhao, H. Guo, Q. Zhang, Y. Li, L. Gu, R. Song, Trace Ru Atoms Implanted into a Ni/Fe-Based Oxalate Solid-Solution-Like with High-Indexed Facets for Energy-Saving Overall Seawater Electrolysis Assisted by Hydrazine, *Appl. Catal. B: Environ.* **2023**, *325*, 122354.

[3] Y. Xu, R. Wang, J. Wang, Y. Zhang, T. Jiao, Encapsulation of Fe-CoP with P, N-Co-Doped Porous Carbon Matrix as a Multifunctional Catalyst for Wide Electrochemical Applications, *J. Energy Chem.* **2022**, *71*, 36-44.

[4] J. Z. Zhang, Z. Zhang, H. B. Zhang, Y. Mei, F. Zhang, P. X. Hou, C. Liu, H. M. Cheng, J. C. Li, Prussian-Blue-Analogue-Derived Ultrathin Co_2_P‑Fe_2_P Nanosheets for Universal-pH Overall Water Splitting, *Nano Lett.* **2023**, *23*, 8331-8338.

[5] F. Shahbazi Farahani, M. S. Rahmanifar, A. Noori, M. F. El-Kady, N. Hassani, M. Neek-Amal, R. B. Kaner, M. F. Mousavi, Trilayer Metal-Organic Frameworks as Multifunctional Electrocatalysts for Energy Conversion and Storage Applications, *J. Am. Chem. Soc.* **2022**, *144*, 3411-3428.

[6] C. Feng, M. Lv, J. Shao, H. Wu, W. Zhou, S. Qi, C. Deng, X. Chai, H. Yang, Q. Hu, C. He, Lattice Strain Engineering of Ni_2_P Enables Efficient Catalytic Hydrazine Oxidation-Assisted Hydrogen Production, *Adv. Mater.* **2023**, *35*, e2305598.

[7] C. Liu, B. Sheng, Q. Zhou, Y. Xia, Y. Zou, P. J. Chimtali, D. Cao, Y. Chu, S. Zhao, R. Long, S. Chen, L. Song, Manipulating d‑Band Center of Nickel by Single-Iodine-Atom Strategy for Boosted Alkaline Hydrogen Evolution Reaction, *J. Am. Chem. Soc.* **2024**, *146*, 26844-26854.

[8] C. Pei, M. C. Kim, Y. Li, C. Xia, J. Kim, W. So, X. Yu, H. S. Park, J. K. Kim, Electron Transfer-Induced Metal Spin-Crossover at NiCo_2_S_4_/ReS_2_ 2D-2D Interfaces for Promoting pH-Universal Hydrogen Evolution Reaction, *Adv. Funct. Mater.* **2022**, *33*, 2210072.

[9] Q. Fu, L. W. Wong, F. Zheng, X. Zheng, C. S. Tsang, K. H. Lai, W. Shen, T. H. Ly, Q. Deng, J. Zhao, Unraveling and Leveraging in Situ Surface Amorphization for Enhanced Hydrogen Evolution Reaction in Alkaline Media, *Nat. Commun.* **2023**, *14*, 6462.

[10] X. Teng, Z. Wang, Y. Wu, Y. Zhang, B. Yuan, Y. Xu, R. Wang, A. Shan, Enhanced Alkaline Hydrogen Evolution Reaction of MoO_2_/Ni_3_S_2_ Nanorod Arrays by Interface Engineering, *Nano Energy* **2024**, *122*, 109299.

[11] H. Sun, B. Yao, Y. Han, L. Yang, Y. Zhao, S. Wang, C. Zhong, J. Chen, C. P. Li, M. Du, Multi-Interface Engineering of Self-Supported Nickel/Yttrium Oxide Electrode Enables Kinetically Accelerated and Ultra-Stable Alkaline Hydrogen Evolution at Industrial-Level Current Density, *Adv. Energy Mater.* **2024**, *14*, 2303563.

[12] Z. Chen, X. Li, J. Zhao, S. Zhang, J. Wang, H. Zhang, J. Zhang, Q. Dong, W. Zhang, W. Hu, X. Han, Stabilizing Pt Single Atoms through Pt-Se Electron Bridges on Vacancy-Enriched Nickel Selenide for Efficient Electrocatalytic Hydrogen Evolution, *Angew. Chem. Int. Ed.* **2023**, *62*, e202308686.

[13] W. Zeng, Z. Jiang, X. Gong, C. Hu, X. Luo, W. Lei, C. Yuan, Atomic Magnetic Heating Effect Enhanced Hydrogen Evolution Reaction of Gd@MoS_2_ Single-Atom Catalysts, *Small* **2023**, *19*, e2206155.

[14] Y. Wang, Y. Shang, Z. Cao, K. Zeng, Y. Xie, J. Li, Y. Yao, W. Gan, Highly Efficient, Field-Assisted Water Splitting Enabled by a Bifunctional Ni_3_Fe Magnetized Wood Carbon, *Chem. Eng. J.* **2022**, *439*, 135722.

[15] L. Cai, J. Huo, P. Zou, G. Li, J. Liu, W. Xu, M. Gao, S. Zhang, J. Q. Wang, Key Role of Lorentz Excitation in the Electromagnetic-Enhanced Hydrogen Evolution Reaction, *ACS Appl. Mater. Interfaces* **2022**, *14*, 15243-15249.
